# Recent Progress, Challenges, and Prospects in Two-Dimensional Photo-Catalyst Materials and Environmental Remediation

**DOI:** 10.1007/s40820-020-00504-3

**Published:** 2020-08-15

**Authors:** Karim Khan, Ayesha Khan Tareen, Muhammad Aslam, Rizwan Ur Rehman Sagar, Bin Zhang, Weichun Huang, Asif Mahmood, Nasir Mahmood, Kishwar Khan, Han Zhang, Zhongyi Guo

**Affiliations:** 1 School of Electrical Engineering and Intelligentization, Dongguan University of Technology (DGUT), Dongguan, 523808 Guangdong People’s Republic of China; 2 Institute of Microscale Optoelectronics, Collaborative Innovation Centre for Optoelectronic Science and Technology, Key Laboratory of Optoelectronic Devices and Systems of Ministry of Education and Guangdong Province, College of Physics and Optoelectronic Engineering, Shenzhen Key Laboratory of Micro-Nano Photonic Information Technology, Guangdong Laboratory of Artificial Intelligence and Digital Economy (SZ), Shenzhen University, Shenzhen, 518060 People’s Republic of China; 3 College of Materials Science and Engineering, Shenzhen University, Shenzhen, 518060 People’s Republic of China; 4 Government Degree College Paharpur, Gomel University, Dera Ismail Khan, K.P.K Islamic Republic of Pakistan; 5 School of Materials Science and Engineering, Jiangxi University of Science and Technology, Jiangxi, 341000 People’s Republic of China; 6 School of Chemical and Bio-Molecular Engineering, The University of Sydney, Sydney, NSW 2006 Australia; 7 School of Engineering, The Royal Melbourne Institute of Technology (RMIT) University, Melbourne, VIC Australia; 8 Research Laboratory of Electronics (RLE), Massachusetts Institute of Technology (MIT), Cambridge, MA USA

**Keywords:** Two-dimensional materials, Photo-catalysts, H_2_O_2_/H_2_-production, Pollutant degradation, CO_2_ reduction

## Abstract

The successful photo-catalyst library gives significant information on feature that affects photo-catalytic performance and proposes new materials. Competency is considerably significant to form multi-functional photo-catalysts with flexible characteristics. Since recently, two-dimensional materials (2DMs) gained much attention from researchers, due to their unique thickness-dependent uses, mainly for photo-catalytic, outstanding chemical and physical properties. Photo-catalytic water splitting and hydrogen (H_2_) evolution by plentiful compounds as electron (e^−^) donors is estimated to participate in constructing clean method for solar H_2_-formation. Heterogeneous photo-catalysis received much research attention caused by their applications to tackle numerous energy and environmental issues. This broad review explains progress regarding 2DMs, significance in structure, and catalytic results. We will discuss in detail current progresses of approaches for adjusting 2DMs-based photo-catalysts to assess their photo-activity including doping, hetero-structure scheme, and functional formation assembly. Suggested plans, e.g., doping and sensitization of semiconducting 2DMs, increasing electrical conductance, improving catalytic active sites, strengthening interface coupling in semiconductors (SCs) 2DMs, forming nano-structures, building multi-junction nano-composites, increasing photo-stability of SCs, and using combined results of adapted approaches, are summed up. Hence, to further improve 2DMs photo-catalyst properties, hetero-structure design-based 2DMs’ photo-catalyst basic mechanism is also reviewed.

## Highlights

- Current progress in preparations, structures, and physicochemical properties of two-dimensional photo-catalyst materials and environmental remediation.
- Propose approaches of diverse of two-dimensional photo-catalyst materials-based nanoplatforms, optimization strategies to enhance activity, and their diverse applications.
- Current challenges and potential advancement of the emerging of two-dimensional photo-catalyst materials.

## Graphical abstract

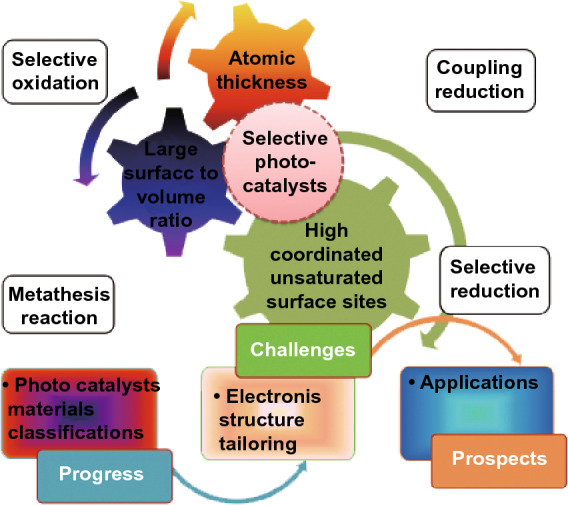

## Introduction

The sustainable energy and chemical supplies demands are very essential for modern society that is necessary for our transportation, prosperity, and daily simplicity. The world’s almost 85% energy demands are fulfilled by fossil fuels-based energy production. Therefore, an increasing progress in modern society for pollution-free energy production gains attention of the researchers in all fields. In the past, larger part of worldwide energy formation was based on fossil fuel, which increases environment pollution and hence causes global warming [1–14]. In this modern society, development in different industries causes rapid population growth, which is further estimated to increase by two factors: the current energy required by 2050 to run industries around globe and their household uses [2–4, 15–26]. Presently, world’s energy supplies are mainly reliant on fossil fuels, for example, coal, petroleum, and natural gases, which are quickly being spent. Utilization of fossil fuels will certainly cause particular gases’ emission which are very injurious to the environment. Consequently, innovative findings in science and engineering are proceeded to address the barriers for efficient energy formation and environmental safety. Hence, production of the sustainable/renewable energy is a solution to meet up the rising worldwide energy demand and especially to solve environmental pollution issues [2–4, 27]. Conversely, fossil fuel-based energy is also widely used in chemical production on industrial level via inorganic/organic transformations by applying high-temperature/pressure circumstances. Although fossil fuel supplies are possibly sufficient for some next generations, durable cost of fossil fuels is undesirable because of un-sustainability of fossil fuels recognized as partial assets and rising greenhouse gases certified to enormous release of CO_2_-like hazard gases. The technical challenges to increase an industrially talented chemical process to protect a clean, renewable energy and to reduce harmful ecological impact are connected with the use of fossil fuels [2, 3, 19, 22]. In this regard, renewable sustainable energy production is one of the significant solutions, especially hydrogen (H_2_)-based energy creation by photo-catalysts as well as electrocatalysts [2–4, 18, 19, 21, 25]. Here, we will mainly concern on photo-catalysts. The H_2_ has the maximum energy contented per weight in combustion fuels and manufactures simply water (H_2_O) as by-product [3, 4, 19]. Thus far, straightforward transfer of solar energy to fuel energy (H_2_) and chemical energy was viewed as one of the green renewable ways to deal with energy and environmental pollution issues in the future [2–4, 17, 18, 21, 23–25]. Hence, H_2_ is considered as an ultra-clean, powerful, environment friendly, and hopeful another choice for meeting the future fuel necessities with environmental safety by less release of greenhouse gasses [2–4, 17–19, 21, 23, 24].

## Basic Properties of Photo-catalysis

### Merits of Photo-catalysis

The considerable reliance of worldwide economy on non-renewable and geopolitical susceptible fossil fuel energies has led to necessity in advance technologies to protect alternative clean and renewable energy supplies. In between different renewable energy sources (i.e., wind, tidal, hydroelectric, ocean currents, biomass, geothermal, and solar), solar energy is by far the most abundant, low cost, pollution free, and sustainable. Even though the total solar energy the earth receives for one hour is greater compared to annual global energy expenditure, the most serious challenge remains collection and storage of this very diffuse form of energy to facilitate real-world application and non-interrupted fuel supply. Photo-catalysis can be basically explained as a method wherein photo-generated electrons (e^−^s) and holes (h^+^s) induce targeted redox reactions on light absorbers and/or co-catalysts loaded on it. A range of other invented renewable energy schemes, semiconductor (SC)-based photo-catalysis, in which infinite and clean solar energy can be acquired as a possible technology [28] achieved great interdisciplinary concentration for their various probabilities in energy and environmental uses. Efficient transformation of solar energy to solar fuel using photo-catalytic method was measured as very eventual enduring maneuver to resolve global energy and environmental concerns [29]. Naturally abundant sunlight and H_2_O splitting-based production of H_2_ by using sunlight were verified as regenerative, environment friendly, and vast techniques to resolve energy disaster and environmental pollution. In photo-catalysis method, a steady and capable photo-catalyst is an important aspect to attain a high efficiency of H_2_. For energy crises as well as environmental issues, SC-based photo-catalysis has enormous ability to guarantee long-lasting and sustainable development, because of direct consumption of green solar energy for formation of important H_2_ fuels and degradation of organic pollutants. Generally, four steps take place in the photo-catalytic process:

1. Light absorption
2. Creation of photo-generated (e^−^–h^+^)-pairs
3. Movement and recombination of photo-generated (e^−^–h^+^)
4. Redox reactions at photo-catalysts surface

How to understand it proficiently is very demanding, both kinetically and thermodynamically. The complexities lie in the subsequent features:

1. Maximum yielding of solar energy (mainly visible (vis) light) to produce enough energetic e^−^s/h^+^s
2. High mobility and long dispersion length of photo-generated e^−^s/h^+^s to suppress bulk recombination
3. Sufficiently strong reduction power of photo-generated e^−^s and h^+^s to persuade reactions, specially H_2_O oxidation that demands four e^−^s
4. Plentiful surface locations for forward target reactions as an alternative of back reactions (e.g., H_2_ and O_2_ reaction to fabricate H_2_O)

Such four subjects represent a significant research pathway. Moreover, three steps span a huge timescale from 10^−15^ to 10^−1^ s. What’s more demanding are intrinsic conflicts between necessities for three key steps. Minimum three factors are considered here:

1. Increasing light absorption range (reduced bandgaps) generally leads to small reduction capability of photo-generated e-s and/or a lower oxidation capability of photo-generated h^+^s;
2. The very low mobility of h^+^s compared to e-s in most SCs does not support rate-determining H_2_O oxidation reactions,
3. The difference in random distributions of oxidation and reduction reaction sites and required migration of e^−^s/h^+^s in diverse directions.

The strong underlying conflicts connected with photo-physical process, electronic properties, and catalysis principles build recognition of highly efficient photo-catalysis as a very challenging process. To solve these challenges, it is significantly important to accurately control every fundamental step depending on a comprehensive consideration of photo-catalysis and structure property interactions. Therefore, first we are going to explain SC materials’ suitability for photo-catalytic nature.

### General Selection of SC Photo-catalytic Materials

Generally, the photo-catalysis is an accelerated photo-reaction method in existence of SC photo-catalyst, in which photons with energy *hv* ≥ *E*_g_ (*E*_g_ = band gap (BG) energy) of photo-catalyst are adsorbed to photoexcite free electrons (e^−^s) to conduction band (CB), creating holes (h^+^s) in valence band (VB). Photo-generated (e^−^–h^+^)-pairs participate in an important part for solar energy transfer method, for example, solar H_2_O splitting, CO_2_ reduction, and photo-catalytic pollutant degradation. Although photo-generated carriers in excited states are less stable, they recombine easily, which results in low conversion effectiveness of photo-catalysis [30]. By the way, since discovery of photo-catalytic H_2_O splitting with TiO_2_ in 1972, great effort was applied in progress of photo-catalysts for an efficient photo-catalytic method [31]. The SC-based photo-catalysis concerned huge research attention, [32] since it was considered very novel solution to manage energy deficiency and environmental pollution problems [33]. The sunlight as an exterior driving force can split H_2_O into H_2_ and O_2_, reduce CO_2_ to chemical and valuable fuel, as well as terminate pollutants entirely [34]. Normally, the main significant efforts in the photo-catalytic development are categorized as light absorption, charge separation, transfer, and surface redox responses. By irradiation of photo-catalysts, it absorbed sunlight, which excites to produce (e^−^–h^+^)-pairs, when *hv* ≥ *E*_g_, leaving e-s in CB and h^+^s in VB, respectively. After that, photo-generated e^–^s and h^+^s are diffused to material surface and also transferred to surface active sites, prior to connection with surface reactions. Sometimes, charge carrier’s recombination occurs and crystal structure, particle size, crystallinity, surface morphology, etc., strongly influenced separation efficiency. At last, target molecules are adsorbed on the surface of materials and experience charge addition development and desorption to make final results [35].

In between (e^−^–h^+^)-recombination process, unnecessary heat is created, which causes a negative role in photo-catalytic production. In the photo-catalysis method, a stable and efficient photo-catalyst is an imperative feature to get high efficiency of H_2_. Additionally, the driving force of solar light photo-catalysis demands suitable SCs to perform various photo-catalytic responses, for example, H_2_O splitting to manufacture O_2_ and H_2_, CO_2_ reduction to hydrocarbon fuels, degradation of organic pollutants, disinfection of bacteria, and selective formation of organic compounds [36]. The milestone occurrence of photo-catalytic H_2_O splitting, by TiO_2_-based electrodes in an ultraviolet (UV) light, was started from revolutionary research co-authored by Fujishima and Honda [37]. In 1976, photo-catalytic degradation of organic contaminants was studied by Carey et al. [38] using TiO_2_ in aqueous suspension. In 1979, Inoue and co-authors examined photo-catalytic reduction of CO_2_ to a range of organic compounds by SC materials, for example, TiO_2_, SiC, ZnO, CdS, and GaP, in aqueous solution. After that, various considerable progresses were made in the formation of very proficient SC-based photo-catalysts. Up to now, several SC photo-catalysts were exploited and utilized in H_2_O splitting. Based on composition, photo-catalysts are usually classified into three kinds:

1. Metal oxides (MOs)
2. Metal chalcogenides
3. Metal-free photo-catalysts

So far, hundreds of SC materials are discovered for different photo-catalytic uses by tuning a range from composition, electronic, and crystal structure. While important accomplishment was achieved in optimizing photo-catalytic performance, most photo-catalysts still suffer from relatively low photo-catalytic efficiencies that are much lower compared to the necessities for probable realistic uses. Based on previous research investigation, probable UV–Vis-active and vis-light-active photo-catalysts included TiO_2_, ZnO, Fe_2_O_3_, CdS, Bi_2_WO_6_, BiVO_4_, Ta_2_O_5_, Ta_3_N_5_, TaON, C_3_N_4_, and so on [39]. To date, emerging high-quality SC photo-catalyst for surmount recovery of energy deficiency and environmental hazards is a great research field [36]. Despite quick progress of conventional photo-catalysts, they are still facing numerous major challenges:

1. Many SCs, particularly MOs, can absorb UV light because of their wide BG [40]
2. A few SCs are not appropriate for entire H_2_O splitting, due to their inappropriate band location and because they only show either H_2_O oxidation or reduction activity [41]
3. In relocation of photo-generated charge carriers to surface reactive sites, charge recombination happens simply for bulk and on photo-catalysts’ surface [42]
4. The majority of bulk SC reaction active sites cannot be exposed to surface and are utilized in the photo-catalytic process [29]

Therefore, key issues to attain an excellent photo-catalytic performance depend on normal mean of high-efficiency photo-catalysts. In recently discovered new potential photo-active materials, 2DMs got much consideration. Bearing in mind various characteristics and advantages, the promising 2DMs with suitable energy band configuration can stimulate new visions [14, 43, 44]. Recent research in 2DMs has advanced the modernized attention in p–n junction; the oldest electrical mechanism was employed in electronics and optoelectronics devices research. The 2DMs offer an amazing flexibility to propose a novel (p-n)-junction device configuration, not workable through usual 3D bulk SCs. The 2DMs signify a promising category of materials that have NSs-like configuration with thickness of just one or few atoms [45]. Attempts were ignited through innovation of graphene (G) in 2004, a single-layer (SL) carbon material along outstanding thermal, mechanical, and electrical characteristics [46]. Ever since, a range of G-like 2D photo-catalysts were become a relevant topic in photo-catalysis field. The 2D photo-catalysts showed special chemical and physical properties in contrast to their bulk counterparts. Emerging 2DMs with unique structural and electronic properties and appropriate band structure have showed huge potential of achieving the desired photo-catalytic efficiency. There are numerous features which influence the photo-catalytic efficiency of photo-catalysts, e.g., composition, BG, crystallinity, surface state, and morphology of SC materials, and interfacial properties of components for composite photo-catalysts.

**Fig. 1:**
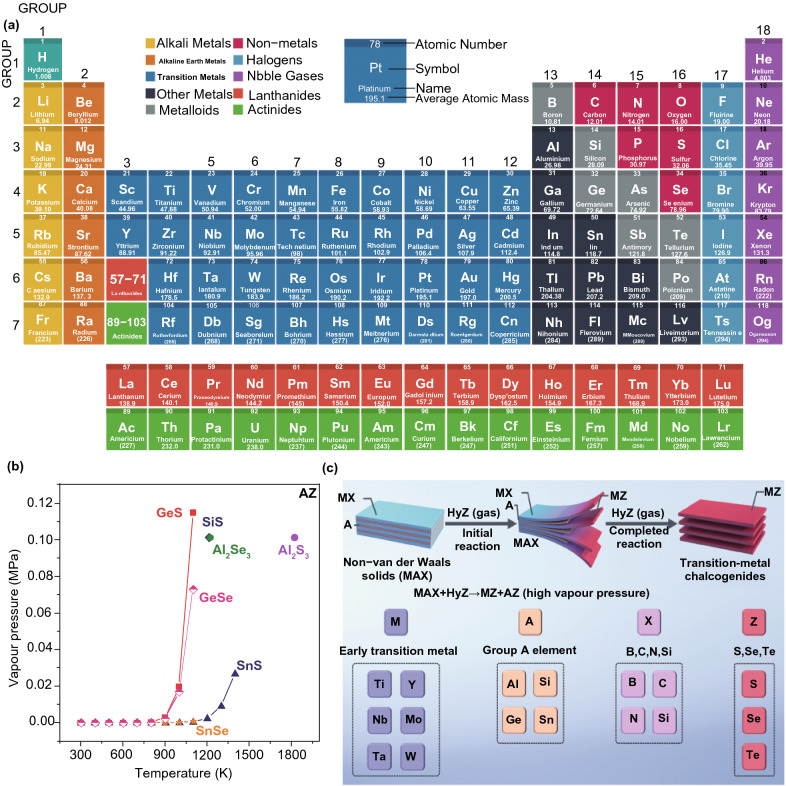
**a** Periodic table shows discovered 2DMs. **b** Temperature and vapor pressure relations for a variety of AZ substances. **c** Scheme for conversion of non-vdWs solids to 2D vdWs TMDCs, where non-vdWs solids like MAX phases are gradually transferred to 2D TMDCs through a topological conversion response (MAX + HyZ (gas) → AZ + MZ), related to volatile AZ products. Adapted with permission from Ref. [48]

In view of necessities for competent light adsorption and photo-generated carrier separation and transport, if possible, a photo-catalyst must contain an elevated specific surface area (SSA), good crystalline structure, stability, and an appropriate band structure [29]. The 2DMs arrangement can supply huge SSA and a large fraction of low coordinated surface atoms to produce further UV light, whereas photon absorption in bulk materials or nanoparticles (NPs) is frequently inadequate through transmittance of light and reflection at grain boundaries [47]. Furthermore, as a result atomic size thickness significantly decreases the migration distance; charge carriers produced in 2DMs interior will be quicker to transfer on surface compared to bulk materials (Fig. 1a). It will significantly decrease recombination chance of photo-generated carriers and support photo-catalytic method. Finally, regarding surface redox reactions, distinctive 2DMs configuration along elevated ratio of surface atoms to whole atoms can cause new SAS to speed up the reaction development. Additionally, atomic breakdown energy develops into comparatively minute when thickness decreased to atomic level and so additional surface defects will come into view. These surface defects will promote and improve target molecule adsorption to make strong interaction, easy charge transfer, and better activation procedure. Photo-catalysts with such characteristics and 2DMs configuration receive high interest, and a great number of related studies were performed [32].

To concentrate on these challenges, formations of new and more competent photo-catalysts are required to energetically investigate in this field [49]. The 2D structures along with foreign electronic properties and a high SSA are formed from layered [50] and non-layered [48] materials. The layered materials are identified through strong in-plane bonds and weak van der Waals (vdWs) force in layers. On the other hand, Ajayan and co-workers [48] recently discovered an efficient formation approach via the progressive conversion of non-vdW solids to 2D vdWs transition metal chalcogenide (TMDCs) layers with recognized 2H (trigonal prismatic)/1T (octahedral) segments (Fig. 1b, c). Conversions, obtained after exposing non-vdWs solids to chalcogen vapors, were controlled utilizing enthalpies and reaction products vapor pressures. Heteroatoms-substituted (e.g., phosphorus and yttrium) TMDCs were also formed by the same scheme, so a general formation scheme is allowed to form phase-selected TMDCs’ 2D configurations with excellent stability at elevated temperatures (about 1373 K) and obtain scalable manufacture of SLs. These 2D TMDCs have wide uses in catalysis, electronics, and energy storage applications. On account of remarkable structure-based, chemical and physical, properties of 2DMs, the construction of few-layer (FL) or single-layer (SL) 2DMs provokes broad attention as talented photo-catalysts with numerous benefits:

1. The BG and light absorption of 2D-SC can adjust via altering layers number [51].
2. The (e^−^–h^+^)-recombination in case of bulk can decrease because of atomic size of 2DMs [52].
3. The SSA of the SCs is significantly enhanced, and most of the SASs can be exposed at surface and included in photo-catalytic reaction [47].

Along with the different 2D photo-catalysts with only FLs or SL structure, G-based photo-catalysts, 2D oxides, 2D chalcogenides, 2D graphitic carbon nitride (g-C_3_N_4_), and other 2D-SCs started gaining huge attention in photo-catalysis [49]. Although 2D photo-catalysts are viewed as talented materials to exchange solar energy into chemical energy as H_2_-formation, there are a number of hurdles, which limit their uses [49], as follows:

1. The exciton binding energy in 2DMs-based photo-catalysts was significantly enhanced because of smaller unfavorable e^−^-screening than bulk material [53].
2. Some 2DMs-based SCs are not stable in aqueous solution or air; thin-layer 2D SC can be assembled collectively or oxidized through photo-generated h^+^s during reaction, which leads to deterioration in photo-catalytic activity [54].
3. While (e^−^–h^+^)-pairs recombination is less compared to bulk SCs, it still resides in 2D photo-catalysts [55].
4. Reduction potential and oxidation potential of few 2D-SCs are not enough in overall H_2_O splitting [55].

To address such issues, a range of approaches were designed to increase photo-catalytic activity of 2DMs-based photo-catalysts, for example, doping with a metal or nonmetal elements, inducing defects, and coupling with metal or SCs, which will be discussed in detail in the upcoming sections [56]. In fact, photo-activity of photo-catalysts relies on their properties, for instance, electron affinity, crystal structure, BG, and interface in photo-catalyst as well as co-catalyst [57]. Consequently, for well-organized transfer of H_2_O to H_2_, the mixing of photo-catalyst and co-catalyst required novel interface structure. This kind of interface can optimize absorption of light for photo-catalysts and support e^−^/h^+^ separation. Normally, bigger contact area at interface can offer enough charge transfer and trapping channels for parting (e^−^–h^+^)-pairs generated by incident light [49]. The above-mentioned problem has one another solution, which is the hetero-structure formation of 2DMs. In contrast to 0D–1D, 1D–1D, 0D–2D, and 1D–2D interfaces, 2D–2D coupled hetero-structure-based interfaces concerned broad concentration in photo-catalysis due to their particular advantages, as follows [49]:

1. The creation of intimate interface in two SCs is in support of exciton dissociation, which enhances the photo-catalytic quantum efficiency [58].
2. It is simplistic and proficient to structure the intimate interface in 2DMs-SCs, even if they have some mismatch of lattices [59].
3. Large lateral size along with high SSA leads to huge contact area in 2D/2D photo-catalysts that advance (e^−^–h^+^)-pairs’ separation and transfer [60].
4. The band potential coordinated to overall H_2_O splitting by integrating H_2_/O_2_-evolution photo-catalyst. Therefore, oxidation and reduction influence of SCs is balanced for H_2_O splitting [61].
5. Creation of 2D/2D hetero-structure is advantageous to develop stability of photo-catalyst because of increase in photo-corrosion and agglomeration [62].

As a sustainable technology, the SC photo-catalysis has gained significant attention in the recent decades due to possible ease/resolve energy and environmental pollution concerns. Therefore, due to 2D/2D interface advantages, many 2D/2D structures are formed recently to improve photo-catalytic performance of photo-catalysts [63, 64]. Based on these advantages, we are going to summarize most of the related topics, which can further improve the photo-catalytic phenomenon for H_2_O splitting (H_2_O oxidation and H_2_ evolution), CO_2_ reduction, N_2_ fixation, organic production, removal of pollutants based on G-based photo-catalysts, 2D oxides, 2D-chalcogenides, 2D g-C_3_N_4_, and some other 2D-SCs.

The 2DMs reviewed here are considered as low-dimensional materials with thickness ranging from SL to few nanometers (nm) by means of basal plane controlling total surface area, and 2DMS-based SCs photo-catalysis principles, synthesis, and stability will be briefly reviewed. Here, up-to-date development of 2DMs-based photo-catalysts is summarized, and significant evaluations of categorizing and convenient production method of 2DMs-based photo-catalysts are presented. To further boost these results, different policies to engineer electronic structure of 2DMs-based photo-catalysts are summed up, such as component tuning, thickness tuning, defect, and doping engineering. Hybridization with insertion of outside components and keeping 2D structure is explained to improve photo-catalytic efficiency, for example, quantum dots (QDs)/2DMs, single atoms/2DMs, molecular/2DMs, and 2D–2D stacking materials. Therefore, we will give a concise explanation of recently developed 2DMs, their applications in photo-catalysis, and the promising approaches for the photo-activity progress from the perspective of chemical doping, hetero-structure layout as well as functional structural design assembly. More importantly, attention will be paid to advancement of versatile photo-catalytic applications of 2DMs-based photo-catalysts in H_2_O oxidation, H_2_ evolution, CO_2_ reduction, N_2_ fixation, organic synthesis, and elimination of pollutants. Besides, manufacture approaches and characterization methods of 2D/2D photo-catalysts are also reviewed. Finally, ongoing opportunities and challenges for upcoming progress of 2DMs-based photo-catalysts in this exhilarating yet still upcoming area of research will be projected [29, 32, 49] and a short summary of present research position and challenges, with respect to 2DM-based photo-catalysts for photo-catalysis applications, will also be explained [29]. It is extremely important and insistent to present a timely updated widespread review on this matter to endorse further progress in the upcoming direction [32].

### Benchmark Photo-catalysts

As it appears that too long step is in our conviction also a little that considered in future investigations that are association derived from present price of photo-catalytic materials. This feature looks alienated to laboratory-level work, deals with elementary information, and consequently accords with the rule not to be hampered by funds restrictions. On the other hand, we must admit that exploration is increasingly related to industry, and funding is governed through financial analysis of project, so this feature can no longer be ignored. In recent publications, it is expected to go forward in claims on importance of reported materials due to the absence of precious metals that were still considered as co-catalysts [65]. It is a too common assumption that does not inform the features of cheaper material; consequently, not including a precious metal (frequently applies in small quantity) is not essentially an economic choice, because material still depends on expensive starting materials, or tiresome reaction conditions (purification, temperature, solvent, etc.) that finally end up in inflating the cost of the proposed catalyst. It may take time to endeavor a little quantitative cost estimation to propose photo-catalyst, not only because the time is a necessary feature, but also getting support for optimizations in this field from other researchers [66]. Coming to the point, the following three key components are proposed to be explored to compare the built-in photonic effectiveness of a variety of photo-catalyst materials in laboratories.

1. Incident photon flux (photon numbers with respect to wavelength per time).
2. “Optimum rate” (achieve the highest photo-catalytic rate through changing photo-catalyst quantity in a specified reactor).
3. Rate of reactant expenditure or product evolution (at optimum rate under diverse reaction conditions).

The increase in heterogeneous photo-catalysis interest and other solar fuel conversion schemes will unavoidably lead to more research in this area. Unfortunately, many research works enclose imprecision while studying photo-catalytic measurements, particularly while reporting gas evolution [67]. It is taken toward benchmark materials’ selection difficulties as there is non-reliability in efficiency measurements. In the literature, some common errors are observed in expectancy of increasing overall quality this direction. It is recommended that classification for exploring photo-catalytic rate is given as follows:

1. Reactant conversion kinetic or product formation rates.
2. Incident photon flux with respect to wavelength.
3. Activities or partial pressures of reactants and sacrificial reagents.
4. Solution type, electrolyte concentration, and pH.
5. Quantity of photo-catalyst, co-catalyst, and solution.
6. Flow rate of reactor and volume/dimension of reactor.

Following combined experiments carried out to assess photo-catalytic performance in likely comprehensive approach, the next step is to comprehend how recently studied photo-catalyst ranks in between present photo-catalysts. Benchmark cannot recommend from all considerations mentioned previously. A lot of investigations have been inadequately performed on this significant part of work, and only contrast materials are benchmark reference catalysts, for instance TiO_2_ Degussa P25. It certainly offers an early essence of photo-catalysts, but all the time comparison cannot provide a good judgment and is not enough to validate the published results. It might appear understandable, but arises a first theoretical question: Is model reported activity vs. Degussa P25 still possessed nowadays? We are bombarded with a variety of novel guidelines of editors, industries, grant agencies, and so on, that it is vital that upcoming photo-catalytic studies must concentrate on utilization of vis-light irradiation. It is a logical insight that provided strong relationship of photo-catalysis with sustainability, future realistic growth should take toward green energy, and process must hinge on utilization of sun light. A lot of energy is irradiated through solar spectrum in the range of vis spectrum (43%), but still more is in fact irradiated through IR rays (52%), yet at this moment it is complicated to utilize it for SC photo-catalysis. It is confined energy to produce requisite charge separation in the majority of SCs-based photo-catalysts. However, it is valuable to note that only some ground-breaking works on exploitation of IR radiation have come forward [68–71], that hold interesting promises for future research development. Indeed, UV region (5%) is far too little, and so there is commonsense that wide BG (≥ 3.0 eV) SCs by themselves can no longer participate in leading role and become outdated unless investigated for development of strategies built around multi-component structural arrangements, for example Z-schemes and p–n junctions. Therefore, it looks conflict that activity must be indefinitely benchmarked against a UV-active SC, i.e., Degussa P25.

In these days, state-of-the-art catalysts’ table of comparisons are emerging more frequently in research work. These are more helpful, if selected with care. Tables should not evaluate one but maximum possible activity potential factors. A comparison between QY does not reveal a lot about catalyst selectivity and stability, which are the two equal significant conditions of comparison. The experiment duration choice for calculating QY is arbitrary and thus can be simply turned to researchers’ expediency, losing objective, particularly while kinetics of product formation are not steady. Furthermore, a photo-catalyst with superior AQY or QY could be simply synthetically better, as exclude a donation to evolve product through other mechanisms working in dark. One more significant feature is that AQY experiments are generally performed with monochromatic light source, and as declared already, QY differs along excitation wavelengths. Tables of comparison completely conversed on QY preferably demand of comparison depend on polychromatic lights sources or as a minimum between photo-catalysts mainly absorbing in same narrow wavelengths range (a situation hard to attain). It is obvious to compare standard catalyst and synthetic catalysts discovered under similar catalytic conditions. Terminology is an additional feature not to be underestimated, as it can be the source of perplexity. As distinct earlier QY and AQY refer to quantity of consumed reactant (or product formation), other International Union of Pure and Applied Chemistry definitions more frequently utilized in heterogeneous photo-catalysis regarded as photonic efficiency (PE) and quantum efficiency (QE):

- QE = photochemical events/absorbed photon flux
- PE = photo-reaction rate/rate of incident photons [72]

Researchers need confirmation whether they are comparing the same factors. In general, we discourage a benchmark prepared completely for QY, PE, or QE, as in our estimation it is imperfect and deceptive [73]. Other activity data can propose extra basic information: Reporting product formation rates over unlimited time offers evaluation of catalyst stability and not very precise suggestion of probable diverse system, as well as a comparison on such terms is necessary for designing catalysts to be formed at commercial level. Compared rates should be studied for catalyst per both surface area and mass, for cause elucidated above.

## Classification of 2DMs for Photo-catalysts

Advancement in material and engineering science over the past years has allowed huge development in catalysis, sustainable energy production, sensor, and electronics. Novel spectroscopy and nano-fabrication techniques offered tools to comprehend primary materials’ properties and to materialize their functionalities by adjusting their configuration and composition. It leads to enormous advancement in multi-component industrial catalysts [73] (e.g., become weak after treatment [74]), excellent chemical production, electrocatalysis (e.g., fuel cell catalysts [75]), and photo-catalysis [76]. This growth was not only governed through turnover and market demands but also through elevating community understanding, rules for environmental safety, and sustainable growth. Currently rising sustainable development and technologies create utilization of a broad range of components, of which some are rare and unequally spread on earth and therefore have economic viability and at probable risk supply. In some way ironically, sustainability and risks associated with material are frequently ignored in academic-level investigation. It is due to functionality and performances during working conditions generally prevailed over synthesis and takes apart costs; hence, evaluation of material criticality and its viability is very intricate and basically goes ahead [77]. However, a basic point of view is materials’ sustainability for final target in renewable energy synthesis. Certainly, as clean energy is almost limitless (e.g., solar, wind), materials and chemicals utilized to transfer it to real electrical energy are obtain rarely. Notably, precious metals group (i.e., platinum), rare-earth elements, gallium, aluminum cobalt, and many others [78] are indispensable components of immensely utilized commercial catalysts. If dependence can be decreased via replacement, such materials would be recycled more competently in the future to circumvent economic disturbances and increasing reserved competition [79]. These materials should have the following properties:

1. Catalyst durability upgrading through material design (post-modifications, confinement)
2. Lowering noble/rare metal loadings, whereas upholding high activity, through maximizing active surface area (atomic-level thickness in low-dimensional materials)
3. Substituting significant components with cost-efficient and abundantly accessible ones (base metals, carbo-catalysis)
4. Enhancement of durability in catalysts’ synthesis and removal (green chemistry-based catalyst recycle)
5. Evaluation of toxicity and environmental effect of catalyst materials

**Fig. 2:**
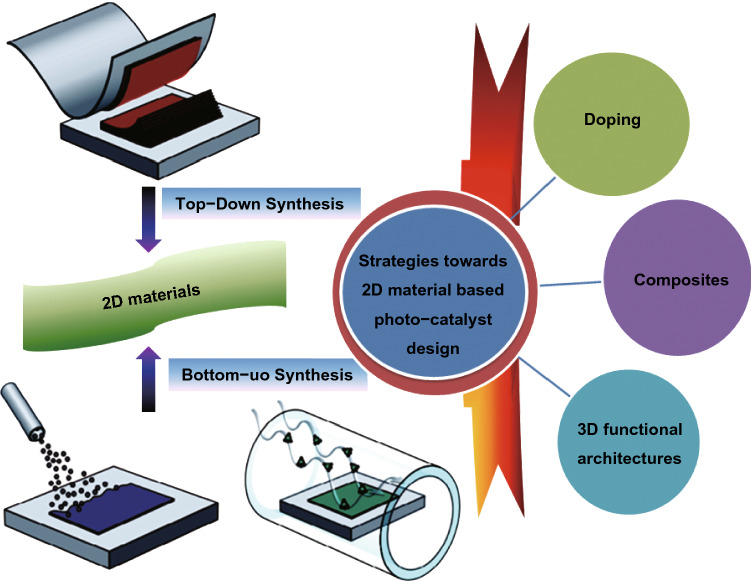
Scheme of 2DMs synthesis and strategies toward 2DM-based photo-catalyst design

The catalysts’ nano-structure is another supreme feature to be considered as it can be used to analytically study and compare diverse catalysts to realize tendency in activity. Generally, the size of photo-catalyst materials also affects their electronic arrangement originated through quantum confinement effects (less than 10 nm) and degree of interface with support, as smaller sized photo-catalyst materials have a larger portion of atoms at metal support edge [57]. For example, Taejong Paik et al. [80] defined the optical BG increased in tungsten oxide (WO_*x*_) NWs compared to stoichiometric WO_3_ bulk counterpart, because of Burstein–Moss shift. This increment confirmed direct photo-catalytic H_2_ evolution from WO_*x*_ NWs via alcohol photo-reform. The stable H_2_ production on platinized WO_*x*_ NWs is pragmatic under conditions where platinized bulk WO_3_ and bulk WO_2.9_ powders either do not show activity or show very low rates, proposing that enhanced surface area is the answer for enhanced activity. As a result, controlled size and composition can cause unanticipated and important alterations in SC photo-catalytic materials properties [81]. As an ideal candidate for photo-catalysis, the mainly studied 2DMs-based photo-catalysts can be divided into different types: counting, MOs, metal composite oxides, MHOs, bismuth-based materials, metal chalcogenides, and metal-free photo-catalysts. Based on photo-catalyst compositions, the 2DMs used in photo-catalysts can be mainly categorized as illustrated in Fig. 2. The 2DMs can be synthesized either through exfoliation of parent layer material through top-down method or formed from small molecules using bottom-up self-assembly technique. Synthesis of 2DMs with tunable layer number, edge morphology, and degree of crystallinity is vital for utilizing these materials for elevated activity catalytic applications and is also discussed in our recently published reviews [3, 4, 19]. Therefore, in this part, we will only provide a concise introduction for basic properties with small explanations about the synthesis strategies of such three types of 2DMs, which are utilized for photo-catalysis applications.

### 2D metal Oxides (MOs) NSs

**Fig. 3:**
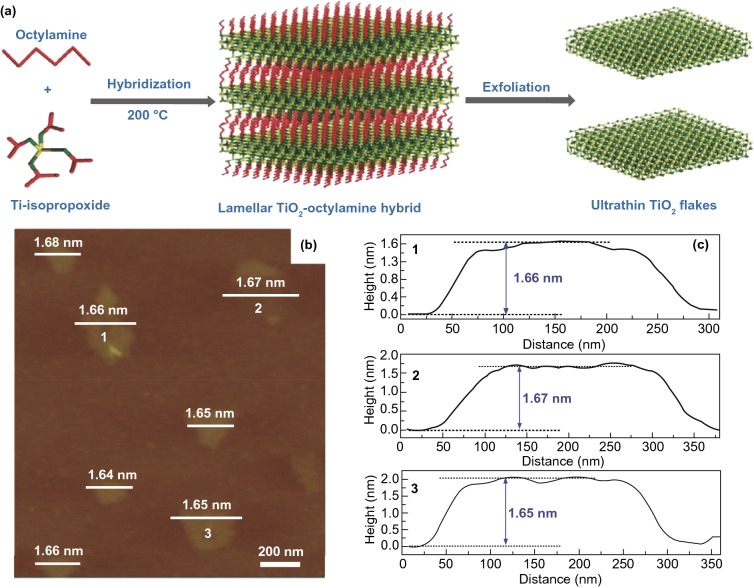
The ultra-thin TiO_2_ flakes. **a** Synthesis scheme. Adapted with permission from Ref. [82]; **b** AFM image, **c** height profiles correspond to AFM image in **b**. Adapted with permission from Ref. [32]

The MOs are broadly studied for photo-catalysts. Certainly, fabricating 2DMs-based MOs is considered as an efficient way to maximize SSA and charge migration and hence gets a competent photo-catalytic performance [32]. So far, numerous MOs with 2DMs structures have been formed and used in photo-catalysis applications, for example TiO_2_, Fe_2_O_3_, Cu_2_O, ZnO, WO_3_, SnO, In_2_O_3_, CeO_2_, HNb_3_O_8_, etc. [82]. Due to the basic non-layered structure feature, some 2D-MOs were complex to be formed by facile ultrasonic exfoliation technique from their bulk counterparts. So, numerous other means were applied for controlled formation of 2D-MOs. For example, a lamellar inorganic–organic hybrid intermediate policy was planned to form ultra-thin TiO_2_ NSs [82]. Utilizing Ti-isopropoxide as a Ti source, octylamine as a capping reagent, 2-phenyl ethanol as solvent, lamellar TiO_2_-octylamine hybrid precursors were obtained via solvothermal process (Fig. 3) [32]. The ultrasound-based exfoliation-resulted powder was washed to eliminate octylamine and get clean, ultra-thin TiO_2_ NSs. The AFM result showed that TiO_2_ NSs’ average thickness was about 1.66 nm. A lot of other types of MOs-NSs, such as Cu_2_O [83] and In_2_O_3_ [84], were also formed via a similar technique. Taking advantage of ultra-thin size, the enhanced density of states (DOSs) by Fermi level (FL) and enhanced charge density on TiO_2_ NSs surface were obtained. For this reason, TiO_2_ NSs displayed quick transport of carriers and therefore achieved 450 times improved photo-catalytic activity as compared to bulk TiO_2_ for CO_2_-reduction for formate fabrication. Additionally, exfoliated single-crystalline WO_3_ NSs were formed by Bi_2_W_2_O_9_. On account of layered Bi_2_W_2_O_9_ structure that is composed of [W_2_O_7_]^2−^ and [Bi_2_O_2_]^2+^ layers, the WO_3_ layers were attained through careful etching of [Bi_2_O_2_]^2+^ layers via processing of acids like HCL and the stabilized WO_3_ layers can be obtained through the tetrabutylammonium hydroxide surfactant. These exfoliated WO_3_ NSs showed an improved BG as compared with bulk-WO_3_, caused by quantum confinement effect. With exception of an exfoliation method, the direct preparation development of MOs-NSs was obtained by wet chemical technique. Utilizing surfactant’ self-assembly approach through polyethylene oxide–polypropylene oxide–polyethylene oxide and ethylene glycol as co-surfactant, various MOs with ultra-thin thickness were formed, for instance TiO_2_, Fe_3_O_4_, Co_3_O_4_, ZnO, MnO_2_, and WO_3_ [32].

**Fig. 4:**
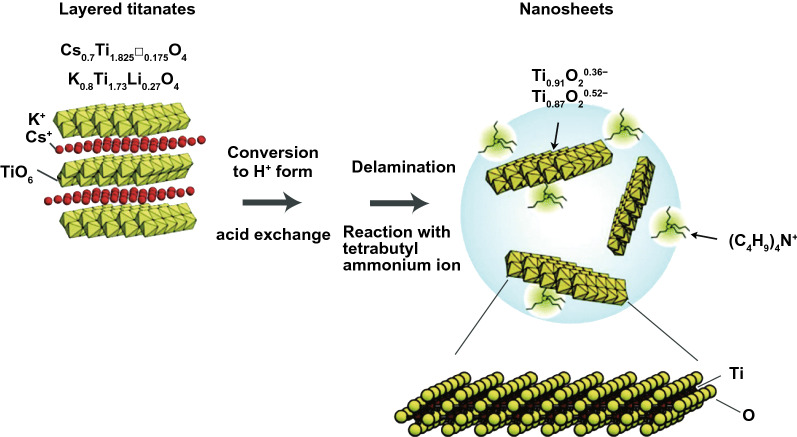
Scheme showing the crystal structure of lepidocrocite kind titanate and its exfoliation into TiO_2_ NSs. Adapted with permission from Ref. [29]

In the last four decades, various MOs, e.g., TiO_2_, ZnO, SnO_2_, WO_3_, and Fe_2_O_3_, were broadly examined as photo-catalysts [42, 85]. Among them, TiO_2_ was the most explored one due to its good stability, biocompatibility, and favorable electronic structure as well as light absorption nature [34]. The 2D-TiO_2_ NSs obtained from the exfoliation of layered titanate have drawn attention in utilizing them as photo-catalysts [86]. The 2D-TiO_2_ NSs showed SC nature similar to their bulk cousins and include rutile and anatase form of TiO_2_, but with somehow superior BG because of the size quantization. For instance, Ti_0.91_O_20.36_-NSs exhibited a BG of ~ 3.8 eV that was higher than that for anatase TiO_2_ (3.2 eV) [87]. Top-down multi-step access found on intercalation and exfoliation of layered MOs was well recognized to form MO-NSs [88]. For example, for TiO_2_-NSs, layered titanates were initially formed by high temperature, conventional solid-state reaction of TiO_2_, and mixture of alkali metal carbonates (Fig. 4).

After that, it was developed with an acid solution to generate protonated intermediate by ion-exchange route. The interlayers of protonated titanate were more extended by changing protons with a definite quantity of bulky organic ions, such as tetrabutylammonium cations (TBA^+^). In suitable condition, layered configuration was exfoliated induced via weak shear force, for instance, mechanical shaking in aqueous solution. Different kinds of layered MOs-NSs, such as WO_3_, titanoniobate [89] (TiNbO_5_, Ti_2_NbO_7_, and Ti_5_NbO_14_), perovskite oxides [90] (K_2_Ln_2_Ti_3_O_10_, together with KLnNb_2_O_7_ and RbL_n_Ta_2_O_7_ (here L_n_ stands for lanthanide ions)), HNb_3_O_8_, HCa_2−*x*_SrxNb_3_O_10_, and HCa_2_Nb_3−*y*_TayO_10_ [91], were formed through analogous solid-state reactions method and wet-chemical exfoliation methods. For instance, titanoniobate NSs have exhibited enhanced photo-catalytic performance in organic pollutant removal [92]. Recently, Tae et al. studied the formation of numerous diamond-shaped titanate NSs with a normal lateral size < 30 nm, by applying a straightforward wet-chemical technique [93]. In very recent times, Zhou et al. formed freestanding, SL Bi_2_WO_6_ MSs by wet-chemical technique via using cetyltrimethylammonium bromide. Bi-atoms on SL were not saturated; hence, introduced numerous active sites on surfaces, which generated h^+^s directly under light irradiation. An excellent photo-catalytic performance of SL Bi_2_WO_6_ for photo-degradation of RhB was recognized by fast charge carrier separation at highly photo-active surface [94].

### Metal Composite Oxides

Compared to MOs, metal composite oxides also showed advantages to photo-catalysis, and numerous metal composite oxides were formed with ultra-thin thickness [91]. Consistent with acid/base effect and ion intercalation supported exfoliation method, HNbWO_6_ NSs were obtained by dispersing the layer HNbWO_6·1_·5H_2_O into tri-ethanolamine aqueous solution [95]. The results based on AFM calculations showed that HNbWO_6_ NSs thickness was about 1.8 and 2.0 nm, which are in agreement with SL significance. As-synthesized HNbWO_6_ NSs suspensions displayed a proficient activity for photo-catalytic H_2_-evolution with a moderate rate of 158.9 µmol h^−1^. Furthermore, ion-exchange approaches through utilizing ultra-thin precursor were used for synthesis of metal composite oxides. For instance, SnNb_2_O_6_ NSs were obtained through K_4_Nb_6_O_17_ NSs and SnCl_2_ as precursors [96]. Through K_4_Nb_6_O_17_ ultra-thin thickness, it was preserved in SnNb_2_O_6_ with ~ 3 nm thickness, as confirmed through AFM study. In comparison with bulk SnNb_2_O_6_, the SnNb_2_O_6_ NSs were having improved BG and more negative CB potential, denoting good reduction capability for photo-catalytic-based H_2_-evolution. Furthermore, charge transfer effectiveness in SnNb_2_O_6_ NSs was also enhanced because of ultra-thin thickness. Additional research showed that the outstanding vis-light H_2_-evolution activity was acquired over SnNb_2_O_6_ NSs, approximately 14 times superior to bulk SnNb_2_O_6_.

### Metal Hydroxides (MHOs)

Ultra-thin MHOs were increasingly considered as significant class in 2DMs, which showed an exciting view in numerous sectors, for example catalysis, energy storage, and conversion. On account of simplicity of guideline for cations, the preferred BG was formed in MHOs by incorporating particular photo-active metal cations. So, the ultra-thin 2D-MHOs structure showed a great potential toward photo-catalytic uses. For example, ZnAl-layered double hydroxide (LDH) 2D-NSs were formed via a reverse micelle technique and used as photo-catalyst for converting CO_2_ to CO [97]. By means of sodium dodecyl sulfate as surfactant, 1-butanol as co-surfactant, translucent and stable reverse emulsion structure was created in an iso-octane/H_2_O mixed solution. Following Al and Zn sources addition to mix solution, urea was used to generate alkaline condition and formed ZnAl-LDH with ultra-thin configuration. Thickness was about 2.7 nm in standing NSs in TEM image and is equivalent to the thickness of 2D-LDHs’ layers. Due to the ultra-thin thickness, O_2_ vacancies (V_o_) were formed in ultra-thin ZnAl-LDH NSs, resulting in the creation of Zn^+^–V_o_ complexes. The DFT-based study showed a novel defect-level hybridization with both occupied Zn 4*s* orbitals and O_2*p*_ orbitals emerging in BG of ultra-thin ZnAl-LDH NSs compared with bulk ZnAl-LDH. The Zn^+^–V_o_ complexes can provide e^−^ trap sites for CO_2_ photo-reduction. Consequently, an appreciably amplified photo-catalytic activity for CO_2_-reduction was obtained for ultra-thin ZnAl-LDH NSs compared with bulk ZnAl-LDH. Except ZnAl-LDH, several new MHOs with ultra-thin thickness, for example CoOOH [98], NiTi-LDH, and ZnTi LDH [99], also showed wonderful performance for diverse photo-catalytic uses.

### Metal Chalcogenides

**Fig. 5:**
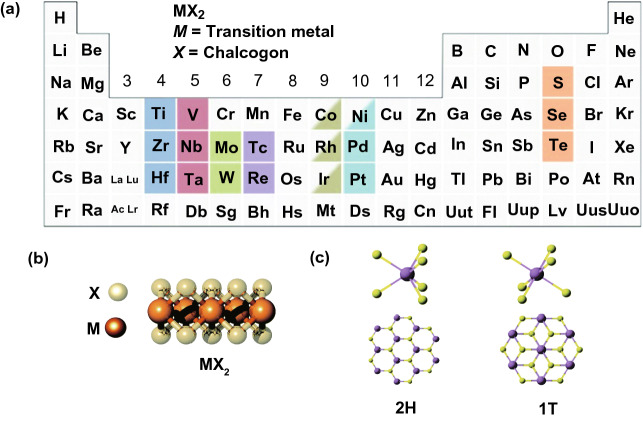
**a** Transition metals and three chalcogen elements (yellow color) which are composed of TMD layer structures. **b** TMD structures. **c** MoS_2_ two-phase (1T, 2H) structures. Adapted with permission from Ref. [100]

The TMDs have gained much interest because their mechanical, optical, and electrical characteristics were explored for a wide range of applications, for instance biosensors, catalysis, lithium battery cathodes, transistors, memory devices, photovoltaics, photodetectors, photo-catalytic solid lubricants, and PEC conversions. The TMDs (e.g., MoS_2_, WS_2_, and TiSe_2_) are a large group of layered materials with common representation as “MX_2_,” where M is a transition metal element of group 4–10 ((Ti, Hf, Zr), (Ta, V, Nb), and (W, Mo)) and X is the chalcogen atom (S, Se, Te). The TMD NSs have different functions in PC and PEC applications. They behave as a photo-sensitizer via increasing light harvest ability in vis region of sun irradiation, a charge separator throughout appropriate energy band arrangement, and a charge carrier. Correct function of 2D nanosheets (NSs) depends on utilization of reaction scheme. In consequence of special electronic configuration, in general metal chalcogenides showed a comparatively broad light absorption area (Fig. 5), which were measured to be a group of talented materials having photo-catalytic uses. Normally, the stoichiometry of TMDs can be expressed through formula MX_2_, in which M and X signify a chalcogen and a transition metal, respectively. The single layer of a TMD involves three atoms, where M is situated in two X (Fig. 5a, b). The configurations of 1T and 2H phases of MoS_2_ are shown in Fig. 5c.

Recently, many 2D metal chalcogenides have been formed and showed an excellent photo-catalytic performance, e.g., CdS, MoS_2_, SnS_2_, SnS, In_2_S_3_, ZnIn_2_S_4_, ZnSe, and SnSe [101]. The synthetic techniques of such 2D metal chalcogenides generally concentrate on exfoliation, because of essential layer structures. Taking hexagonal SnS_2_ (h-SnS_2_), such as h-SnS_2_ SLs, can be attained through refluxing bulk-SnS_2_ in formamide to rupture interlayer vdWs’ interactions [102]. Almost transparent aspect of SnS_2_ NSs in TEM image exposes ultra-thin thickness, and it was described to be 0.61 nm via AFM, well matched with SL-SnS_2_ slab along [001] direction. As a result of SL configuration, electronic structure of SLs-SnS_2_ experienced discrete changes, with increased BG, higher DOSs at VB edge, and faster interfacial charge transfer. Therefore, SLs-SnS_2_ delivers a surprisingly improved photo-catalytic H_2_O splitting activity (70 times development) compared to bulk-SnS_2_, under vis-light. The 2D metal chalcogenide NSs, for example MoS_2_ [103], SnS_2_ [104], TiS_2_ [105], WS_2_ [106], MoSe_2_ [107], WSe_2_ [103], etc., are rising as a new significant class of 2DMs in the photo-catalysis applications because of their good electronic properties [108]. Taking MoS_2_ as an example, bulk-MoS_2_ materials have indirect BG of 1.2 eV that is not suitable for photo-catalytic reactions caused by the lack of oxidation or reduction potential (*E*_0_) for activating photo-catalytic method. However, MoS_2_-NSs had been established with having a direct BG of ~ 1.96 eV because of quantum confinement effect that provides MoS_2_/-NSs along appropriate band positions and capability for vis-light absorption. In contrast to most of the layer MOs, vdWs bonding of metal chalcogenide interlayers creates exfoliation of these layers easily. Until now, a lot of top-down approaches are described for the formation of SL or FL metal chalcogenide NSs, for example lithium intercalation–exfoliation, mechanical exfoliation, and liquid phase ultrasonic exfoliation [109]. Furthermore, bottom-up chemical production and chemical vapor deposition (CVD) techniques suggested potential influential alternatives such as exfoliation techniques for fabricating metal chalcogenide NSs. For example, Cheon et al. introduced disk-shaped ZrS_2_ NSs with < 2 nm thicknesses and lateral size ranging from 20 to 60 nm via reacting ZrCl_4_ and CS_2_ in oleylamine [110]. This method was, soon after that, extended for other transition metal selenide and sulfide NSs. The MoS_2_-NSs were formed via solvothermal techniques using (NH_4_)_6_Mo_7_O_24_·4H_2_O and thiourea as precursors [111].

### Bismuth-Based Materials

**Fig. 6:**
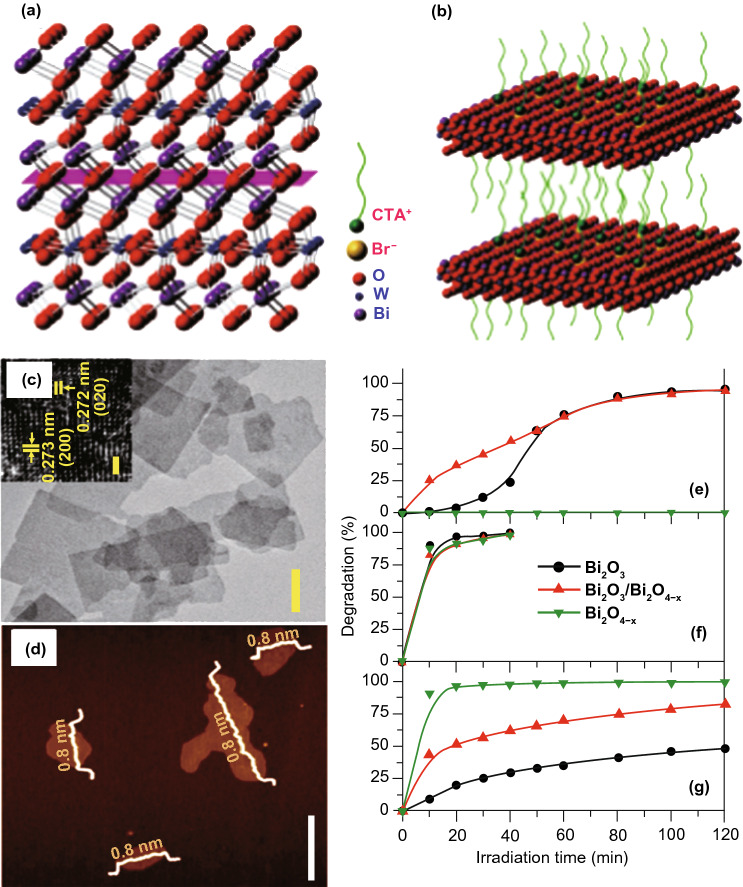
**a** Bi_2_WO_6_ crystal structure. **b** Fabrication method of the SL Bi_2_WO_6_ with CTAB support. **c** TEM/HR-TEM of Bi_2_WO_6_ formed by CTAB support. **d** AFM analysis of SL Bi_2_WO_6_ based on CTAB. Adapted with permission from Ref. [32]. Comparison of photo-catalytic degradation of **e** MB, **f** MO, and **g** phenol over Bi_2_O_3_, Bi_2_O_3_/Bi_2_O_4−*x*_, and Bi_2_O_4−*x*_. Adapted with permission from Ref. [118]

Recently, bismuth (Bi)-based materials have been broadly investigated and studied for their photo-catalysis applications because Bi6s in Bi(III) can hybridize with O_2*p*_ orbitals to generate novel favorable hybridized VB and BG of Bi-based materials which are narrowed for absorption of vis-light. Due to continuous improvement in photo-catalytic performance, numerous Bi-based materials with controlled ultra-thin thickness are formed, for example Bi_2_WO_6_ [112, 113], Bi_2_MoO_6_ [85], BiVO_4_ [114], Bi_2_SiO_5_ [115] (BiO)_2_CO_3_ [94], Bi_3_NbO_7_ [116], BiOX (X = Cl, Br, I) [117], and Bi_2_O_3_/Bi_2_O_4−*x*_ nano-composite [118]. The SL Bi_2_WO_6_ NSs were formed via surfactant cetyltrimethylammonium bromide (CTAB)-supported hydrothermal technique [94], where Br-ions from CTAB were adsorbed at SL Bi_2_WO_6_ surface and produced Coulomb repulsion forces, which delayed stacking of SLs Bi_2_WO_6_. Furthermore, hydrophobic long-chain cationic CTA^+^ at Bi_2_WO_6_ surface supplied an extra surface repulsion to further stop crystal growth along the c-axis. Therefore, SL Bi_2_WO_6_ slab (0.8 nm thickness) with [BiO]^+^–[WO_4_]^2−^–[BiO]^+^ sandwich substructure was achieved, as supported from AFM analysis (Fig. 6a–d). Plentiful coordinative unsaturated Bi-atoms were exposed at SL Bi_2_WO_6_ SNs and act as active sites. After irradiation with light, h^+^s is produced in [BiO]^+^ as e^−^s in [WO_4_]^2−^. Resembling hetero-junction interface, sandwich [BiO]^+^–[WO_4_]^2−^–[BiO]^+^ substructure permits efficient interface for space charge separation. Therefore, SL Bi_2_WO_6_ displayed significantly improved photo-catalytic activity toward pollution deduction in vis-light.

In addition, as-synthesized SL Bi_2_WO_6_ illustrated H_2_-evolution activity under vis-light, even if Bi_2_WO_6_ nano-crystal in fact holds no H_2_-evolution activity. Altering the surfactant type to polyvinylpyrrolidone (PVP), ultra-thin BiOCl NSs were attained by means of a solvothermal treatment [119]. The BiOCl nanoplate’s thickness (≈ 30 nm) was reduced to 2.7 nm, while PVP was applied as a capping agent, as verified through the AFM analysis. The polyvinyl skeleton structure of PVP prevented more development of the BiOCl nano-crystal by generating passivation layer about BiOCl cores via strong interaction with Bi^3+^, N, and O atoms of pyrrolidone ring. This suppressed the agglomeration of BiOCl nano-crystal along c-axis during repulsion forces in between polyvinyl groups. Therefore, ultra-thin BiOCl NSs were prepared via the PVP-assisted solvothermal treatment and this method was further used for synthesis of ultra-thin BiOBr and BiOI 2D form [120]. The attained ultra-thin thicknesses give BiOCl NSs with upshifted CB and VB potentials and reduced the BG relative to BiOCl nano-plates. As such, effective division of photo-induced (e^−^–h^+^)-pairs was obtained and caused an increase in photo-catalytic activity for pollutant removal [32]. Paolo Fornasiero and co-workers also explored Bi_2_O_3_/Bi_2_O_4−*x*_ composite that functions as a potential photo-catalyst (Fig. 6e–g). The aim of the study was to begin active species on photo-catalyst surface via utilizing a non-traditional advancement. Therefore, they utilized (UV–Vis)-light to stimulate alterations in Bi_2_O_3_ surface that produces Bi_2_O_3_/Bi_2_O_4−*x*_ nano-composite arrangement. So, for methylene blue (MB) such surface modifications bring significant enhancement in photo-catalytic performance. The wide BG with respect to Bi_2_O_3_ along excitation considerations proposes that analogous photo-induced crystal modifications, although exist, should be insignificant for TiO_2_-based materials. Until now, only careful designed thermal treatments were able to make exciting anatase/rutile nano-composites with outstanding photo-catalytic performance [118].

### Metal-Free NSs

Excluding metal containing SC 2DMs, metal-free 2DMs were also formed as photo-catalysts. Recently, new classes of metal-free 2DMs have been introduced from lightweight and abundant elements, such as carbon, phosphorus, and binary carbon nitride, boron carbide, and hexagonal boron nitride (h-BN) that reveal new prospects for photochemistry. The 2D-G with hexagonal *sp*^2^-hybridized structure is inspiring great research concern in a range of energy-related uses because of its elevated carrier mobility. The high flexibility and larger SSA alone from accessibility of solution processable graphene oxide (GO) allows 2D-G NSs to simply merge with other SCs to form electronic bridges [121]. Recently, 2D-G have been intensively studied in photo-catalytic fields and demonstrated as competent e^−^-acceptor to improve the charge transfer and reduce (e^−^–h^+^)-pair’s recombination to improve photo-catalytic activity of composite photo-catalysts [3, 4, 18, 19]. More interestingly, although SL 2D-G is recognized as a semimetal with a zero BG, which is not suitable in light absorption, numerous scientists have confirmed that functionalized 2D-G base analogy like 2D-GO could be promising materials for nonmetal photo-catalysts as band structure of GO is associated with its degree of oxidation that can be engineered via choosing appropriate preparation methods. For example, Yeh et al. observed a 2D-GO that could work as active photo-catalyst in H_2_O splitting [122] and can gradually produce H_2_ from 20 vol% methanol solution in H_2_O and pristine H_2_O after irradiation with UV/Vis-light. After that, Hsu et al. studied 2D-GO and showed an elevated photo-catalytic efficiency for transformation of CO_2_ to methanol (CH_3_OH) by solar light irradiation [123]. As an equivalent of 2D-G, g-C_3_N_4_ NSs were rapidly rising because of their excellent chemical and electronic properties [124]. Bulk g-C_3_N_4_ has a layered 2D configuration and proper BG (~ 2.7 eV) for light absorption in visible range. The g-C_3_N_4_ NSs were obtained through delaminating bulk layered g-C_3_N_4_ that is usually formed via pyrolysis of N_2_-rich precursors through bulk reaction or polycondensation.

**Fig. 7:**
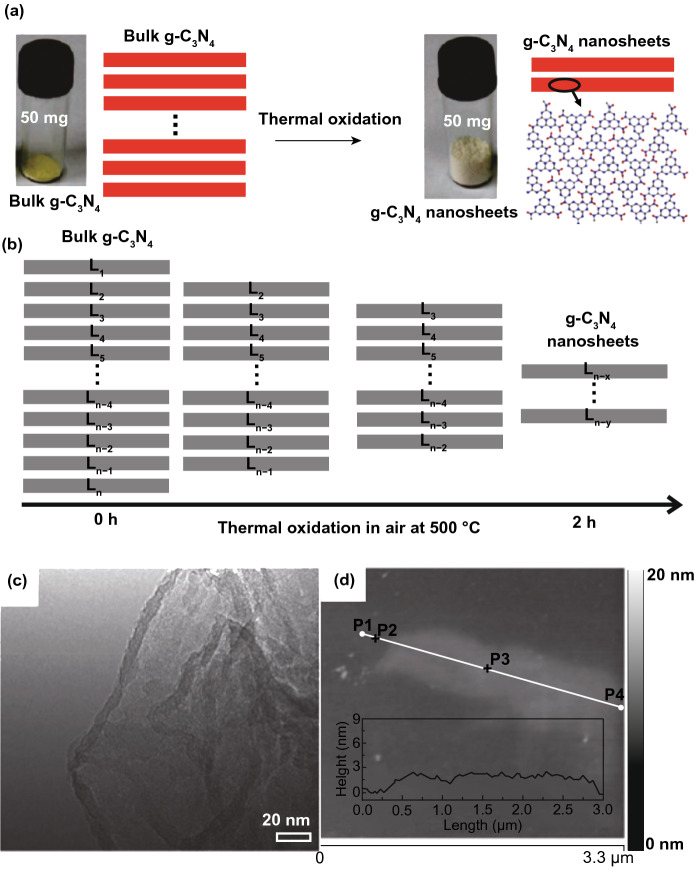
**a** Scheme shows bulk g-C_3_N_4_ and g-C_3_N_4_ NS structures. Adapted with permission from Ref. [29]. **b** Scheme to fabricate g-C_3_N_4_-NSs through thermal oxidation etching of bulk g-C_3_N_4_ at 500 °C in air. **c** TEM image, **d** AFM analysis of g-C_3_N_4_-NSs. Adapted with permission from Ref. [32]

A new metal-free photo-catalyst, with outstanding photo-catalytic proficiency of g-C_3_N_4_ NSs under vis-light irradiation, was verified in many photo-catalytic uses. For example, Niu et al. [125] studied a simple top-down approach to form g-C_3_N_4_ NSs via oxidation etching of bulk g-C_3_N_4_ in air under high temperature (Fig. 7). The acquired g-C_3_N_4_ NSs thickness was about 2 nm with SSA 306 m^2^ g^−1^, which was high in comparison with bulk phase. Quantum confinement effect causes enhanced e^−^-transfer ability toward in-plane direction, and the lifetime of photo-generated charge carriers was improved. Therefore, photo-catalytic performance of g-C_3_N_4_ NSs for H_2_-production process was really enhanced. In recent times, other liquid phase exfoliation techniques are formed to synthesize g-C_3_N_4_ NSs from bulk counterpart. For instance, Yang et al. prepared freestanding g-C_3_N_4_ NSs through liquid phase exfoliation of g-C_3_N_4_ powder in isopropanol; this exhibited good photo-catalytic effectiveness for H_2_-evolution by applying vis-light irradiation. Photo-catalytic efficiency of exfoliated NSs was higher > 17 factor contrast to non-exfoliated counterpart and with a factor of > 8 than already described g-C_3_N_4_ NSs [126]. Apart from distinctive energy band configuration, g-C_3_N_4_ is more active toward many photo-catalytic uses, for example H_2_-evolution, CO_2_-reduction, pollutant deduction, disinfection, etc. As a result of in-plane graphite-like layer configuration with strong C–N covalent bonding and interlayer weak vdWs’ forces, bulk g-C_3_N_4_ was accountable to be exfoliated and obtained in FL or even SL form. Generally, there are two methods for g-C_3_N_4_ exfoliation, i.e., thermal oxidation and ultra-sonication-based liquid exfoliation techniques. In view of that, H_2_-bond coherent strands of polymeric melon units in layers were not sufficiently stable beside oxidation. Liu et al. [125] formed a thermal oxidation exfoliation method to form an ultra-thin g-C_3_N_4_ NSs. Thicknesses of bulk g-C_3_N_4_ were steadily reduced with increasing times through layer-by-layer etching method (Fig. 7b–d). After 120 min, thermal oxidated g-C_3_N_4_ NSs with almost 2 nm thicknesses were obtained. Since quantum confinement effect and increased BG promoted e^−^s migration rates along in-plane direction, H_2_-evolution activity improved 5.4 times, as compared to bulk counterpart. Encouraged by this synthesis method, numerous advance researches achieved organizing ultra-thin g-C_3_N_4_-NSs via modified techniques [127, 128]. The ultra-sonication assisted liquid exfoliation was observed as another effective technique to attain ultra-thin g-C_3_N_4_ NSs because of fundamentally layered structure.

In exfoliation process, the efficiency is affected due to surface energies, and when solvent and bulk materials match each other, exfoliation was extremely good. The calculated g-C_3_N_4_ surface energy was almost 115 mJ m^−2^, in a good agreement with H_2_O (~ 10^2^ mJ m^−2^). Thus, with the use of H_2_O in g-C_3_N_4_ liquid exfoliation, exfoliated NSs with almost 2.5 nm thickness were acquired [129]. Based on the analogous law, Ajayan and co-workers [130] calculated g-C_3_N_4_ exfoliated by isopropanol. The as-formed g-C_3_N_4_-NSs were having homogeneous thickness of ~ 2 nm. This ultra-thin thickness of g-C_3_N_4_-NSs displayed an improved BG as well as charge migration rate in contrast to bulk g-C_3_N_4_. This caused a 9.3 times higher photo-catalytic-based H_2_-evolution. Similarly, other solvents were also searched to form ultra-thin g-C_3_N_4_ and obtained improved photo-catalytic performance [131]. Despite g-C_3_N_4_, other metal-free materials were also formed, which act as photo-catalysts for different uses [132]. For example, ultra-thin silicon NSs controlled synthesis via molten salt-induced exfoliation and chemical reduction of natural clay [133]. Ultra-thin silicon NSs (≈ 5 nm thickness) showed an excellent H_2_-evolution performance from a H_2_O methanol mixture. Advanced investigations showed that proficient H_2_O splitting was obtained over ultra-thin silicon NSs lacking addition of co-catalyst or sacrificial agent [134]. Unfortunately, ultra-thin silicon NSs have experienced serious activity decline by extended time. How to approve suitable approach to increase stability might be heart of H_2_O splitting over ultra-thin silicon NSs [32].

### Other Metal Containing 2D-NSs

In addition to aforementioned different types of materials, other recently introduced 2DMs, for example layered metal oxy-nitride and oxy-halides, and metal carbides, also have a great potential for photo-catalysis uses after chemical doping or combining with other SCs. For instance, bismuth oxy-halides (BiO_X_, X = Cl, Br, and I) were gotten increasing interest because of their outstanding photo-catalytic nature, that are analogous to or even greater than those of the anatase TiO_2_ [119]. Moreover, neutral layers of the Ti_3_C_2_(OH)_2_ formed through HF-assisted exfoliation of metal carbides, for example Ti_3_AlC_2_, were verified as competent photo-catalyst for adsorption and photo-catalytic decomposition of organic molecules in an aqueous atmosphere [135].

### 2D/2D Hetero-structures

Properly developing the 2D/2D hetero-structures confirmed the most talented form for further boosting the photo-catalytic activity, because of that hetero-junction interfacial effect [136]. The hetero-junction interfacial effect can encourage separation and therefore extend lifetime of the photo-generated (e^−^–h^+^)-pairs in catalyst that directly or indirectly contributes to redox reaction of photo-catalytic H_2_-production or organics degradation. Several attempts were applied to engineer 2D-component or reinforce the interfacial acting force to form the capable 2D/2D photo-catalysts. Although hetero-junction found on a range of dimensions (e.g., 2D/2D, 3D/3D, and 2D/3D) with exposed interface put right contact, they are all possible efficient catalysts. The 2D/2D hetero-junctions have different advantages for catalysis, as follows:

1. High catalytic active sites because of great SSA/interface area and ultra-thin thickness.
2. Charges are easily transferred because of small basic resistance and short transport path in 1D of the ultra-thin 2D components.
3. Transparency consequence from ultra-thin thickness is helpful in light absorption.

Therefore, plan as well as the use of 2D/2D layered hetero-structures has rapidly become the most up-to-date research topics. Recently, the family of ultra-thin, 2D layered materials formed significantly. Further, other than presently used ultra-thin 2D-NSs, for example 2D-G, TMDCs, and noble metals, there are many other 2D-NSs with changeable electronic and physical properties formed from the last few years, e.g., MOs, h-BN, black phosphorus, metal–organic frameworks (MOFs), organic crystals, and covalent organic frameworks (COFs) [136].

## Electronic Structure Engineering

Engineering an electronic configuration, the properties of ultra-thin 2DMs are experiencing a different variation, offering probabilities to enhance or even provoke novel photo-catalytic activity. There are a range of ways to engineer the electronic structure of ultra-thin 2DMs, e.g., thickness tuning, component tuning, defect engineering, doping, and so on. Now, we will explain most of them in detail in the next section.

### Component Tuning

**Fig. 8:**
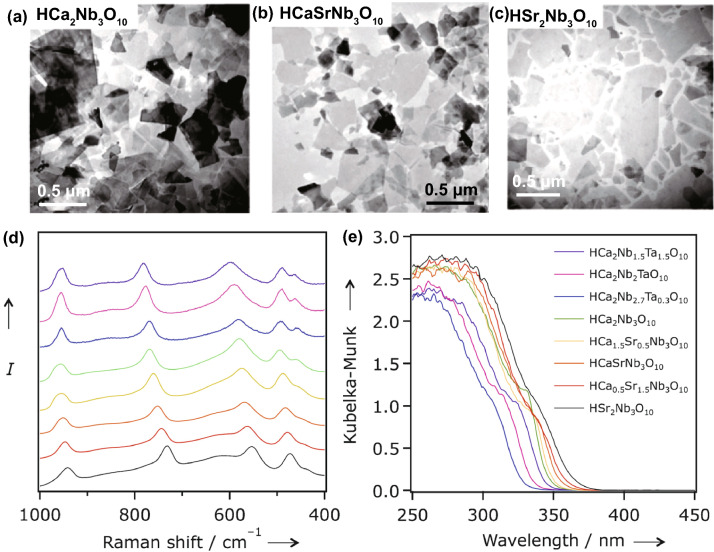
**a**–**c** TEM results of TBA^+^-exfoliated Ca_2−*x*_Sr_*x*_Nb_3_O_10_-NSs. **d**, **e** Raman spectra and UV–Vis diffuse reflectance spectrum of restacked HCa_2−*x*_Sr_*x*_Nb_3_O_10_ and HCa_2_Nb_3−*y*_Ta_*y*_O_10_ NSs. Adapted with permission from Ref. [137]

For photo-catalytic employment, component of SC establishes band configuration. As reactivity of the photo-generated e^−^s and h^+^s toward resultant surface redox reactions was usually defined by band edge potentials, component of SC demonstrates a vast effect on photo-catalytic performances. Particularly, for ultra-thin 2DMs, the electronic configuration strongly relies on equivalent constituent. Maeda et al. [91] formed HCa_2−*x*_Sr_*x*_Nb_3_O_10_ and HCa_2_Nb_3−*y*_TayO_10_ NSs with restricted energy band structure via interlayer exchange K^+^-ions with protons in layered KCa_2−*x*_Sr_*x*_Nb_3_O_10_ as well as KCa_2_Nb_3−*y*_Ta_*y*_O_10_ and after that more undergo exfoliation. By gradually engineering atomic component in NSs, the optical absorption of materials is deeply suffered (Fig. 8).

Thoroughly, commencement of absorption edge in HCa_2−*x*_Sr_*x*_Nb_3_O_10_ showed a clear redshift with improved Sr-content, accompanying via BG energy reduced from 3.59 (*x* = 0) to 3.40 eV (*x* = 2). Additionally, onset of blueshift absorption edge was determined for KCa_2_Nb_3−*y*_Ta_*y*_O_10_ with higher Ta contents. The substitution of Nb^5+^ by Ta^5+^-ions caused more negative CB potential, which was liable for blueshift of the onset absorption edge. Thus, tunable light absorption performance was obtained by altering component and further showed an important effect on the photo-catalytic H_2_-evolution reaction. By utilizing an analogous plan, energy band structure of ternary sulfides, H_2*x*_Zn_1−*x*_In_2_S_4_, was efficiently engineered with ZnIn_2_S_4_ and showed optimal photo-catalytic H_2_-evolution rate [138]. Another significant case was tuning of halogen/O_2_ ratio in the bismuth oxy-halide to tune electronic structures. In accordance with DFT calculations, VB top of bi-oxy-halide was mainly composed of O_2*p*_ and X*np* (*n* = 3, 4, and 5 for X = Cl, Br, and I) hybrid orbitals, while CB was primarily comprised of Bi6*p* orbitals. In the course of engineering O, X elements, BG and band edge potentials were efficiently engineered. Thus far, a number of Bi oxy-halide NSs with tuned O, X component were synthesized, for example Bi_12_O_17_C_l2_ [139], Bi_4_O_5_Br_2_ [140], Bi_4_O_5_I_2_ [141], and so on. In general, with decreasing Br or Cl content, acquired materials showed narrowed BG than that of resultant BiOCl or BiOBr and reduction of I content resulted in an increase in BG comparative to BiOI. Benefiting from energy band structure range, tuned bi-oxy-halide ultra-thin NSs are showing superiority toward various photo-catalytic employments.

### Thickness Tuning

SC thickness is an important factor to an electronic structure engineering and photo-catalytic performance optimization. As a result of known quantum confinement effect, BG of SC undergoes an increase, when materials thickness is reduced. Moreover, surface effect is aggravated as thickness reduced to an atomic size. The electronic DOSs can improve at the surface of ultra-thin 2DMs in comparison with interior of bulk materials. Such characteristics showed a significant impact on photo-catalytic efficiency of ultra-thin 2DMs. Based on thermal oxidation etching way, ultra-thin C_3_N_4_ NSs with thickness of ~ 2 nm were formed [125]. As thickness reduced to atomic size, electronic structure of C_3_N_4_ suffers from major difference. Consequently, due to quantum confinement effect, BG increased from 2.77 eV for bulk C_3_N_4_ to 2.97 eV in ultra-thin 2D-NSs. Simultaneously, thickness reduction grants guarantee to improve an electron transport capability toward in-plane direction and enhanced duration of photo-generated charge carriers.

**Fig. 9:**
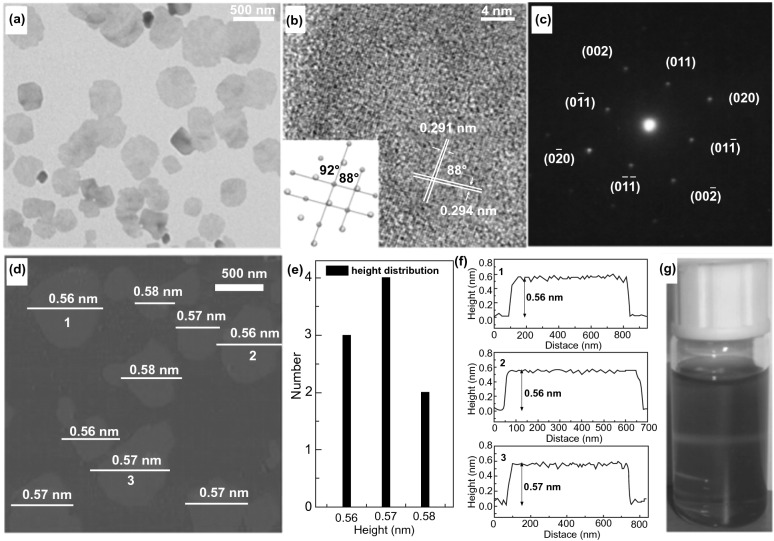
**a**, **b** TEM and HR-TEM, **c** selected-area electron diffraction. **d–f** AFM image, height distribution, and height profiles. **f** (1–3) stands for 1–3 in **d**. **g** Colloidal H_2_O/ethanol (1:1) dispersion of the as-synthesized products showing Tyndall effect. Adapted with permission from Ref. [144]

Additional research originates in which CB edge of ultra-thin C_3_N_4_ NSs showed upshift in comparison with bulk counterpart [142]. So, photo-generated e^−^s holds strong reduction capability and helps in improving the photo-catalytic H_2_-evolution performance. Despite C_3_N_4_, electronic structure tuning is gained within other SCs through thickness engineering. Through thickness reduction of bulk SnNb_2_O_6_ to 50 and 3 nm, resultant BG increased from 2.30 to 2.35 and 2.43 eV, respectively, causing upshifting of CB edge [96]. It is suggested that thickness engineering was an efficient way to alter energy band configuration of SCs. Moreover, when bulk materials thickness is regularly decreased to an atomic size thickness or even SL, the ratio of exposed surface atoms to whole atoms can be prominently improved. Lack of nearby atoms formed plentiful coordination on unsaturated surface atom with dangling bonds and is leaning to bond with other atoms to attain stability. So, these surface atoms displayed a high surface energy and chemical reactivity. The free-standing SnSe and SnS NSs with all exposure surface atoms were acquired through exfoliating their bulk counterparts in mixed solvent of H_2_O and ethanol [143]. Taking SnS as an example, with large-area NSs resembling morphology with lateral size of almost 500 nm, their transparent properties were studied by TEM investigation, showing ultra-thin thickness of as-synthesized material (Fig. 9). The average height of SnSe NSs, calculated by AFM, was about 0.57 nm that agrees with thickness of half unit cell. Hence, these SL-exfoliated NSs calculated band structures which proposed a change from an indirect SC in bulk SnS to direct SC for SnS SLs. In comparison with bulk SnSe, enhanced DOSs were obtained at VB edge of SL SnS, which were enabling SL SnS with improved carrier transport efficiency. To get benefit from SL structure, SLs SnS showed improved photo-absorption and charge separation efficiency and later supported H_2_O splitting performance.

### Doping

The appropriate doping was observed as a competent approach for engineering physicochemical property of 2DMs. Conscious manufacturing of extrinsic metal or nonmetal species into SC lattice presents the prospect to adjust electronic or surface configurations of host material for enhancing photo-catalytic performance. Types and allocation of dopants are very important to control properties of host SCs. Advantages of atomic size of 2DMs; doping perhaps is a very sufficient plan to influence properties of ultra-thin 2DMs. Normally, doping always happens on bulk materials’ shallow surface due to lack of atoms accessing gallery that demonstrated a small manipulation on their total performance. Concerning ultra-thin 2DMs, atomic thickness permits efficient doping of dopants and just needs small diffusion penetration. So, it is enviable to tailor heteroatom into 2DMs and builds high competence methods.

#### Metal Doping

Metal ions incorporation into crystal lattice causes a rise in impurity levels in SCs forbidden band. Onset light absorption edge redshift is frequently examined that is recognized to cause a transition of impurity quantities to CB or VB. Normally, the very capable doped photo-catalysts primarily depend on doping alteration of metal ions that satisfied the criteria, i.e.,

1. The e^−^s and h^+^s can be trapped through dopant and make sure efficient confined separation.
2. The captured e^−^s and h^+^s are generated and transferred on surface successfully.

**Fig. 10:**
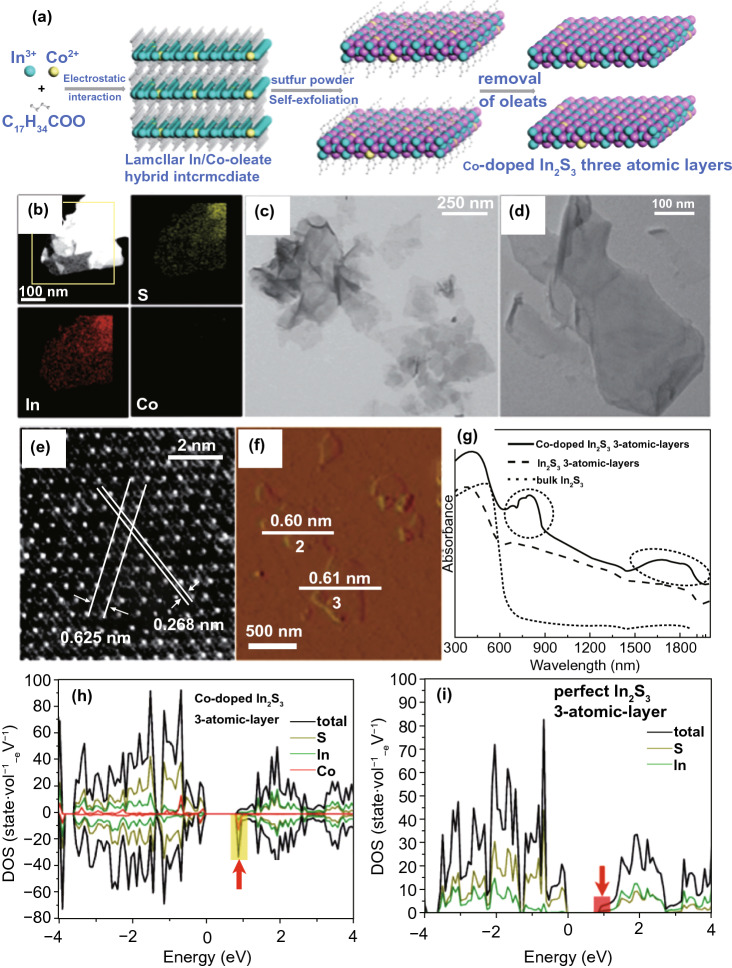
**a** Representation of the formation of Co-doped In_2_S_3_ three atomic layers. **b–g** Characterizations for Co-doped In_2_S_3_ three atomic layers: **b** HAADF-STEM image and EDS mapping of an individual Co-doped In_2_S_3_ three atomic layer, **c–e** TEM images and HR-TEM image, **f**, **g** AFM image, and height profiles. **h** DOSs of Co-doped In_2_S_3_ three atomic layer slabs and **i** ideal In_2_S_3_ three atomic level slab. Adapted with permission from Ref. [147]

Xie and co-authors [145] doped In_2_S_3_ NSs with Co to optimize photo-catalytic H_2_O splitting. Through a lamellar inorganic–organic hybrid intermediate approach, Co-doped In_2_S_3_ NSs (0.59 nm thick) along with 3-atomic layers thickness were formed (Fig. 10). The electronic configuration of In_2_S_3_ using three atomic layers and Co-doped In_2_S_3_ was first verified via DFT simulations. To simulate existence of Co-dopant, certain ultra-thin In_2_S_3_ NSs with noticeably enhanced DOS are developed at conduction band maximum (CBM) as compared with perfect, ultra-thin In_2_S_3_ NSs. Additionally, Co-ion doping provides a Co-doped In_2_S_3_ material with numerous other energy levels that were resultant from Co_3d_ splitting. Under light irradiation, e^−^s was simply excited through *d* → *d* internal transition of the Co-ions in tetrahedral coordination, allowing generation of more photo-generated (e^−^–h^+^)-pairs. These results were confirmed through light absorption difference of In_2_S_3_ NSs and Co-doped In_2_S_3_ in (UV–Vis)-diffusion reflectance spectrum. Considerable advancement in light absorption was viewed from 600 to 2000 nm that was consigned to creation of dopant energy levels of Co. To get advantages from doping of Co, photo-generated charge separation effectiveness enhanced about 25-fold increase in average recovery duration, as practiced through an ultrafast transient absorption spectroscopy (UTAS). Therefore, Co-doping permits 10 times developed photo-catalytic activity for H_2_O splitting compared to perfect In_2_S_3_ NSs. Similarly, other metal elements were also utilized for doping to engineer electronic structure of ultra-thin 2D-hosted photo-catalyst, for instance Pt, Rh, Cr, Fe, Cu, and so on [146]. For example, Fe was doped into ultra-thin BiOCl NSs, which extended light absorption range from UV to Vis-light. Photo-catalytic activity for pollutant removal and H_2_-evolution was increased. These results certainly verified that metal element doping is an efficient way to tune electronic structure of ultra-thin 2D photo-catalysts and can promote photo-catalytic performance.

#### Nonmetal Doping

**Fig. 11:**
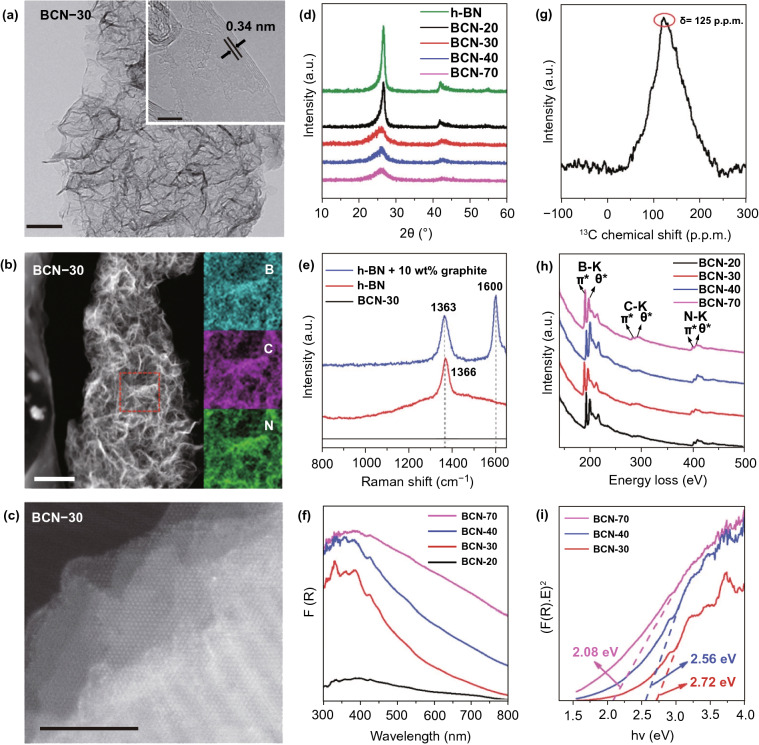
**a** HR-TEM of BCN-30. **b** Dark-field TEM image of BCN-30 and element map. **c** HR-STEM of the BCN-30 sample along (002) facet. **d** XRD of h-BCN. **g** NMR spectra of BCN-30. **e** Raman spectrum of h-BN and BCN-30 and physical mixture of h-BN and graphite. **h** EELS spectrum of BCN-*x*. **f** UV–Vis diffuse reflectance spectra of BCN-*x*. **i** BG calculation of BCN-*x* from the (F(R) E)n versus E plots. Adapted with permission from Ref. [151]

Concerning nonmetal elements doping, two direct theories were suggested to modify an electronic arrangement and hence influence photo-catalytic performance. First, dopants can generate localized states between forbidden bands and one advantage is fusing of dopant-occupied positions with VB and upshifting of valence band maximum (VBM). Such two diverse techniques normally originate from different doping types, where surface doping will cause development of localized states and uniform doping will promote VBM [148]. As mobility of h^+^s in localized states is slow and after that restricts photo-catalytic efficiency, offering uniform allocation of dopant to upshift VBM and encourage h^+^s relocation is preferred much. The atomic thickness of the 2DMs facilitated a uniform doping because little doping depth is specifically needed [149]. By tailoring O_2_-atoms for 2D ZnIn_2_S_4_ NSs to replace sulfur atoms lattice, electronic configuration suffered by diverse differences from the pristine ZnIn_2_S_4_ NSs [150]. So, the DFT-based calculations showed that O_2_-doping effectively reinforces DOS at VBM versus pristine ZnIn_2_S_4_, enlightening creation of enhanced charge density around VBM. Both of CB and VB edge in O-doped ZnIn_2_S_4_ showed the upshift concerning ZnIn_2_S_4_ NSs, as verified through UV–Vis absorption spectrum and XPS VB spectrum. It enhanced CBM as well as improved the VB distance across obtained with very superior mobility and improved the expenditure of photo-generated h^+^s, thus to support H_2_ production. Wang and co-authors studied that C-atoms doping can efficiently refrain electronic configuration of h-BN (Fig. 11) [151]. So, the DFT simulation showed that BG of h-BN was obtained to be 4.56 eV. After C-doping in structure, BG was notably reduced. Regarding B_11_C_12_N_9_ compound, BG was narrowed to 2.00 eV, through VB and CB edges which mostly consist of C_2*p*_ orbitals. B_11_C_12_N_9_ VB top states were not localized in comparison with pure BN. Taking advantage of C-doping with *sp*^2^-delocalization system, ultra-thin C-BN NSs with thickness of almost 3–4 nm were achieved and displayed an outstanding vis-light photo-catalytic performance to evolution of H_2_ and reduction of CO_2_. Considering insulator characteristic of pristine h-BN, it is illustrated that nonmetal doping can endorse photo-catalytic performance and also create promising photo-catalytic performance.

### Defect Engineering

Aside from doping, defect engineering also demonstrates an important effect on ultra-thin 2DMs, in case of photo-catalysis. Owing to 2D atomic size thin structure, in the presence of defects, it has strong influence on fundamental properties, in spite of a very low-level doping. In relation to huge surface defects formation in the bulk materials, ultra-thin 2DMsNSs with relatively small atomic escape energy can propose an important chance to get a range of defects. So, it is necessary to construct surface defects, for instance anion, cation vacancies, pits, vacancy association, and distortions, to efficiently optimize electronic configuration of ultra-thin 2D photo-catalysts.

#### Anion Vacancies

**Fig. 12:**
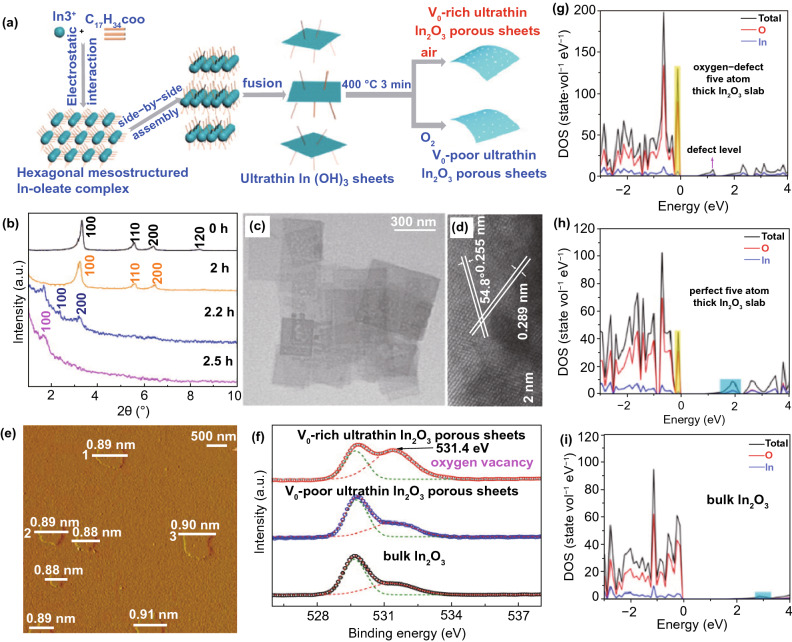
**a** Scheme showing the development of VO-rich/VO-poor atomically thin In_2_O_3_ porous NSs under special environment. **b** Time-dependent small-angle XRD patterns for the as-synthesized precursors. **c–f** Characterizations for the VO-rich atomically thin In_2_O_3_ porous NSs obtained via rapid thermal heat treatment of In(OH)_3_ NSs in air. **c**, **d** TEM/HR-TEM image. **e** AFM analysis. **f** O 1s XPS spectra. **g** Electron spins resonance spectrum. **h** Simulated DOS of O_2_ defect five-atom-thickness In_2_O_3_ slab. **i** ideal five-atom-thickness In_2_O_3_ slab. Adapted with permission from Ref. [84]

In anion vacancy type, V_O_ was broadly studied due to its small creation energy and prevalence in the oxide materials [152]. For example, Fengcai Lei et al. [84] studied that by fast heating of intermediate In(OH)_3_ NSs in O_2_ or air, V_o_-rich and V_o_-deficient In_2_O_3_ NSs were formed in fully controlled way, respectively. Figure 12 shows the AFM image, which showed thickness of In_2_O_3_ NSs to be almost 0.9 nm, enlightening controlled formation of In_2_O_3_ materials with atomic thickness. ESR and XPS spectrum results showed the presence of V_o_. The observed 531.4 eV peak showed V_o_-rich ultra-thin In_2_O_3_ NSs, which have maximum peak area, signifying that more V_O_-rich ultra-thin In_2_O_3_ NSs were formed compared to V_o_-poor ultra-thin In_2_O_3_ NSs and bulk counterpart. Moreover, sharp V_o_ signal at *g* = 2.004 in ESR spectrum was also observed that shows V_o_-rich In_2_O_3_ NS sample holds the highest level of V_o_. In V_o_ engineering, electronic configuration of In_2_O_3_ NSs with rich V_o_ will experience noticeable change. As authorized through DRS analysis and the XPS VB, spectrum, V_o_-rich In_2_O_3_ NSs showed a narrowed BG and upshifted VB edge. DFT simulations clearly show that enhanced DOS at VBM was created and a novel defect concentration showed V_o_-rich In_2_O_3_ than V_o_-poor In_2_O_3_ NSs. So, the V_o_-rich In_2_O_3_ NSs acquired higher carrier level and enhanced electric field in space charge regions. The e^−^s were further simply excited into CB in irradiation. Therefore, V_o_-rich In_2_O_3_ NSs showed 2.5 and 15 times enhanced photo-catalytic performance as compared to V_o_-poor In_2_O_3_ NSs as well as bulk In_2_O_3_, correspondingly, for H_2_O oxidation. Such outcomes certainly verified the efficient role of anion vacancy in electronic configuration engineering.

#### Cation Vacancies

**Fig. 13:**
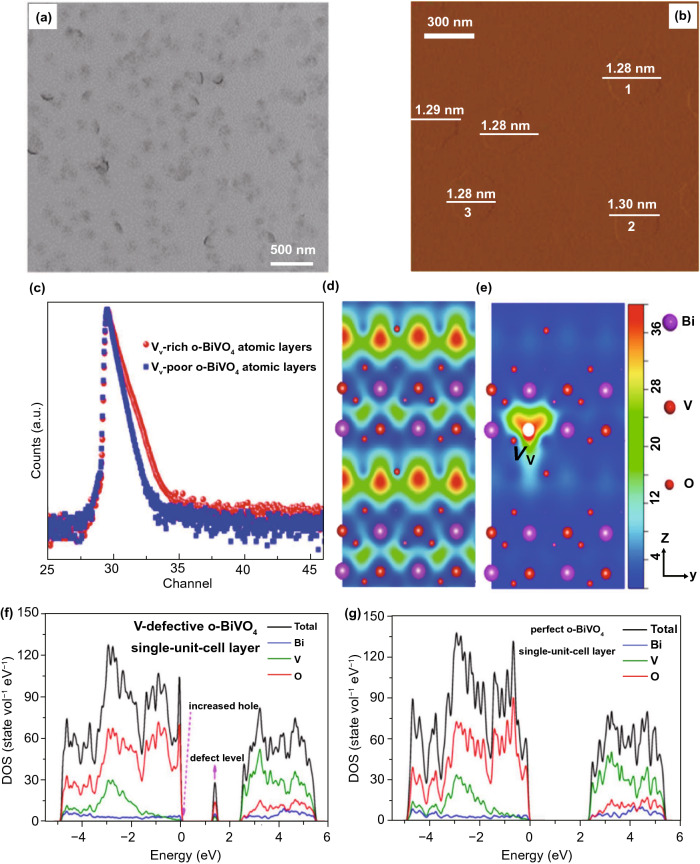
**a**, **b** TEM and AFM analysis of one-unit-cell thickness Vv-rich o-BiVO_4_. **C–e** Defects study of V_v_-rich and V_v_-poor o-BiVO_4_ atomic layers. **c** Positron duration spectra. **d**, **e** Scheme of entrapped positrons. **f**, **g** DOSs calculation of the V defects in o-BiVO_4_ single-unit-cell layer slab and pure o-BiVO_4_ single-unit-cell layer slab (**f**), along [001] direction (**g**). Adapted with permission from Ref. [154]

Besides anion, cation vacancies are extremely efficient approach to cause useful electronic structure modification of ultra-thin nano-structure due to multifarious electron arrangement and orbit. For example, vanadium (V) vacancies (V_v_) were initiated in single-unit-cell BiVO_4_ NSs (1.28 nm) along diverse quantities (Fig. 13) [153]. The atomic level concentration of the V_v_ was done through positron annihilation spectrometry (PAS) and X-ray fluorescence (XRF). For BiVO_4_ the shortest life component (*τ*_1_, around 200 ps) approved from PAS that trapped at V_v_, helpful for subsistence of V_v_ for two samples. Comparative positron intensity duration for V_v_-rich BiVO_4_ NSs was denoted for higher V_v_ level. Furthermore, elemental ratio of *t* V and Bi was verified to be 0.914 and 0.976 in V_v_-rich BiVO_4_ NSs and V_v_-poor BiVO_4_ NSs, in that order proposing concentration difference of V_v_. Benefiting from incidence of V_v_, a novel defect level can be produced in BG of BiVO_4_, as confirmed via DFT simulation, which leads e^−^s further capable to be excited into C.B. Additionally, higher DOS at VB edge was achieved because of V_v_ engineering. So, engineered vacancies increased the light harvest and promoted electronic conductivity for V_v_-rich BiVO_4_. Simultaneously, abundant V_v_ allowed an efficient charge separation that prolonged carriers’ lifetime from 74.5 to 143.6 ns. Taking advantages from such V_v_, an advanced photo-catalytic performance was attained from methanol synthesis rate up to 398.3 µmol g^−1^ h^−1^.

#### Associated Vacancies

**Fig. 14:**
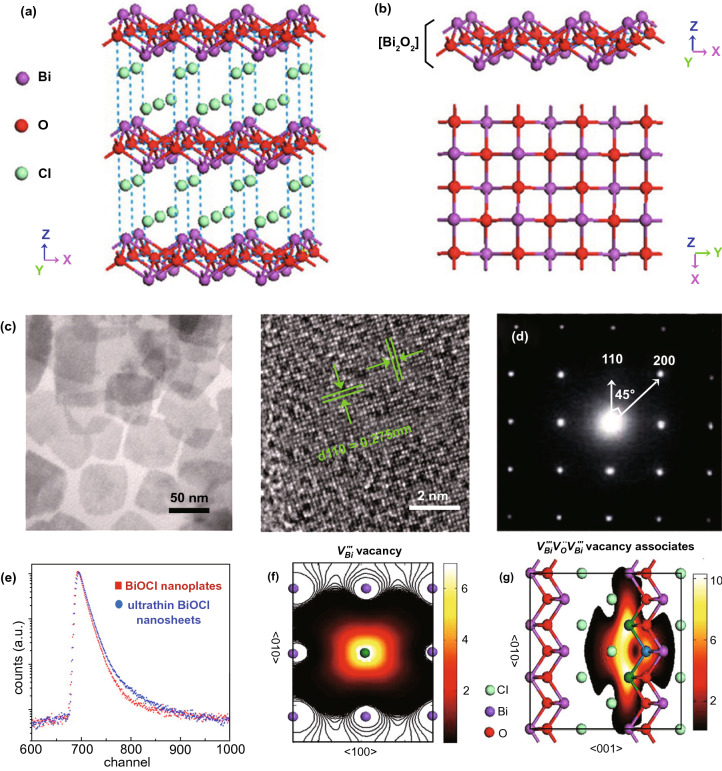
Scheme showing the crystal structure of BiOCl. **a** 3D-projection. **b**, **c** [Bi_2_O_2_]^2+^ layers along with the [010] and [001] directions, correspondingly. **d** TEM/HR-TEM of BiOCl NSs. **e** Positron lifetime spectra of ultra-thin BiOCl NSs and BiOCl NPs. **f**, **g** Scheme showing trapped positrons of VBi″′ defect and VBi″′VO^··^VBi″′-associated vacancy, correspondingly. Adapted with permission from Ref. [119]

The lost surface atoms not just introduced mono-vacancy but also vacancies associated were able to appear. As a result of multi-atomic vacancy coupling, vacancy associated can strongly engineer electronic structure and cause amazing electronic performance. For instance, triple vacancy of VBi″′VO^··^VBi″′ were built in ultra-thin BiOCl NSs (thickness = 2.7 nm) with dimension engineering (Fig. 14) [119]. Generated associated triple vacancy VBi″′VO^··^VBi″′ was verified through PAS. When Bi-atoms’ outer surface was exposed in BiOCl crystal configuration, it is very likely to break out from lattice to make vacancy. While thickness was decreased to an atomic size, O_2_-atoms that linked to Bi-atoms in an internal layer also escaped more effortlessly. So, control defects in BiOCl nano-plates were separated VBi″′, whereas that changed its associated vacancy VBi″′VO^··^VBi″′ in ultra-thin BiOCl NSs. Different defect types certainly affect the electronic structure that guarantees BiOCl NSs with enhanced adsorption of RhB molecules due to further negative charge. Benefiting from defect types changing from VBi″′ to VBi″′VO^··^VBi″′, ultra-thin BiOCl NSs showed both upshifted CB and VB potentials that favor charge mobility and therefore allowed enhanced separation of (e^−^–h^+^)-pairs. Therefore, ultra-thin BiOCl NSs showed great solar photo-catalytic activity toward removal of pollutants.

#### Distortion

Besides the vacancies, other defects, for instance, distortions and pits were also sufficient policies for changing electronic structure of the ultra-thin 2D photo-catalysts. For instance, O’Hare and his research team [99] formed NiTi-LDH NSs through controlled thickness during reverse micro-emulsion approach. As obtained NiTi-LDH NSs, local atomic arrangement was examined through the X-ray absorption near-edge spectroscopy (XANES). While thickness significantly decreased, the titanium cation (Ti^4+^) with lowered oxidation state was achieved in NiTi-LDH NSs (≈ 2 nm thickness), whereas it is approximately completely Ti^4+^ in bulk NiTi-LDH. In comparison with bulk NiTi-LDH, lower coordination numbers of Ti-cations were seeing in NiTi-LDH NSs and experience severs crystal deformations. Consequently, NiTi-LDH NSs showed distinguishing electronic crystal lattice and enhanced e^−^ transfer effectiveness comparative to bulk NiTi-LDH.

#### Pits

Pits formation gives a good technique to enhance surface defects which produced more coordinated unsaturated atoms with dangling bonds around pits in NSs. Through thermal treatment of bulk g-C_3_N_4_ inNH_3_ environment, NSs are efficiently exfoliated and many in-plane pits were built in g-C_3_N_4_ NSs [124]. In-plane pits formation damaged g-C_3_N_4_ plane structure and supplies promising active sites with dangling bonds. These dangling bonds serve as cross-plane diffusion pathways to speed up mass transfer and charge diffusion. Furthermore, formed pits are promoted as a result in creation of C-vacancies owing to unbalance structure. So, g-C_3_N_4_ NSs showed improved BG and absolute light absorption area matching with bulk counterpart. Simultaneously, pit-rich g-C_3_N_4_ NSs guarantee superior CB and VB potential and give advanced e^−^ donor density. Thus, 20 times high photo-catalytic H_2_-evolution activity was attained in vis-light irradiation. The aforementioned investigation results give a new and deep insight to comprehend method of action of surface defects in supporting photo-catalytic activity from energy band structure, surface charge, and SASs. Usually, as relative to defect poor counterpart, engineered defects in ultra-thin 2DMs can alter electronic configuration, generally with improved DOS at edge of VB or CB, or even create a novel defect concentration in between forbidden band. Generally, light absorption possibility of genuine materials with redshift and hence light harvesting capability can be improved. At surface, limited charge density can also be altered and might make possible adsorption and activation of the target molecules. Simultaneously, formed surface defects can develop carrier level in the photo-catalysts and provide charge separation centers to entrap the carriers, encouraging consumption effectiveness of h^+^s and e^−^s for analogous interfacial redox responses.

### Anisotropic Effects in Catalysis

Photo-catalytic routes could show vital to sustainable manufacturing of fuels and chemicals necessary for carbon–neutral society. An (e^−^–h^+^) recombination is a serious issue that has till date limited efficiency majority of potential photo-catalysts. Therefore, Matteo Cargnello et al. [155] showed the efficiency of anisotropy in enhancing charge separation and thus increasing activity of TiO_2_ photo-catalytic method. Particularly, they showed that H_2_ fabrication in homogeneous, 1D brookite-TiO_2_ nano-rods was highly improved through engineering their length. Utilizing respective characterization techniques to separately investigate excited e^−^s and h^+^s, linked high observed reaction rates to anisotropic arrangement support competent carrier use. The QY for H_2_-fabrication from C_2_H_5_OH, C_3_H_8_O_3_, and C_6_H_12_O_6_ as high as 65%, 35%, and 6%, correspondingly, showed generalization of this method for enhancing the photo-activity of SC-NMs for a broad range of reaction systems.

## Hybridization

Unlike bulk materials, 2DMs have become a hot topic in academic field due to their atomic layer thickness, broadband absorption, and ultrafast optical response, which have been widely applied in the ultrafast laser generation [156–183], optical switching and modulators [184–197], optoelectronics devices [183, 198–203], and biosensor and biotherapy [14, 44, 204–214]. 2DMs have an ultra-large SSA to make sure that surface state is even more significant compared to bulk inside. Photo-generated charge carriers will be distributed at surface to engage in the redox reactions, to be discussed next. So, surface hybridization to present size element to support consumption effectiveness of charge carriers is enviable below precondition of 2D arrangement. Consistent with surface hybridization, numerous representative hybridizations with 2D structure are introduced, e.g., QDs/2DMs, single atoms/2DMs, molecular/2DMs, and 2D–2D stacked materials.

### Quantum Dots/2DMs Hybridization

Coordinated unsaturated surface atoms of NPs have dangling bonds that used energy. To further decrease NPs’ size, bigger part of surface atoms compared with total atoms will be attained and their regular atom binding energy must be high. So, if the size of NPs can control the QDs and modify 2DMs, interfacial strong coupling among them can be manufactured. Furthermore, QDs can show high dispersion on 2DMs because of their small size; those probably effective co-catalysts furthermore enhance the photo-catalytic activity. To develop operation effectiveness of Ag, Ag-QDs with size > 5 nm were formed. After hybridization with BiOBr NSs, photo-catalytic activity has been significantly boosted to degrade tetracycline hydrochloride, ciprofloxacin (CIP), and rhodamine B after vis-light irradiation. It was studied that tailored Ag-QDs activate molecular O_2_ through hot e^−^ which decrease after vis-light exposure. The Ag-QDs can concurrently provide charge separation centers, adsorption centers, and photo-catalytic reaction centers, which are dependable on enhanced photo-catalytic efficiency. To decrease service of noble metals, non-noble metals or even metals-free QDs are taken as other choice. Also, thickness of subjected 2DMs can be further decreased to SL, in order to get improved dispersion and interfacial contact. For example, nitrogen-doped carbon QDs (N-CQDs) through 3 nm size were formed by facile hydrothermal method and later modified at BiOI NSs’ atomically thin surface [120]. The AFM analysis showed that average thickness of BiOI was almost 0.9 nm, proposing SL arrangement.

**Fig. 15:**
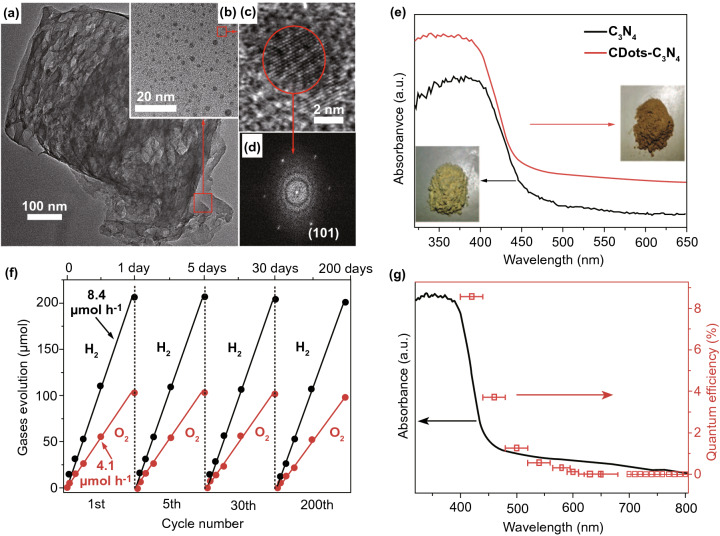
**a**–**d** TEM of C-QDs/C_3_N_4_. **e** UV–Vis absorption spectrum of C_3_N_4_ and C-QDs/C_3_N_4_ photo-catalysts. **f** Time period of O_2_ and H_2_ production from H_2_O after vis-light irradiation catalyzed via C-QDs/C_3_N_4_. **g** Wavelength-based QE (red dots) of H_2_O splitting via C-QDs/C_3_N_4_. Adapted with permission from Ref. [215]

After N-CQDs, the N-CQDs/BiOI matters introduction displayed significantly extended lifetime of photo-generated charge carriers, as showed through time-resolved transient photo-luminescence (PL) decay and immediate photo-current. BiOI atomic-level configuration makes sure prominently quick bulk charge diffusion to surface and conjugated *π*-modified N-CQDs configuration, which effectively endorse surface charge separation, resulting in longer carrier duration. Therefore, the photo-catalytic activity and the N-CQDs/BiOI materials active species concentration enhanced considerably. Kang and co-authors also confirmed that C-QDs can work as chemical catalyst to really improve the photo-catalytic H_2_O splitting via C_3_N_4_ (Fig. 15) [215]. Different from traditional single step 4e^−^ reaction from H_2_O splitting, 2e^−^/2e^−^ two-step path was followed by the C-QDs-C_3_N_4_, where C_3_N_4_ is added to transfer H_2_O to H_2_ and H_2_O_2_. Moreover, C-QDs are accountable in H_2_O_2_ decomposition and O_2_ evolution. Therefore, outstanding photo-catalytic H_2_O splitting effectiveness can be obtained, with 2.0% solar to H_2_ efficiency and robust stability in 200 recycle run after 200 days. Similarly, there are also some other systems concerning QD/2DMs hybridization to further enhance photo-catalytic efficiency, for example CdSe QDs, Zn–Ag–In–S QDs, NiS_2_ QDs, and so on [216]. Such findings certainly verified superiority of QD modification, and QD/2D arrangement might be efficient substitute for configuration to get improved photo-catalytic performance.

### Single Atoms/2DMs Hybridization

To further enhance photo-catalytic activity, size reduction of NPs to single atoms is possibly a probable plan. However, fraction of unsaturated coordination bonds of monoatom maximizes and enables a strong surface effect [217]. The first studied monoatom-based catalysis by Zhang et al. [218] became an interested topic in photo-catalysis direction. The monoatom-based photo-catalyst was based on isolated single atom on support materials surface, in the form of dispersion or coordination. Monoatoms-based strategy can improve photo-catalytic activity and offer another technique to alter the selectivity. As well, active single atom, chemical bonding in metal single atom, and 2DM NSs-based supports have become a strong and simpler charge transfer method. So, it is very attractive to create a single-atom/2DMs hybridization to obtain a high photo-catalytic activity [219]. Wu and co-workers [220] studied single Pt-atom as co-catalysts to advance photo-catalytic H_2_ evolution activity of C_3_N_4_ NSs. A simple liquid phase reaction with C_3_N_4_ and H_2_PtCl_6_-coupled low-temperature annealing was applied to form Pt-single atoms/C_3_N_4_. High-angle annular dark-field STEM (HAADF-STEM) was utilized to establish allocation and arrangement of Pt. The individual clear spots matching to Pt-atoms were viewed to be consistently dispersed on g-C_3_N_4_, with ~ 99.4% Pt size of > 0.2 nm, showing a Pt subsist completely as monoatoms. On the other hand, when the Pt loading quantity reaches 0.38%, the Pt-atoms dispersion was denser and formed numerous sub-nanometer clusters. Extended X-ray absorption fine structure (EXAFS) spectroscopy was utilized to investigate local atomic configuration of the Pt/C_3_N_4_. The Pt-atoms coordination number was about 5, through bond distance of ~ 2.03 Å, exposing those Pt-atoms that were dispersed on the top of C_3_N_4_ system. Following production of single Pt-atom/C_3_N_4_ arrangement, photo-catalytic H_2_-evolution activity was significantly enhanced. The H_2_-evolution rate of Pt/C_3_N_4_ (0.16 wt% Pt loading) achieved was almost 318 µmol h^−1^, about 50 times advanced, as compared to pristine-C_3_N_4_. Simultaneously, single Pt-atom/C_3_N_4_ showed admirable stability for H_2_-evolution and isolated single Pt-atom still stays at C_3_N_4_ after circulations. The desirable quality of UTAS, surface trap states of C_3_N_4_ verified was basically changed because of isolated single Pt-atom that extends the carrier duration and provides more chances for e^−^s to engage in H^+^-reduction method. Moreover, separated single Rh-atoms were spread on 2D-TiO_2_ NSs along with homogeneous 0.7 nm thickness via calcination, protonation, and coupled exfoliation method [221]. In HAADF-STEM image, separated brightest spots were observed that showed Rh-atoms but intermediate brightness spots symbolized Ti-atoms. The EXAFS showed that Rh species in single Rh-atoms/TiO_2_ displayed an analogous chemical setting as Rh_2_O_3_ showed bonding to O-atoms which was hence oxidized. Mono-Rh-atom co-catalysts were served as a reaction core for photo-catalytic-based H_2_-evolution, consistent with DFT simulations. So, H_2_-evolution rate was boosted 10 times as compared to pure TiO_2_ NSs. Although single atoms were engaged in catalysis, there were also existed numerous matters to be determined. Normally, the sustained content of monoatom was comparatively small and noble metal single-atom showed main types. It is very attractive to boost quantity of supported metal atoms with isolated single-atom arrangement and broaden it to other non-noble metals. Moreover, it is required to promote the stability, in order to meet potential industrial uses. The facts that single isolated metal atoms had high surface energy were shown, in which isolated metal atoms strongly cooperate with support surfaces. Through influenced metal atoms interactions with surface defects (elevated energy sites) on support, hybridization energy scheme might turn into a local minimum. Consequently, mono-metal atoms can be fastened as well as kept stable. Particularly in ultra-thin 2DMs, surface defects are liable to be generated because of ultra-large SSA and minute atomic flee energy. So, it is possible to construct monoatom anchored surface DR ultra-thin 2D arrangement to elevate photo-catalytic activity.

### Molecular/2DMs Hybridization

Despite single isolated atoms, single molecule materials can also be used to engineer electronic structure by acting as a co-catalyst to enhance photo-catalytic activity [222]. Profiting from sub-nano-pores in C_3_N_4_, molecular TiO_2_ was included into C_3_N_4_ NSs by facile polycondensation of precursors with dicyandiamide and TiO_2_-ions [223]. The morphology of clean ultra-thin TiO-C_3_N_4_ NSs was obtained and confirmed by TEM analysis, with thickness of about 3−3.3 nm. From HAADF-STEM analysis and elemental mapping, TiO_2_ was originated to be consistently dispersed on C_3_N_4_ framework with isolated format. These results suggested that molecular TiO_2_ was effectively built in C_3_N_4_ framework equally. Local Ti–O geometrical and electronic structures in sub-nano-pores of C_3_N_4_ NSs were found through XAFS. Usually, anatase-TiO_2_ displayed well-defined triple pre-edge characteristics that can be recognized to distort TiO_6_ pattern with six coordinated O_2_-atoms. Dissimilar from TiO_2_ result, Ti–O in C_3_N_4_ exhibited a single pre-edge feature with non-symmetric structure. The EXAFS showed that Ti-atoms were located in C_3_N_4_ h^+^s that was coordinated with six N_2_-atoms in C_3_N_4_ and one O_2_ atom out of plane in C_3_N_4_. Benefiting from TiO_2_ molecule insertion, TiO-C_3_N_4_ NSs displayed a narrowed BG in comparison with pristine C_3_N_4_ with a reduction in CB position. It is resultant from more electron involvement of Ti–O into C_3_N_4_ and enhances *π*–e^−^ delocalization in conjugated structure. TiO-C_3_N_4_-engineered electronic structure can also enhance charge carrier separation. Therefore, TiO-C_3_N_4_ enhanced the photo-catalytic performance for ·OH generation and removal of pollutant. In photo-catalytic method, h^+^s transfer slower kinetics which brings massive carrier recombination in Achilles’ heel of photo-catalytic conversion activity. Even though ultra-thin 2D-structure permits quick charge migration in bulk phase, lack of surface charge separation centers will also spoil overall photo-catalytic activity. Utilizing strategy to encourage surface charge separation, particularly h^+^s transfer is extremely required. Instead, it looks feasible to utilize H_2_O soluble molecular materials as homogeneous co-catalyst and hence also optimize photo-catalytic performance. Wu and his research team [220] formed H_2_O-soluble molecular trifluoroacetic acid (TFA) as co-catalysts to enhance photo-catalytic H_2_-evolution performance of K_4_Nb_6_O_17_ NSs. Taking advantage of reversible redox couple TFA·/TFA− as well as high active intermolecular radical responses, TFA molecule served as strong h^+^s-shuttle, allowing efficient move of the photo-generated h^+^s and ensuing elevated charge separation effectiveness. The TFA increment enhanced H_2_-evolution rate regularly and the maximum rate reached 6344 µmol g^−1^ h^−1^, when TFA/K_4_Nb_6_O_17_ molar ratio was 25.6. This optimum H_2_ yielding rate was ~ 32 times higher compared to pristine K_4_Nb_6_O_17_ NSs, certainly signifying this molecular co-catalyst. As solid-state co-catalysts, they are restricted from limited contact areas in co-catalysts and host photo-catalyst, and surface charge separation cannot be completely definite. When overmuch solid-state co-catalysts were anchored on photo-catalyst, the SASs will be covered that deficient in the satisfactory active sites easy to get reactant molecules. As a result, H_2_O-soluble molecular materials such as molecular co-catalysts can equally disperse in the solution and give greatest available area to host photo-catalysts. So, developed molecular co-catalyst approaches perhaps are a possible way in a competent separation of photo-generated carriers and therefore improve photo-catalytic performance.

### 2D–2D Stacking Materials Hybridization

**Fig. 16:**
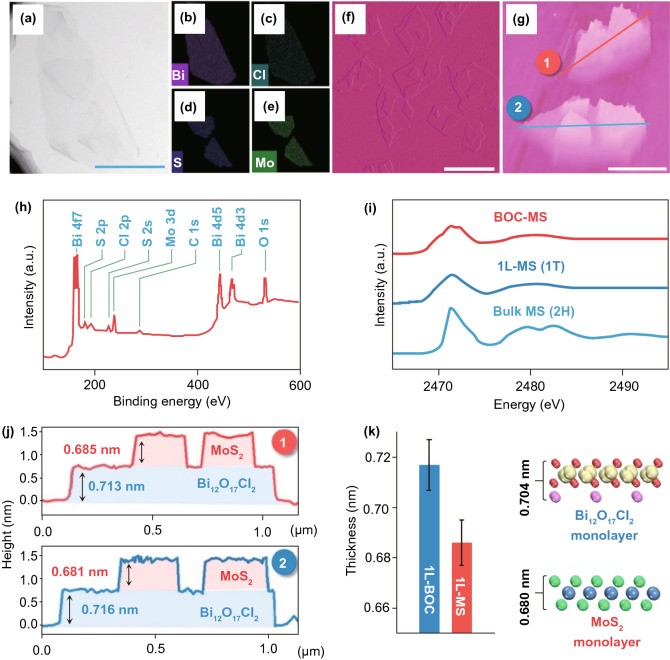
**a** TEM top view analysis. **b–e** elemental mapping images, **f** XPS spectrum. **h**, **i** AFM analysis. **m** TEM images side view. **n** Atomic resolution HAADF-STEM image. **o–s** Respective EELS elemental maps of BOC-MS. **g** S K-edge XANES spectrum of BOC-MS, 1L-MS, and bulk MS. **j** Height profiles along lines in **i**. **k** Comparison of 1L-BOC and 1L-MS in BOC-MS average thicknesses. Error bars in (**k**) show s.d. over 100 self-sufficient AFM calculations. SLs MoS_2_ and Bi_12_O_17_C_l2_ theoretical thicknesses. Adapted with permission from Ref. [224]

While constructing 2D–2D stacks, it is a largely applied method to boost photo-catalytic efficiency. Particularly for layered materials, lattice mismatch was reduced due to comparable layered structures, and 2D–2D stacking with close associates was formed. For example, Zhang and co-workers [139] studied SL Bi_12_O_17_C_l2_ with surface V_o_ via Li-intercalation-based exfoliation approach. Afterward, SLs MoS_2_ NSs were assembled onto SL-Bi_12_O_17_C_l2_ through surface V_v_ and build Bi-S bonds in Bi_12_O_17_Cl_2_ and MoS_2_. TEM image well matched with an elemental mapping results proposed that numerous tiny MoS_2_ NSs were strongly anchored on a large Bi_12_O_17_C_l2_ NS to make 2D stacking hetero-structure (Fig. 16); normal thickness of large size and small size NSs was ~ 0.717 and 0.686 nm that concurred with Bi_12_O_17_Cl_2_ and MoS_2_ SLs, in that order.

The atomic resolution HAADF-STEM analysis and resultant EELS elemental mapping showed that it was obviously studied and SLs-MoS_2_ were anchored selectively on (Bi_12_O_17_) end faces to construct (Cl_2_)–(Bi_12_O_17_)–(MoS_2_). As charge density surrounding (Bi_12_O_17_)^2+^ layer was superior compared to (Cl_2_)^2−^ layer, photo-generated e^−^s and h^+^s were ambitious to (Bi_12_O_17_)^2+^ and (Cl_2_)^+^ end faces under irradiation, correspondingly. Photo-generated e–s flowed in between MoS_2_ SLs through formed Bi-S bonds and enable efficient charge separation (ultra-long duration of carrier 3446 ns), as proofed through TA spectroscopy. Taking advantage of atomic size thickness, efficient directed interface charge separation, and plentiful H_2_-evolution sites in MoS_2_, acquired MoS_2_/Bi_12_O_17_Cl_2_ bilayers displayed great vis-light photo-catalytic H_2_-evolution performance. Using ascorbic acid as h^+^s sacrificial agent, H_2_-evolution rate can turn up 33 mmol h^−1^ g^−1^ and quantum effectiveness of 36% at 420 nm. Other than MoS_2_/Bi_12_O_17_Cl_2_, there are several investigations about 2D stacking to optimize photo-catalytic efficiency, for instance NiO/Ca_2_Nb_3_O_10_ [225], MoS_2_/TiO_2_ [224], MoS_2_/CdS [226], WS_2_/CdS [226], MoS_2_/C_3_N_4_ [227], SnS_2_/C_3_N_4_ [228], Fe_2_O_3_/C_3_N_4_ [229], C_3_N_4_/Bi_4_O_5_I_2_, ZnCr-LDH/layered titanate [230], and ZnIn_2_S_4_/MoSe_2_. For example, a distinctive 2D stacking structure has more benefits. It increased the available area about planar interface in 2D/2D structures and reduced barriers for e^−^ transportation via co-catalyst, and therefore promoted the interfacial charge transfer development through e^−^ tunneling effect. Furthermore, these 2D thin layers can ease light blocking effect of co-catalyst; therefore, sufficient light can contact the host photo-catalyst. It is required to alter 2D-components and reinforce interfacial acting force to promote highly efficient 2D/2D photo-catalysts.

## Photo-catalytic Applications

Based on the aforementioned consequences, ultra-thin 2DMs displayed huge benefits for the photo-catalysis from microstructure, BG, electronic configuration, and surface nature. Thus far, advanced ultra-thin 2DMs NSs were functional as photo-catalysts for diverse photo-catalytic uses. So, development of flexible photo-catalytic uses by 2DMs for H_2_O oxidation, H_2_-evolution, CO_2_ reduction, N_2_-fixation, organic synthesis, and pollutants removal will be explained in detail in the next section.

### Water (H_2_O) Oxidation

Hydrogen (H_2_) that has the highest energy density is measured as one of the potential energy carriers for storing solar energy in chemical bond energy form between two H atoms. In between different methods for conversion of sunlight and H_2_O into H_2_, photoelectrochemical (PEC) H_2_O splitting using SC photo-electrodes has gained most interest due to three main benefits:

1. Production of H_2_ and O_2_ at respective electrodes that eradicate separation problems.
2. Operation potential under environmental conditions.
3. Potential for manufacturing of a system which consists of just stable and copious inorganic materials.

**Fig. 17:**
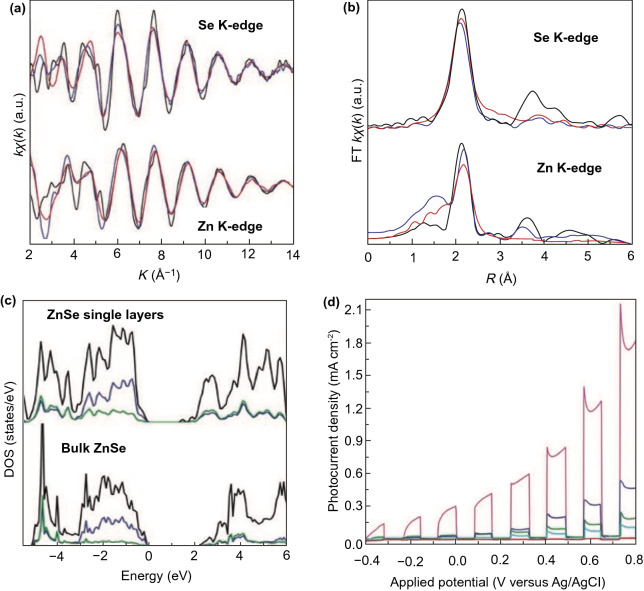
**a** Se and Zn K-edge extended XAFS oscillation function kχ(k). **b** Equivalent Fourier transforms; red, blue, and black lines show ZnSe SLs, ZnSe-pa SLs, and bulk ZnSe, in that order. **c** Simulated DOS; black, blue, and olive lines show total, Se sp, and Zn sp state densities, correspondingly. **d** Photo-current density versus utilized potential curves in chopped 300 W Xe-lamp irradiation. Adapted with permission from Ref. [233]

Designing vis-light-active SCs for H_2_O splitting needs an appropriate BG and band position, efficient charge separation, fast charge movement, and longtime durability in aqueous solutions. An attractive design approach to fulfill these requirements is to merge 2D materials (e.g., graphene, MoS_2_, g-C_3_N_4_) with appropriate SCs. It is usually known that H_2_ is very potential green fuels with benefits, for example high specific energy, multiple use approaches, and pollution-free combustion product. It displayed magnificent view in prospect of sustainable energy use, if it is created through sustainable skill. The photo-catalytic H_2_O splitting into H_2_ and O_2_ is viewed as Holy Grail infield of chemistry through just sustainable solar light as energy input, photo-catalysts as medium, and H_2_O as reaction source. As major forward step was accomplished, effectiveness of H_2_O splitting is still restricted in majority of photo-catalytic methods. Usually, H_2_O-oxidation is efficiency, limited method in photo-catalytic H_2_O splitting schemes because of complex four h^+^s complex redox method. So, it is highly in demand and imperative to propose photo-catalyst with robust solar H_2_O-oxidation method. Recent studies illustrated that 2DMs-based photo-catalysts are very capable choice for solar H_2_O-oxidation [231, 232]. The freestanding SLs ZnSe with four-atomic thickness was formed through ultrasonic exfoliation from lamellar hybrid intermediate (Zn_2_Se_2_)(pa) (pa represents n-propylamine) [231]. The U-XAFS, local atomic structures, and electronic configurations of the ZnSe SLs were studied. For Zn K-edge kχ_(k)_ oscillation curves as shown in (Fig. 17a), the ZnSe SLs displayed clear distinction comparative to ZnSe-pa SLs and bulk ZnSe, showing significant variation of local atomic arrangement. The R-space curves of ZnSe samples showed that peaks positioned at 2.11 and 3.63 Å were attributed to the nearest Zn–Se and next nearest Zn–Zn coordination in bulk ZnSe (Fig. 17b). While ZnSe size was decreased to an atomic level, local atomic configuration experiences outstanding changes. ZnSe peak was shifted to 2.17 Å, and next the nearest Zn–Zn distances (3.85 Å) were decreased. Simultaneously, Se–Se distances in SLs ZnSe NSs were extended from 4.012 to 4.11 Å of bulk ZnSe. Such findings certainly showed reality of surface distortion in SL structure that reduced surface energy and allowed exceptional stability of ZnSe SLs. Moreover, surface deformation of SL ZnSe will consequently be in the form of enhanced DOSs at CB edge that might further make sure a high charge carrier transfer rate (Fig. 17c).

**Fig. 18:**
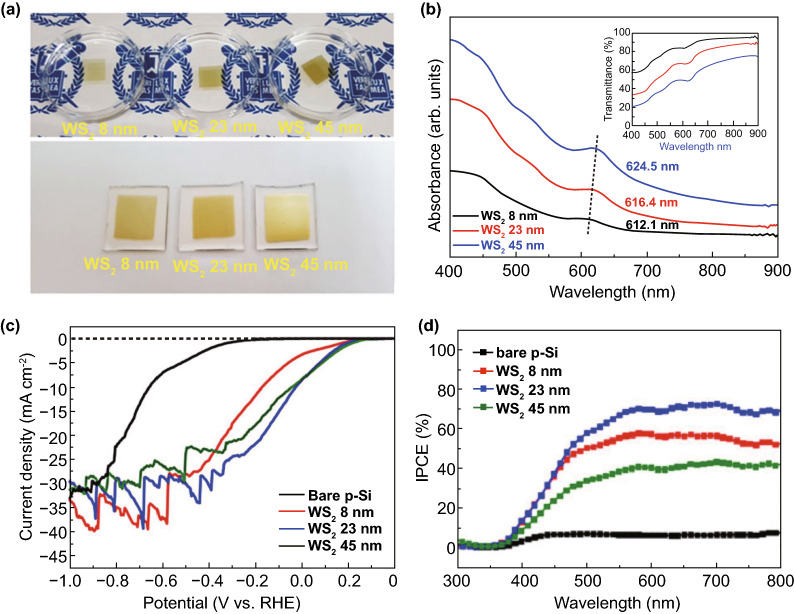
**a** Photographs of WS_2_ films on glass with different thicknesses. **b** UV–Vis absorbance of WS_2_ different thin films. **c** PEC performance illustrated as J–V polarization curves for WS_2_ different films size. **d** IPCE measurements of WS_2_ different thin films size on p-Si photo-cathodes. Adapted with permission from Ref. [238]

Taking advantages from SL structure with surface defects, ZnSe SLs show strong light absorption, enhanced charge separation effectiveness, and small charge transfer resistance. Consequently, SLs ZnSe NSs exhibited 195 times superior photo-catalytic performance compared to bulk ZnSe for H_2_O oxidation after Xe-lamp irradiation (Fig. 18d). As H_2_O oxidation to evolve O_2_ is completely h^+^-contributed reaction, enhancing h^+^-use rate perhaps is an efficient plan to enhance photo-catalytic H_2_O-oxidation. Liu et al. [136] created several pore constructions in ultra-thin WO_3_ NSs through fast-heating approach on earlier exfoliated WO_3_·2H_2_O NSs. As migration direction of the photo-generated h^+^s was along {001} [2–4, 16–26] facets in the *x*-direction in W–O–W chains on WO_3_ NSs, with long itinerant pathway the photo-generated h^+^s certainly undergo several recombinations of charge carriers, which acutely prevent photo-catalytic performance. The created pores efficiently shorten diffusion way of h^+^s and conduce to H_2_O oxidation to make O_2_ at WO_3_ surface. Additionally, plentiful dangling bond along pore environment created good circumstances to make easy chemisorptions of molecular reaction that finally boosted O_2_-evolution kinetics. As a result, 18 times superior photo-catalytic H_2_O oxidation was attained for pore-rich WO_3_ ultra-thin NSs comparative to bulk WO_3_. It shows significant strategy to promote conversion efficiency by using ultra-thin 2D configuration to the photo-catalytic H_2_O oxidation. Additionally, many other photo-catalysts with ultra-thin 2D-structure can also show an exceptional photo-catalytic behavior toward H_2_O oxidation, for instance SnS_2_ [234], SnSe [235], SnS, Fe_2_O_3_ [236], NiTi-LDH, and also engineered materials like In_2_O_3_ with V_o_ engineered [84], Co-doped In_2_S_3_ [145], pit-rich BiOCl [237], and so on.

### H_2_-Production

As innovative investigation for photo-catalytic H_2_-evolution based on TiO_2_ in 1972, photo-catalytic H_2_-evolution from H_2_O was at front position of chemistry research to solve worldwide energy problem. Many amazing catalytic materials were used in photo-catalytic H_2_-production; majority are still said to have quite small photo-catalytic effectiveness, which could not meet the necessities of realistic uses on large industrial scale. Current research for the 2DMs established that rising 2D-SCs with suitable energy BG were a talented choice to get outstanding H_2_-evolution performance [239]. In case of abundant SCs, the Cu_2_O with unique CB, positioned at ~ 0.7 V negative as compared to H_2_-evolution potential, is possibly a competent catalyst in H_2_-conversion from solar. To realize elevated H_2_-evolution efficiency, atomic-sized 2D-NSs, e.g., cubic Cu_2_O, were formed. The AFM study showed thickness of about 0.62 nm, related to four-atomic-level thickness of Cu_2_O in [01-1] direction. Therefore, surface energy of the cubic Cu_2_O was conformed to the order of (111) < (100) < (110). As (110) and (01-1) surfaces are equivalent facets (01-1), facet in atomic-level thin Cu_2_O NSs also showed a great surface energy that brings a good activity. From considerably reduced thickness, atomically thin Cu_2_O NSs’ electronic structure was distinct from bulk equivalent. Utilizing DFT simulations, an atomically thin Cu_2_O NSs showed really enhanced DOSs at edge of VB as compared to bulk Cu_2_O. At similar time, extended CB edge was also studied in 2D Cu_2_O NSs as compared to bulk Cu_2_O, enlightening that atomic-level thick Cu_2_O has high carrier mobility and small BG. Profiting from such advantages, above 36 times higher photo-catalytic H_2_-evolution rate was reached from 2D Cu_2_O NSs after vis-light irradiation. These results certainly illustrated that 2DMs can bring huge advantage for H_2_-evolution as well as a series of extraordinary activities. In addition to the advancement in H_2_-evolution, more modification in ultra-thin 2DMs structures was required. For instance, through doping O_2_ into ZnIn_2_S_4_ NSs, H_2_-evolution rate of O-doped ZnIn_2_S_4_ can attain 2120 µmol h^−1^ g^−1^ from aqueous solution containing 0.25 m Na_2_SO_3_ and 0.35 m Na_2_S after visible light illumination lacking any co-catalyst that was 4.5 times high as compared to pure ZnIn_2_S_4_ [150].

Local atomic structures of formed materials were studied by XAFS. The O_2_ doping in ZnIn_2_S_4_ NSs created high structure distortion through substitution of O_2_-atoms for sulfur. The engineered local atomic and electronic structure will experience observable deviation. The DFT simulation showed that O_2_ doping can boost DOS at VBM with respect to pure ZnIn_2_S_4_, signifying creation of enhanced charge density about VBM. XPS results showed the valence spectra and estimated BG, where CBM and VBM of O-doped ZnIn_2_S_4_ ultra-thin NSs show upshifting in comparison with pure ZnIn_2_S_4_. So, average recovery duration of carriers for O-doped ZnIn_2_S_4_ NSs was about 1.53 factors long-lasting relative to pure ZnIn_2_S_4_, attaining 110 ps. Hence, these advantages showed that O-doped ZnIn_2_S_4_ can show really enhanced photo-catalytic activity for H_2_-evolution. These findings showed that doping was an efficient policy to alter local electronic and atomic structure of 2DMs that can influence charge separation or migration and at the end optimizes H_2_-evolution.

Moreover, though considering major ultra-thin 2D-SCs NS materials lacks satisfactory H_2_-evolving sites, it is necessary to establish plentiful H_2_-evolution positions, for example, isolated Pt-atom or FL-TMDCs to more improve H_2_-evolution performance. Therefore, Wang and co-authors [227] studied FL MoS_2_ to C_3_N_4_ NSs to improve photo-catalytic H_2_-evolution. In the formation method, C_3_N_4_ was absorbed in (NH_4_)_2_MoS_4_ H_2_O solution and after that sulfidation was done through H_2_S gas, at 350 °C. C_3_N_4_ and MoS_2_ have equivalent layer formation, which can reduce lattice mismatch and help planar development of MoS_2_ slabs after using C_3_N_4_ surface. Consequently, an inorganic–organic 2D–2D stacking was formed using G-like thin-layer hetero-junctions. Plentiful H_2_-evolution sites were formed via FL MoS_2_ NSs. Moreover, dispersed MoS_2_ thin layers on C_3_N_4_ NSs surface can provide the superior effectiveness compared to multilayer MoS_2_ because of e-tunneling effect via MoS_2_ thin layers from reaction interfaces. On the basis of support charge separation and plentiful H_2_-evolving sites persuaded through FL MoS_2_ NSs, acquired MoS_2_/C_3_N_4_ 2D junctions exhibited better photo-catalytic H_2_-evolution activity in comparison with pristine C_3_N_4_. The 0.2 wt% MoS_2_ is the best sample that illustrated the highest H_2_-evolving rate, with an obvious 2.1% QY recorded at 420 nm. Moreover, WS_2_ can be utilized as a catalyst in WS_2_/p-type Si photo-cathode hetero-junctions. Kwon et al. [238] examined WS_2_/p-Si photo-cathode for photo-catalytic based HER. Figure 18a shows the as-prepared WS_2_ thin film color changes from yellow to brown with increasing thickness, whereas absorbance of film regularly enhances. However, absorption peak position almost remains constant (Fig. 18b). The PEC demonstrated that photo-catalytic HER performance for 23 nm WS_2_/p-Si showed the maximum current density of 8.375 mA cm^−2^ at 0 V and 72% incident photon to current conversion efficiency (IPCE) (Fig. 18c, d). So, it shows that merger of TMDs (WS_2_, MoS_2_), along traditional SCs, for example Si, is capable for efficient PEC H_2_O splitting.

**Fig. 19:**
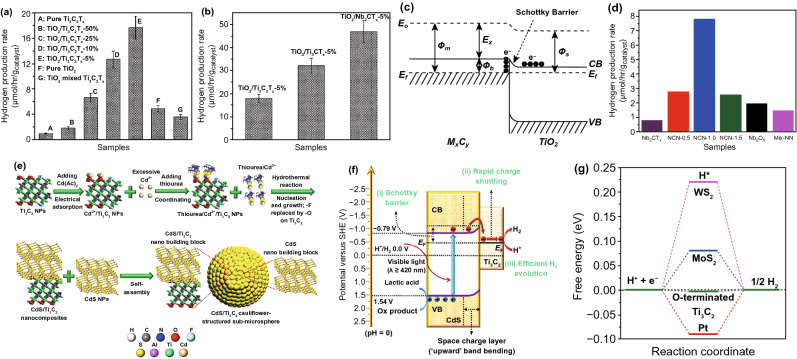
**a**–**c** Photo-catalytic H_2_ formation rates of TiO_2_/Ti_3_C_2_T_*x*_ and controlled samples. (**a**) Photo-catalytic HER activity of TiO_2_ loaded with particular metal carbide MXene co-catalysts (Ti_3_C_2_T_*x*_, Ti_2_CT_*x*_, and Nb_2_CT_*x*_), (**b**) formation of SB at MXene/TiO_2_ hetero-interface, and (**c**) Wang et al. [240]. **d** Photo-catalytic HER of Nb_2_O_5_/C/Nb_2_C samples (Tongming Su et al. [242]). **e–g** Fabrication method of CdS/Ti_3_C_2_ cauliflower-structured sub-microsphere (**e**), charge separation and transfer in CdS/Ti_3_C_2_ system after vis-light illumination (red and blue spheres illustrate photo-induced e^−^s and h^+^s, correspondingly) (**f**) and at equilibrium potential HER free energy diagram (**g**). Adapted with permission from Ref. [242]

Similarly, Wang et al. [240] studied physicochemical nature of Ti_3_C_2_T_*x*_ MXene coupled with TiO_2_ for photo-catalytic HER (Fig. 19a) [241]. The 5 wt% content of Ti_3_C_2_T_*x*_ in TiO_2_/Ti_3_C_2_T_*x*_ nano-composite showed a 400% augmentation for the photo-catalytic HER than rutile TiO_2_. Therefore, Ti_3_C_2_T_*x*_ gives a 2D-podium to interrelate with consistently fabricated TiO_2_ to make possible partition of photo-generated (e^−^–h^+^)-pairs to slow charge recombination. Fascinatingly, TiO_2_ NPs were well dispersed at Ti_3_C_2_T_*x*_ surface without harsh aggregation. Additionally, Ti_3_C_2_T_*x*_, Nb_2_CT_*x*_, and Ti_2_CT_*x*_ were also utilized as reducing co-catalysts after reacting with TiO_2_ and increase photo-activity in the HER [240]. Fascinatingly, Nb_2_CT_*x*_ and Ti_2_CT_*x*_ show better HER catalytic activity as compared to Ti_3_C_2_T_*x*_, while coupling with TiO_2_ as co-catalysts (Fig. 19b). It can be dragged through Schottky barrier (SB) and work function of every MXene, where Nb_2_CT_*x*_ shows the maximum work function (~ 4.1 eV) for direct proof of the highest HER activity. In reaction system, TiO_2_ surface was excited after irradiation with light to generate e^−^s and h^+^s. As a result of different Fermi levels of MXene and TiO_2_, photo-generated e^+^s was transferred from CB of TiO_2_ to MXene. Furthermore, adding positive charges in TiO_2_ and negative charges in MXene, CB and VB were turned upward (Fig. 19c), leading to form SB at TiO_2_/MXene hetero-interfaces to avoid e^−^s from relocating back to TiO_2_. In addition to SB and fast e^−^ shuttling, build-up of e^−^s on MXene will respond to H^+^-ions to create H_2_.

In addition, Su et al. [242] formed Nb_2_O_5_/C/Nb_2_C (MXene) hybrid nano-composites (NCN-*x*) through oxidizing Nb_2_CT_*x*_ surface at various durations (*x* = 0.5, 1, and 1.5 h) to produce a Nb_2_O_5_ layer via utilizing CO_2_ as gentle oxidant. Optimum duration of oxidation of 1 h photo-activity of NCN-1.0 for HER was four times higher as compared to pure Nb_2_O_5_ (Fig. 19d), which was created from close interfacial junction in conducting Nb_2_C co-catalysts and SC Nb_2_O_5_ for exceptional (e^−^–h^+^)-separation. This showed generation of transition metal carbides as feasible co-catalysts in solar to chemical energy conversion. Other than MO photo-catalysts, hybridization of TMDCs with MXene has also turned into an effervescent area of catalysis research in energy conversion. Qiao and co-workers also studied metal sulfide/Ti_3_C_2_ (metal sulfide: CdS, ZnS, and Zn_*x*_Cd_1−*x*_S) nano-hybrid photo-catalysts (Fig. 19e) [243]. The CdS/Ti_3_C_2_ composite records high vis-light HER photo-activity (14,342 mol h^−1^ g^−1^, 136.6 times that of bare CdS) with surprisingly large apparent QY value 40.1% at 420 nm, showing that it was one of the most excellent noble metal-free metal sulfide photo-catalytic system. The DFT simulations showed that O_2_-terminated Ti_3_C_2_ was talented co-catalyst derived from its marvelous HER activity, outstanding metallic conductance, and enviable Fermi levels. Basically, such investigation formed a novel view for scheming high-efficient and cost-efficient solar H_2_O splitting utilizing metal chalcogenide photo-catalysts and photo-electrodes. Moreover, MXene-based composite with metal-free g-C_3_N_4_ photo-catalysts has a new start of attention in kingdom of renewable energy formation. Shao et al. [244] formed a Ti_2_C/g-C_3_N_4_ photo-catalyst by use of thermal annealing of melamine with the 2D-Ti_2_C NSs for the outstanding HER activity. Optimum 0.4 wt% of Ti_2_C provides an elevated H_2_-production rate of 950 mol h^−1^ g^−1^ with apparent QY of 4.3% at 420 nm. Enhanced photo-catalytic efficiency was observed by efficient charge transfer and separation due to the existence of SB to decrease H^+^ to H_2_. Similarly, Sun et al. [75] also studied g-C_3_N_4_/Ti_3_C_2_T_*x*_ photo-catalysts, in which Ti_3_C_2_T_*x*_ with O-surface terminations enhances the separation of charges for improvement of 105% in the HER activity with apparent QY of 1.27%. The DFT simulations support it and showed that O-terminated Ti_3_C_2_ with 25% H-atoms presents obvious free energy as low as 0.011 eV [241]. Giorgio Carraro et al. [81] explored enhanced H_2_ evolution via photo-reforming of sustainable oxygenates using nano-structure Fe_2_O_3_ polymorphs. They studied that Fe(III) oxide polymorphs, β- and ε-Fe_2_O_3_, have notable performance in solar spectrum for H_2_ production from solutions of renewable oxygenates (i.e., ethanol, glycerol, glucose). For β-Fe_2_O_3_ and ε-Fe_2_O_3_, H_2_ evolution rates up to 225 and 125 mmol h^−1^ m^−2^ are gained, along significant better activities in regard to commonly study α-Fe_2_O_3_.

### Reduction of CO_2_

Other than photo-catalytic H_2_-evolution, CO_2_ reductions to produce hydrocarbon fuels over photo-catalysts were observed as an efficient way to concurrently reduce energy crisis and greenhouse cause. Basically, combustion of fossil fuel develops a great quantity of CO_2_, causes an increase in greenhouse effect due to unstoppable increase in CO_2_. Significantly, conversion of CO_2_ to valuable fuels is an alarming challenge in the recent time. Certainly, CO_2_ composed a fundamental C1 building element for chemical industries, but its thermodynamic stability and very high kinetic blocked its broad industrial uses. The undeniable solution for this mystery is to discover C-neutral fuels for low-C market to a sustainable future without environment disadvantages [245]. Usually, the CO_2_ molecules are very stable with C=O bond dissociation energy higher than ~ 750 kJ mol^−1^. Thus, in CO_2_ photo-reduction method, higher energy is required to split O=C=O structure, that is very demanding compared to H_2_O splitting into H_2_. Gateway for creation of a CO^2·−^ intermediate through single e^−^ transmission to activate a CO_2_ was observed as a rate-limited step to ensue proton-concerned reduction method. A theoretical potential of − 1.9 V versus NHE is needed for initial startup, and a superior over-potential is necessary for actual utilized potentials. Furthermore, multiple proton-coupled e^−^ transfer methods are concerned in CO_2_ activation and reaction ways were quite difficult and varied with the synthesis of different products. In accordance with diverse number of injected e^−^s (2e^−^, 4e^−^, 6e^−^, and 8e^−^), the products, for example, are CO, HCHO, CH_3_OH, and CH_4_, correspondingly. Moreover, competition exists for CO_2_ photo-reduction and H_2_O reduction, because H_2_O reduction to produce H_2_ is energetically more encouraging that confines products selectivity. Current research explained that ultra-thin 2DMs-based photo-catalysts also displayed outstanding CO_2_ photo-reduction. For instance, Bi_2_WO_6_ layers with single-unit-cell thickness were formed through a lamellar hybrid intermediate plan [246]. Sodium oleate was used to provide oleate ions, thus to relate to Bi^3+^ via electrostatic interaction. Afterward, lamellar Bi-oleate complexes were formed through self-assembly with tail-to-tail or head-to-head bilayer array of oleate ions to construct a meso-structure. While Na_2_WO_4_ was inserted and treated with hydrothermal method, Bi_2_WO_6_ was formed and lamellar meso-structure was self-exfoliated into a single-unit-cell layer. As-synthesized single-unit-cell Bi_2_WO_6_ layer was used in photo-catalyst for CO_2_ photo-reduction and a 300 W Xe lamp. The Bi_2_WO_6_ powder was suspended in H_2_O along highly pure CO_2_ gas constantly bubbled during solution for measurements. An average rate of 75 µmol g^−1^ h^−1^ was obtained in methanol synthesis over single-unit-cell Bi_2_WO_6_ layers for 5-h analysis that was almost 3 and 125 times higher comparative to Bi_2_WO_6_ nano-crystals and bulk Bi_2_WO_6_ correspondingly. Following six cycles, photo-reduction effectiveness remains the same and is not effected by any deterioration and shows an outstanding photo-stability. Recent research initiates that an excellent photo-catalytic performance was resulting from the novel geometrical configuration of single-unit-cell Bi_2_WO_6_ layers, as follows:

1. Initially ultra-large SSAs of single-unit-cell Bi_2_WO_6_ layers make sure 3 factors higher capacitance in CO_2_ adsorption that was a significant tip for CO_2_ activation and reduction.
2. The single-unit-cell thicknesses give advance charge separation and extend carrier duration as testified through time-resolved fluorescence emission spectrum calculations.
3. Single-unit-cell thickness carries almost higher DOS at CB edge as well as boosted surface charge density and hence promoted the 2D-conductivity.

**Fig. 20:**
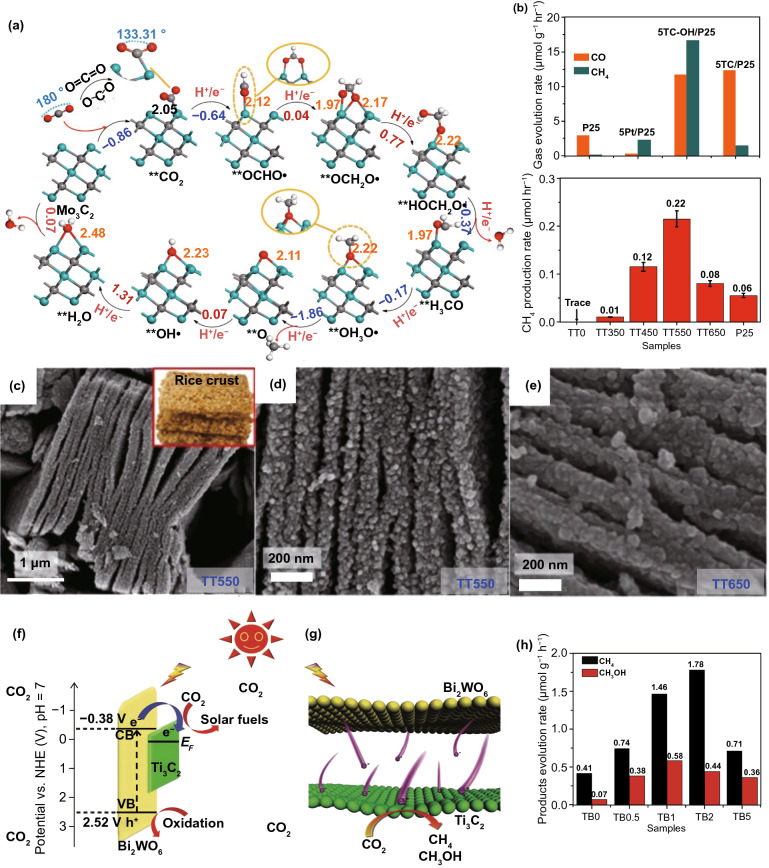
**a** Lowest amount energy routes (PBE/DFT-D3 calculations) pursued for CO_2_ into *CH_4_ and **H_2_O conversion, catalyzed by Mo_3_C_2_. Gray, lilac, red, and white spheres indicate C, Mo, O, and H atoms, correspondingly (Li et al. [248]). **b** Photo-catalytic CO and CH_4_ evolution rates over P25, 5Pt/P25, 5TC/P25, and 5TC-OH/P25 (Ye et al. [249]). **C–f** Photo-catalytic CO_2_RR of the TiO_2_/Ti_3_C_2_ (TT-*x*) samples and P25 for the CH_4_ formation (**c**) and FE-SEM images of TT550 (**d**, **e**) and TT650 (**f**) (Low et al. [250]). **g**, **h** Photo-induced e^−^ migration method at Ti_3_C_2_/Bi_2_WO_6_ hetero-interface (**g**) and photo-catalytic activity of Ti_3_C_2_/Bi_2_WO_6_ through diverse mass ratios of Ti_3_C_2_ to Bi_2_WO_6_ (0%, 0.5%, 1%, 2%, and 5%) (**h**). Adapted with permission from Ref. [251]

Research certainly showed benefits of 2DMs photo-catalyst for CO_2_ photo-reduction. Adsorption of CO_2_ is a significant condition for CO_2_ photo-reduction, which considerably influences e^−^ transfer method. Here, it is attractive to find suitable approach to boost adsorption site for CO_2_ adsorption and generate strong contact to make possible e^−^s transfer for efficient activation. Research concluded that generating surface defect sites is possibly another way [97]. Although with controlled synthesis, ZnAl-LDH ultra-thin NSs with thicknesses of 2.7 and 4.1 nm and bulk ZnAl-LDH with almost 210 nm thicknesses can be synthesized that are known as ZnAl^−1^, ZnAl^−2^, and ZnAl^−3^, correspondingly. While bulk ZnAl-LDH thickness is reduced to ultra-thin configuration, density of V_o_ defects is increased, so reduces coordination number of nearby Zn-ion and initiates several coordinative unsaturated Zn-ions. So, Zn^+^–V_o_-complexes are built in ZnAl-LDH NSs as supported through XAFS, ESR, and PAS spectra calculations. In CO_2_ photo-reduction route, synthesized Zn^+^–V_o_ complexes can function as entrapping positions to encourage CO_2_ adsorption. Simultaneously, EIS and DFT simulation showed that Zn^+^–V_o_ complexes can provide e^−^s entrapping sites to improve charge separation effectiveness and make easy e^−^-transfer to CO_2_. Therefore, defect-rich ZnAl-LDH NSs showed great photo-catalytic activity for conversion of CO_2_ into CO through a 2e^−^ method, with 7.6 µmol g^−1^ h^−1^ conversion efficiency for ZnAl-1. Further improved CO_2_ conversion competency, selectivity, and developed stability obtained by Xie et al. [154] tuned V_v_ into a single-unit-cell o-BiVO_4_ and then it is used as a photo-catalyst for CO_2_-reduction. Using cetyltrimethylammonium bromide, a lamellar hybrid half way plan was used for the formation of o-BiVO_4_ layers with single-unit-cell thickness. Regulating reaction time and temperature, V_v_-rich and V_v_-poor o-BiVO_4_ atomic layers were formed with [001] direction. Atomically thick o-BiVO_4_ NSs showed AFM and TEM results. As proved through PAS and XRF, V_v_ with discrete levels was formed on V_v_-poor o-BiVO_4_ and Vv-rich o-BiVO_4_ NSs surface. V_v_ showed a significant function in photo-catalytic method for CO_2_-reduction. Firstly, V_v_ created a new defect concentration in BG and enhanced h^+^s level near the Fermi level. Therefore, light harvesting of V_v_-rich o-BiVO_4_ was improved and electronic conductance was better. The V_v_-rich o-BiVO_4_ showed an increased CO_2_-adsorption capacitance and stronger surface hydrophilicity comparative to V_v_-poor o-BiVO_4_; this was certainly useful to CO_2_ reduction process. Finally, V_v_ improved charge separation and efficiently boosted carrier lifetime that permits more e^−^s to engage in CO_2_ photo-reduction. To get benefit from already discussed benefits, V_v_-rich o-BiVO_4_ showed improved conversion effectiveness with methanol production rate of 398.3 µmol g^−1^ h^−1^. In photo-reduction method, just small concentration of H_2_ and trace amount of ethanol can be detected, signifying highly suitable method for product selection. Furthermore, the V_v_-rich o-BiVO_4_ can undergo continuous photo-reduction reaction up to 96 h, lacking any clear decrease in the photo-catalytic efficiency. The above such outcomes show that the 2D photo-catalysts are efficient choice for getting highly efficient CO_2_ reduction. In case of pristine MXene, somehow the consideration of surface terminations (–OH and –O functional group) was very important to clarify reaction steps for CO_2_RR. In case of the Cr_3_C_2_ and Mo_3_C_2_ MXene, with no surface termination groups, energy input of 1.05 and 1.31 eV was essential for transfer of CO_2_ to CH_4_ (Fig. 20a). While Mo_3_C_2_ surface was terminated with OH or O, energy was further decreased to 0.35 and 0.54 eV, correspondingly. Therefore, OH or O-terminated MXene was certainly made easy CO_2_ conversion in comparison with un-functionalized MXene. Moreover, photo-catalytic reduction of CO_2_ at V_o_ on Ti_2_CO_2_, Ti_3_C_2_O_2_, and V_2_CO_2_ was studied theoretically by the first-principles DFT simulations [247]. These results showed that Ti_2_CO_2_ needs the minimum energy of reaction method and therefore proves good CO_2_RR activity. The CO_2_ was decreased to subsequent four compounds: HCHO, CH_3_OH, CH_4_, and HCOOH. Obviously, whole energy barrier for CO_2_ hydrogenation into HCOOH at O_v_ in Ti_2_CO_2_ SL was very good with just 0.53 eV compared with other reduction products that needed superior kinetics. For example, O_v_ on O-terminated MXene was active site in CO_2_RR for high HCOOH selectivity; therefore, MXene NSs can be used for broad range of applications.

Ye et al. [252] joined surface-alkalinized Ti_3_C_2_ MXene as co-catalysts with marketable P25 through a simple mechanical mixing technique for important improvement in photo-catalytic CO_2_RR. Following surface alkalinization, 5 wt% Ti_3_C_2_(OH)_2_-doped P25 (5TC-OH/P25) reveals obvious support in CH_4_ release as compared to un-modified 5TC/P25 (Fig. 20b). The DFT study showed that adsorption energy of CO_2_ on TC-F (F-termination) was superior to CO_2_ on TC-OH (OH-termination). Thus, CO_2_ molecules were easily adsorbed at TC-OH surface, leading to the synthesis of activated CO_3_^2−^. So, encouraging charge separation, extraordinary electrical conductance, sufficient CO_2_ adsorption, and activation sites on alkalinized MXene were main things contributed to photo-catalytic improvement. These clearly showed the major job of surface alkalinization of MXene, as a valuable metal-free co-catalyst for synthetic photo-synthesis.

Similarly, Low et al. [253] studied in situ formed TiO_2_ NPs on conductive Ti_3_C_2_, to form TiO_2_/Ti_3_C_2_ hybrids (TT-*x*, where *x* shows the calcination temperature) through thermal annealing for CH_4_ production from CO_2_RR (Fig. 20c). After –F functional groups removal at elevated temperature, it brings oxidation of Ti_3_C_2_ tuned with –O functional groups. The TT550 and TT650 morphology was clearly different from Ti_3_C_2_ but analogous to rice crust (Fig. 20d–f). High conductivity of Ti_3_C_2_ promotes e^−^-transfer from TiO_2_ and exclusive rice crust analogue morphology with a large active site density, considerably push photo-catalytic activity. Referring to precede work which is discussed already [252], it was hard to straight evaluate both TiO_2_/Ti_3_C_2_T_*x*_ photo-catalytic systems due to different synthesis methods, unlike TiO_2_ phase, discrete difference in morphology, and diverse MXene surface modification. Therefore, it is sensible to embrace AQY for CO_2_RR as a controlled tool to describe different future experiment conditions. In recent times, same group of researchers has formed ultra-thin 2D/2D Ti_3_C_2_/Bi_2_WO_6_ hetero-junction hybrid nano-composites (Fig. 20g) [254]. Due to electronic coupling and intense physical effects, 2D/2D hetero-junction noticeably improves transfer and partition of photo-induced charge carriers to reduce charge recombination. 2 wt% Ti_3_C_2_-modified Bi_2_WO_6_ NSs (NSs) (TB_2_) records the highest CH_4_ release rate than other stoichiometry (Fig. 20h). Moreover, large interfacial contact surfaces of intimate 2D/2D hetero-junction donate more quick charge mobility in comparison with 0D/2D and 1D/2D hetero-junctions due to a decrease in charge transfer path. So, such stimulating investigation on parallel 2D/2D hetero-interfaces generates novel potential in material science for layered hetero-junctions design in electrocatalysis and photo-catalysis for energy conversion.

### Nitrogen (N_2_) Fixation

In comparison with CO_2_RR, the photo-catalytic N_2_-fixation is still more demanding as dissociation enthalpy of N_2_ molecule triple bond (962 kJ mol^−1^). Normally, catalyst conversion for N_2_ is enormously harsh as N_2_ just weakly binds with solid-state catalysts and reaction entails high-energy intermediates. So, it is very much required to do an appropriate structure to support conversion competency of N_2_ to NH_3_. E0 to make the N^2−^ is as high as − 4.2 V versus NHE via ${\text{N}}_{2} + {\text{e}}^{ - } \to {\text{N}}^{2 - }$ method, whereas proton-coupled e^−^ transfer reaction ${\text{N}}_{2} + {\text{H}}^{ + } + {\text{e}}^{ - } \to {\text{N}}_{2} {\text{H}}$ has more available *E*_0_ of − 3.2 V versus NHE [255]. Proton-supported exchange method might evade production of high-energy intermediates and therefore reduce thermodynamic kinetics for NH_3_ formation. Current investigations showed that 2DMs are a talented applicant to get an efficient photo-catalytic N_2_ fixation [249]. Zhang and co-workers [250] studied that V_o_ formation in BiOBr NSs can efficiently boost N_2_ fixation reaction. Theoretical study showed that N_2_ adsorption onto the V_o_ through an end on arrangement of adsorbed N_2_ triple bond can be extended from 1.078 Å in original N_2_ to 1.133 Å, signifying efficient N_2_ activation. Generated e^−^s in BiOBr can simply inject into N_2_ *π* antibonding orbitals. Accessibility of localized *π*-back donating e^−^s in V_o_ might efficiently adsorb N_2_ to generate activation. So, N_2_ reduction to NH_3_ catalyzed through the BiOBr with V_o_ needs a very low reaction kinetic and a higher photo-catalytic performance can be obtained. N_2_ conversion rate after vis-light and UV–Vis-light irradiation is 104.2 and 223.3 µmol g^−1^ h^−1^, respectively, after without h^+^ scavenger or co-catalyst. Stimulated through V_o_-motivated N_2_ activation, MoS_2_ NSs with S-vacancy were formed and further utilized to N_2_ fixation. The MoS_2_ NSs showed NH_3_ formation rate almost 325 µmol g^−1^ with 10-h measurement after simulated solar light irradiation. Although marketable bulk MoS_2_ cannot give any photo-catalytic performance for NH_3_ synthesis under similar test condition, it illustrates exclusive benefit of MoS_2_ NSs for N_2_-reduction. According to Mott–Schottky spectra, MoS_2_ NSs and bulk MoS_2_ samples in CB positions were anticipated to be − 0.35 and − 0.24 V, correspondingly, that were positioned below thermodynamic reduction potentials of N_2_ through one or two-e^−^ transfer method. So, it was presumed that N_2_ reduction with MoS_2_ NSs was multi-electron coupled proton transfer method. Due to n-type SC essence, there subsists many free e^−^s in MoS_2_ NSs and such free e^−^s can pair with photo-generated excitons to form charge excitons (trions) that were mostly located around Mo-sites. Produced trions have manifold e^−^s in one bound state that was useful to multi-e^−^ migration reactions. While N_2_ is confined through S-vacancies, it was bounded via three Mo atoms with trions after irradiation. N_2_ activated after e^−^s donation from bonding orbitals and accepting e^−^s to its antibonding orbitals results in a trion-supported six-electron reduction method. The significant photo-catalytic N_2_ fixation resides in building adsorption site in N_2_ molecule, e^−^-rich systems for e^−^ donation, engineering band configuration through enough E_0_ and coupling protons to decrease energy condition of intermediates. So, theoretical and experimental study showed that novel breakthrough on MXene-based composites for CO_2_RR and N_2_RR will appear in the frontline of technology and science, transferring them in future real energy applications.

### Organic Synthesis

**Fig. 21:**
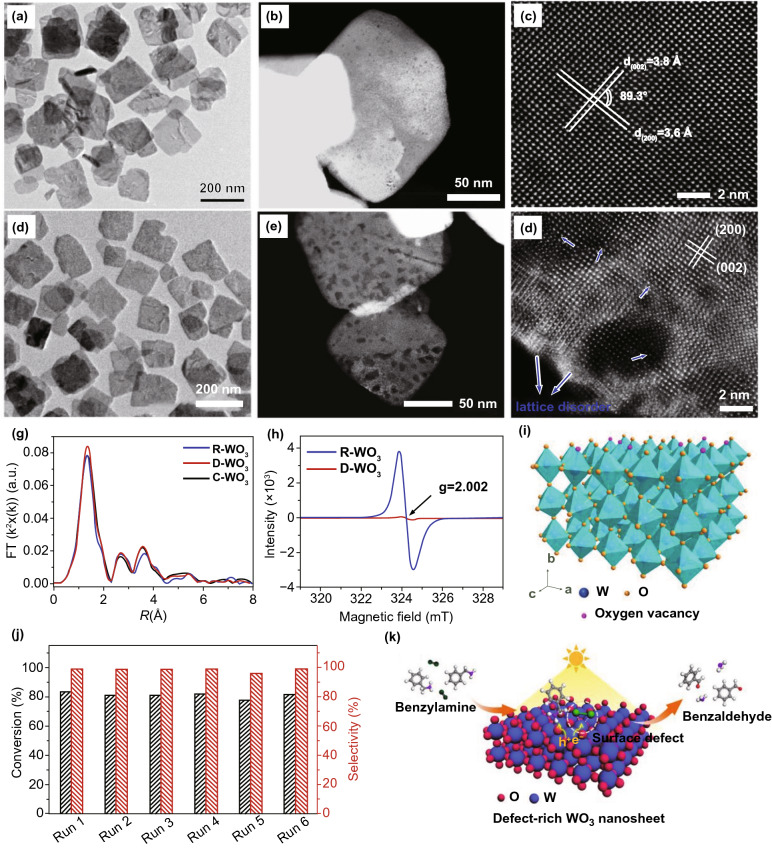
**a**–**f** Morphological study. **g** Fourier transforms W L3-edge EXAFS spectra with respect to commercial WO_3_. **h** ESR spectra at room temperature. **i** Scheme showing locations of V_o_ in WO_3_ lattice. **j** Cyclic analysis for defect-rich WO_3_ in catalytic aerobic coupling of benzylamine after irradiation with *λ* > 400 nm at 298 K. **k** Scheme shows total light-driven catalytic reaction route. Adapted with permission from Ref. [257]

Idea of decreasing energy expenditure for chemically developed, solar light-driven chemical transformation through assist of the SCs holds huge view. Under irradiation, SCs can use solar light to make an exciton or hot carriers that can stimulate chemical reactions at surface of catalyst. Effectiveness and selectivity of the photo-catalytic conversion are still in adequate at large scale. Weak interaction of O_2_-molecule with photo-catalysts surfaces, particularly a defect-free surface, is a serious matter for poor effectiveness of photo-catalytic organic formations. Reaction involves O_2_, which needs efficient interfacial e^−^-transfer, either directly as donor or indirectly as e^−^-acceptor [256]. Additional significant subject is poor selectivity that might be obtained from photo-generated h^+^s. Generally, generated h^+^s has strong oxidizing capacity and is accountable for nonselective over-oxidation. In recent times, the 2D photo-catalysts displayed a huge promise for selective organic transformation using mild conditions. For example, Xiong et al. [257] formed V_o_ into ultra-thin WO_3_ NSs to make O_2_-molecules active and activate organic conversion. Defect-rich (DR) WO_3_ NSs were formed through calcination of the first synthesized WO_3_·H_2_O NSs in N_2_-environment, at 673 K, whereas defect-deficient (DD) WO_3_ NSs were synthesized via calcination in atmospheric condition. From aberration-corrected HAADF-STEM, it is clear that DD WO_3_ shows comparatively smooth and flat surface. Simultaneously, continuous and controlled lattice fringe was experienced from atomic resolution HAADF-STEM image (Fig. 21). Such outcomes conclusively proposed the defects deficiency in such DD WO_3_ sample. In DR WO_3_, many small pits were formed, with small lattice disorder, and dislocation appears in NSs that vigorously emphasize the presence of different defects.

To further reveal the presence of V_o_, synchrotron radiation-related XAFS spectroscopy was performed. The DR WO_3_ showed a clear diverse confined atomic configuration compared to DD WO_3_, where W-atom coordination numbers decrease from 6 to 5.4 for DD WO_3_ and DR WO_3_, showing local deficiency of O_2_-atom. ESR investigation was also used to calculate WO_3_ samples. Noticeably, at *g* = 2.002 a symmetric ESR signal was experimented for the DR WO_3_, showing e^−^-entrapping at V_o_. Joint STEM, XAFS, and ESR findings suggested that subsistence of V_o_ at precise sites induces few lattice distortion and displacement. After getting benefits from DR configuration, O_2_ was chemisorbed at V_o_ of defect-rich WO_3_ NSs through end on arrangement and associated with e-s transfer from coordinative unsaturated site to O_2_. So, O_2_ was efficiently activated to O^2·−^ species over DR WO_3_, and it was transformed from amines to respective imines with 6 times enhanced kinetic rate compared to DD WO_3_. As reaction time increased to 8 h as conversion ratio of benzylamine was greater compared to 80% with an extremely high selectivity, no clear decline was observed within 6 times of catalytic cycles. Other than O_2_-activation, hydrophobicity was another significant feature to affect photo-catalytic organic conversion method. Li and co-workers [258] studied colloidal formation approach information of BiOCl NSs. Through BiCl_3_ hydrolysis in octa-decylene solution, supported by in situ preparation of H_2_O via reaction in oleylamine and oleate solution, single-crystalline BiOCl colloidal NSs (BiOCl C-UTNSs) were obtained with almost 3.7 nm thickness. For comparison, BiOCl NSs were also formed through hydrothermal way, known as BiOCl H-UT-NSs. Surface H_2_O contact angle (CA) measurement was utilized to establish wettability of as-synthesized BiOCl NSs. BiOCl C-UT-NSs showed a H_2_O CA of 116.3°, which is hydrophobic. It occurs from detail that organic ligands have capped on BiOCl C-UT-NSs surface during colloidal formation. Conversely, BiOCl H-UT-NSs exhibited a H_2_O CA of 0°, showing the super-hydrophilic nature of synthesized BiOCl H-UT-NSs. Huge difference in BiOCl C-UT-NSs and BiOCl H-UT-NSs may bring about important effect for photo-catalytic organic conversion method. Moreover, there subsist plentiful V_o_ on BiOCl C-UT-NSs, ensuing strong light absorption in vis-light range. To get benefit from hydrophobic character and enhanced light harvesting capability, BiOCl C-UT-NSs displayed greatly enhanced photo-catalytic activity for conversion of N-t-butylbenzylamine to N-t-butyl-benzylamine. The 78% conversion ratio was obtained from BiOCl C-UT-NSs, whereas BiOCl H-UT-NSs have only displayed about 15% conversion rate, from Xe lamp irradiation for 1 h. Furthermore, BiOCl C-UT-NS sample was more utilized for conversion of secondary amines to respective imines along increased conversion selectivity as well as efficiency. The 2D photo-catalysts were verified to be talented choice for the photo-catalytic organic formation, and such approach can broaden perceptive of organic conversion method and is favorable to establish further proficient organic transformation systems.

### Removal of Pollutants

**Fig. 22:**
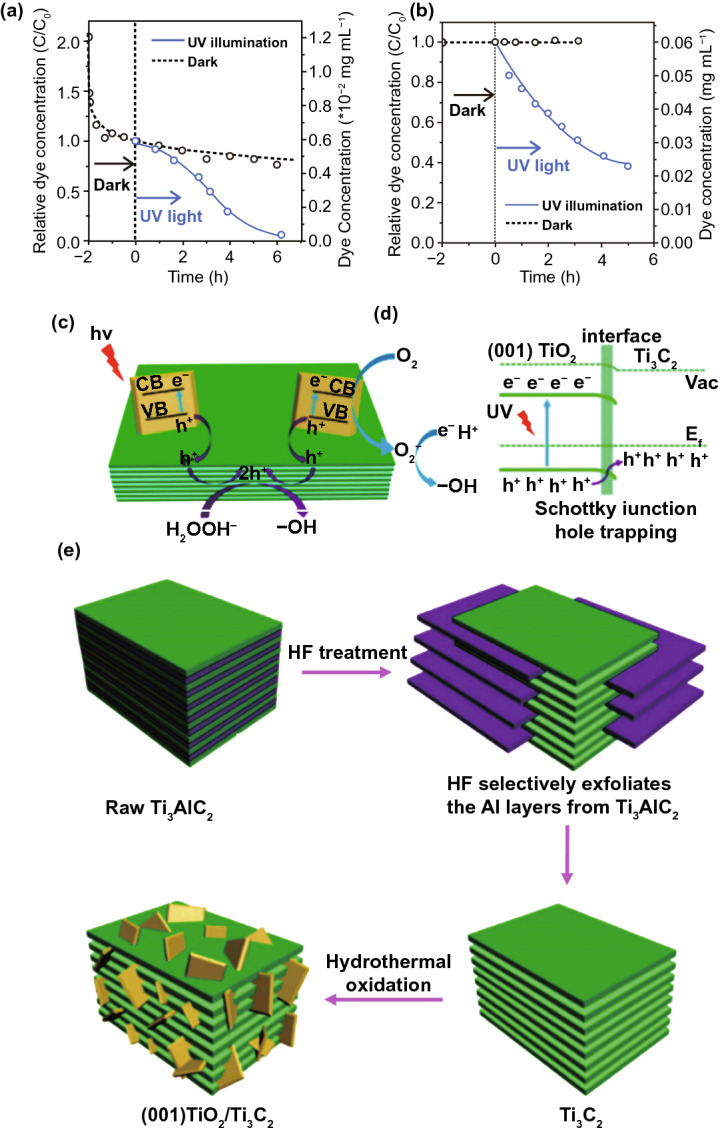
Photo-catalytic pollutant degradation utilizing MXenes and their hybrid nano-composites. **a**, **b** Time-dependent MB respective level (**a**) and AB80 (**b**) in Ti_3_C_2_T_*x*_. Adapted with permission from Ref. [135]). **c–e** Preparation of (001)TiO_2_/Ti_3_C_2_ nano-hybrids (**c**), charge transfer process (**d**), B.G. (**e**) of (001)TiO_2_/Ti_3_C_2_ after light irradiation. Adapted with permission from Ref. [259]

In the progress of financial and industrialization, environmental pollution is the main problem that threatens the public health. The photo-catalysis was considered as an efficient and financially viable technology to handle elimination of environmental pollutions. Due to exclusive advantages such as better adsorption capability of pollutants and strong light harvesting capacitance, 2DMs-based photo-catalysts showed a great hope for removal of pollutants. For instance, Xia and co-workers [140] formed ultra-thin Bi_4_O_5_Br_2_ NSs through reactive ionic liquid supported via solvothermal method in combination with pH adjustment. In ionic liquids, long carbon chain served as a capping reagent which controls the crystal growth along c-axis. Simultaneously, reaction condition pH was adjusted to 11 that supplied OH^−^ to substitute Br- to execute de-halogenation in fabrication route of Bi_4_O_5_Br_2_. So, both thickness and component-engineered Bi_4_O_5_Br_2_ materials were formed and utilized in photo-catalytic degradation of an antibiotic CIP and tetracycline (TC) after irradiation with vis-light for 120 min. After irradiation, 75% of CIP is photo-degraded through Bi_4_O_5_Br_2_, whereas BiOBr degradation rate was 51.4%. Furthermore, Bi_4_O_5_Br_2_ NSs showed 77.8% degradation rate for TC within 60-min irradiation, which is very high compared to BiOBr with just 31.7%. Changeable energy band configuration of Bi_4_O_5_Br_2_ was verified to consider the increased photo-catalytic activity. More negative CB position of Bi_4_O_5_Br_2_ will make easy development of more active O_2_^·−^ species. Upshifting of CB position and broad VB will be advantageous to improve charge separation effectiveness. Thus, obtained Bi_4_O_5_Br_2_ NSs displayed greater performance toward pollutant removal. To further enhance photo-catalytic performance in pollutant degradation, creating surface defects might be another approach. Therefore, Xie and co-workers [119] studied that while BiOCl thickness is reduced from 30 to 2.7 nm, in BiOCl defect type will vary from isolate defects VBi″′ to triple vacancy-related VBi″′VO^··^VBi″′, as confirmed with PAS. Through desirable quality from triple vacancy-associated VBi″′VO^··^VBi″′ along four negative charges, BiOCl NSs were additionally negatively charged as comparative to BiOCl nano-plates. As RhB was positively charged, the further negatively charged ultra-thin BiOCl NSs promoted RhB adsorption on BiOCl NSs’ surface. Moreover, the presence of vacancies can enhance light absorption and speed up separation of charges. As a result, ultra-thin BiOCl NSs exhibited a great solar photo-catalytic activity for RhB removal. MXene shows outstanding performance in photo-catalytic degradation of organic pollutants. Mashtalir et al. [135] utilized Ti_3_C_2_T_*x*_ to degrade MB (a cationic dye) and acid blue 80 (AB80) (an anionic dye) (Fig. 22a, b). The MB and AB80 degradation was augmented via UV irradiation. In dark, MB level reduces due to negatively charged adsorption at Ti_3_C_2_T_*x*_ surfaces with MB. After UV irradiation, a substantial reduction in MB and AB80 concentration, with 81% and 62%, respectively, was experienced in existence of suspended Ti_3_C_2_T_*x*_. It is observed that over longtime period, Ti_3_C_2_T_*x*_ oxidation to form TiO_2_ in dissolved O_2_ presence was obvious that merits wide ranging research in this field. Similar to G-TiO_2_ nano-composites, it was imagined that Ti_3_C_2_T_*x*_-supported TiO_2_ may function as a possible catalyst that further promotes progress of this direction. While continuing such investigation for the design of hetero-junction interfaces, Peng et al. [259] studied a composite of Ti_3_C_2_ and {001} facets-exposed TiO_2_ through hydrothermal incomplete oxidation of Ti_3_C_2_ (Fig. 22c–e).

**Fig. 23:**
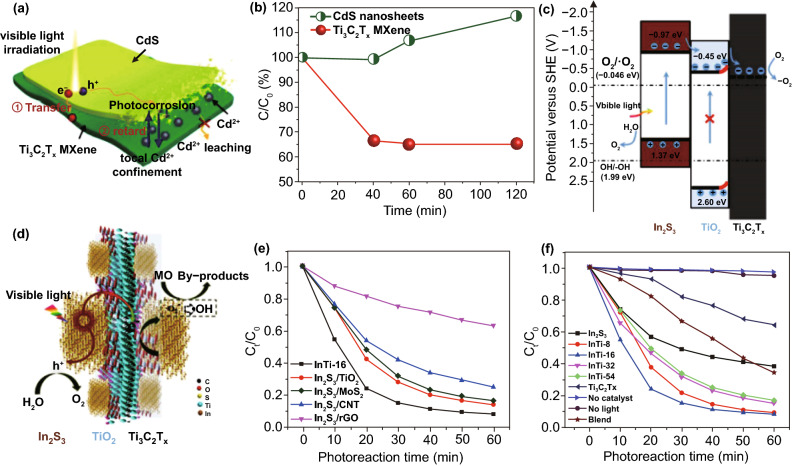
**a** Scheme of CdS/Ti_3_C_2_T_*x*_ NSs to improved photo-activity and photo-stability. **b** Adsorption of Cd^2+^ over CdS NSs and Ti_3_C_2_T_*x*_ MXene in dark. Adapted with permission from Ref. [261]. **c** Charge migration. **d** Separation and reaction mechanism for MO degradation in In_2_S_3_/anatase TiO_2_@metallic Ti_3_C_2_T_*x*_ (InTi) system after vis-light and photo-catalytic degradation of MO over other In_2_S_3_-based binary hybrids. **e** In_2_S_3_, In Ti hybrids, and Ti_3_C_2_T_*x*_. **f** In Ti-*x* indicates mass of Ti_3_C_2_T_*x*_ included during preparation (*x* = 8, 16, 32, and 54 mg). Adapted with permission from Ref. [262]

In photo-catalytic reaction, TiO_2_ through preferential {001} facets creates e^−^s and h^+^s through UV light illumination and, afterward, Ti_3_C_2_ develops a Schottky junction with {001}-face n-type TiO_2_. It hinders recombination of e^−^s with h^+^s due to SB. Enriched e^−^s on TiO_2_ (001) might interact with dissolved O_2_ to generate superoxide radical anions (O^2−^) that further respond to H^+^ and e^−^ to produce extremely reactive hydroxyl (OH) radicals, which increase photo-degradation. It is well recognized that photo-catalytic activity of TiO_2_ relies not only on particle shape but also on its exposed facets. It seems that {001} surface offers oxidation sites in photo-catalytic method, while {101} facets proceed as reductive sites. Therefore, TiO_2_ facet tuning using MXene has basic significance to systematically untangle underlying photo-catalytic system [241]. In addition, TiO_2_ metal sulfides are also utilized to join with Ti_3_C_2_T_*x*_. Xie et al. [260] formed a 2D in-plane CdS/Ti_3_C_2_T_*x*_ onto sheet hetero-structures via electrostatic self-assembly method (Fig. 23a). In such catalytic system, the Ti_3_C_2_T_*x*_ Janus co-catalysts not just act as an e^−^ mediator to augment e^−^s extraction from CdS but also restrain h^+^-mediated photo-corrosion of CdS. Assigning Ti_3_C_2_T_*x*_ small Fermi level than CdS CB, photo-e^−^ lifetime of CdS/0.5% Ti_3_C_2_T_*x*_ was longer as compared to bare CdS.

Moreover, Ti_3_C_2_T_*x*_ can absorb Cd^2+^ ions that were produced during photo-catalysis (Fig. 23b) and, as a result, avoid Cd^2+^-ions dissolution in H_2_O to enhance photo-stability of CdS. For instance, double-gain strategy offers a conceptual idea to evade instability as well as photo-corrosion of CdS. Other than MXene-based binary hybrid nano-composites, prosperous study into ternary hetero-structures has become a mainstream in photo-catalysis area. Wang et al. [262] (2018) formed a new quasi-core–shell In_2_S_3_/anatase TiO_2_@metallic Ti_3_C_2_T_*x*_ MXene hetero-structure hybrids through in situ hydrothermal technique for degradation improvement in methyl orange (MO). Fascinatingly, enhanced photo-activity of ternary nano-architectures was credited to numerous associated factors, such as well-designed type II band arrangement and noble metal-free-based Schottky junction with promising charge migrating channels (Fig. 23c, d). Specially, such occurrence originates from synergistic contribution in between vis-light-responsive In_2_S_3_, upward band bending in TiO_2_, and amazing electrical Ti_3_C_2_T_*x*_ conductance. For example, optimal photo-catalyst in the presence of Ti_3_C_2_T_*x*_ content of 16 mg (InTi-16) evidents the highest photo-degradation competency for MO elimination, as well as more significantly, it exceeds other In_2_S_3_-based binary NSs (Fig. 23e, f). Progressing via this analysis, it can be elucidated that key to ameliorate dyes photo-catalytic degradation is to effectively separate photo-induced (e^−^–h^+^)-pairs to hold back charge recombination. Consequently, efficiently tuned vigorous hetero-structure system with distinct quality (e.g., intimate contacted hetero-interfaces, use broad solar spectrum to imitate natural sunlight, superior conductance, constructive charge transfer, and separation) is an urgent demand for boosting photo-catalytic degradation. Incidentally, extensive research in chemistry, materials arrangement, and optimization is the main requirement. In turn, it will open new prospects for separating charge carrier dynamics in synergistically speeded up photo-activity in practical photo-catalytic applications.

### Hydrogen Peroxide (H_2_O_2_) Production

Since the first synthesis of H_2_O_2_ by Thenard (1818) as a result of barium peroxide reaction with nitric acid [263], H_2_O_2_ has gotten rising consideration in past 200 years due to which it was listed among the 100 most significant chemicals in world [264]. The anthraquinone oxidation (AQ) is mostly developed for H_2_O_2_ manufacture on industry level that is presently caused about 95% of total H_2_O_2_ formation. Normally, AQ method generally contains four steps [265]:

1. Hydrogenation of AQ in organic solvent by means of Ni/Pd catalyst.
2. Oxidation of hydrogenized AQ (HAQ) in air or O_2_-enriched atmosphere with the help of catalysts.
3. Removal of H_2_O_2_ and recycling HAQ to AQ.
4. Refinement and concentration of H_2_O_2_.

The multi-step oxidation and hydrogenation response need an elevated applied energy. On the other hand, AO method is not environmentally benevolent, since large quantity of waste H_2_O (for example, 2-ethyl-anthraquinone, tri-octyl phosphate, tert-butyl urea, and K_2_CO_3_ lye), exhaust gas (mesitylene isomers), and solid waste (activated alumina) was formed. It is well known that H_2_O_2_ is a very competent and ecological oxidant. It has maximum content of active O_2_ (47.1% w/w), and no noxious side products are formed in its reactions, apart from H_2_O and O_2_. Due to these qualities, H_2_O_2_ has broadly applied in organic synthesis, [266] waste H_2_O management, disinfection [267], and paper industry [268]. Nowadays, H_2_O_2_ is studied in energy field as both oxidant and reductant in innovative fuel cell [269]. The results illustrated that theoretical output potential of the H_2_O_2_ fuel cell was 1.09 V that is analogous to traditional H_2_-fuel cell (1.23 V). The H_2_O_2_ has established rising concentration as it is not only a mild and environment friendly oxidant but also a talented novel liquid fuel. Formation of H_2_O_2_ by photo-catalysis is green, sustainable, and potential method, in view of its utilization in H_2_O and O_2_ as source materials and solar light as energy. Other advantage of H_2_O_2_ as compared to H_2_ is that it is completely soluble in H_2_O and simply transportable that paves it as energy carrier perfectly.

Synthetic photo-synthesis is a photochemical method to renovate sustainable resources into clean fuels and chemicals (for example, H_2_O_2_) through sunlight which was expected to solve rising energy requirements. Photo-catalytic-based H_2_O_2_ formation is a talented approach to improve energy requirements, as H_2_O_2_ is significant liquid chemical and fuel. However, subsequent dilemma strictly limits the growth of this method:

1. Less selectivity.
2. Less stability (usually > 5 short-time cycles).
3. Quick charge recombination.
4. Support of hole scavengers.
5. Requirement of O_2_ saturation.

TiO_2_ is broadly considered as a photo-catalyst because of its crystal stability, optical, physical and electrical properties as well as biocompatibility [17, 18]. H_2_O_2_ manufactured by TiO_2_ photo-catalytic attracted a great concentration [19]. The TiO_2_ CB (− 0.19 V vs. NHE, pH 0) bottom is more negative than 2e^−^ reduction of O_2_ (0.68 V), which promotes reduction reactions for H_2_O_2_ fabrication, but pristine TiO_2_ has few disadvantages for example, poor light absorption due to its large BG. Mainly, quantity of H_2_O_2_ generation is much low (< 0.2 mM) over pristine TiO_2_-based photo-catalyst that is away from reasonable level. This may be after the formation of H_2_O_2_; it straight away reacts with surface Ti–OH groups and forms Ti–OOH complexes. Secondly, Ti-OOH complexes dissociated to Ti–OH and OH^−^ through e^−^ reduction as:

$${\text{Ti}}-{\text{OOH}} + {\text{H}}^{ + } + {\text{e}}^{ - } \to {\text{Ti}}-{\text{OH}} + {\text{OH}}^{ - }.$$

Numerous surface alteration approaches were applied to boost photo-catalytic fabrication of H_2_O_2_ in TiO_2_-based photo-catalytic method, for example surface fluorination and surface complexation. The g-C_3_N_4_ is analog to graphite, and metal-free polymer n-type SCs have stacked 2D configuration of tri-s-triazine linked through tertiary amines [38]. In consequence of its exceptional structural, electrical, optical, and physicochemical properties, g-C_3_N_4_ is familiar as a novel class of multipurpose materials for catalytic, electronic, and energy uses [39]. Wang and co-authors (2009) first revealed photo-catalytic properties of g-C_3_N_4_ on H_2_ and O_2_ evolution [40], and g-C_3_N_4_-based photo-catalysts have attracted boosting interest worldwide [41]. BG of g-C_3_N_4_ is ~ 2.7 eV which is similar to optical wavelength of ~ 460 nm, that makes it a possible vis-light-active photo-catalyst. Additionally, g-C_3_N_4_ has photo-catalytic capability for H_2_O reduction and oxidation because of its suitable BGs [42]. Hypothetically, g-C_3_N_4_ is a good photo-catalyst applicant for H_2_O_2_ formation [10] as its CB position (− 1.3 V vs. NHE) is correctly located to make possible O_2_ reduction (− 0.28 V vs. NHE), while VB potential (1.4 V vs. NHE) is smaller compared to metal oxides that can efficiently avoid oxidative disintegration of H_2_O_2_. As such, g-C_3_N_4_ rapidly becomes attractive in the field of photo-catalysis H_2_O_2_ formation [43]. However, photo-catalytic H_2_O_2_ formation activity of g-C_3_N_4_ is still limited via low effectiveness in consequence of some adverse parameters, generally including lower surface area, inadequate vis-light harvesting, and quick recombination of photo-induced (e^−^–h^+^)-pairs. Protocols, for instance engineering structures, controlled defects, loaded noble metal nanoparticles, doping elementals, and heterogenization, were later applied to improve g-C_3_N_4_-based photo-catalytic H_2_O_2_ formation.

## 2D/2D Hetero-junctions for Catalysis

The description of hetero-junction, initially developed from SC–SC (S–S) junction, now has been elaborated to scope metal–SC (M-S) junction and still nontypical hetero-structures of SCs and ionic conductors [136]. Mostly, edge coupling of two components in a hetero-junction could make band arrangement or repairing contact after Fermi levels equilibration (or work functions) at interface following Anderson’s rule or Schottky–Mott rule for S–S or M–S junctions, correspondingly. There is an agreement in previous work that re-localization of charge carriers at hetero-junctions edge may make easy catalytic activity of as-fabricated materials or devices.

### Photo-catalytic H_2_ Production

Since 1972, Fujishima and Honda [37] discovered H_2_O splitting at TiO_2_ electrode under UV light irradiation; at the same time, numerous efforts were dedicated to photo-catalytic H_2_-production [136]. The photo-catalytic is a procedure that produces H_2_ (and O_2_) using reduced or oxidized adsorbed H_2_O through photo-generated e^−^s and h^+^s at SC catalysts’ surface. Due to quick recombination of photo-generated (e^−^–h^+^) in catalysts, for H_2_ production effectiveness was still far from prerequisite practical applications. Therefore, 2D/2D layered composite photo-catalysts with suitably formed hetero-junctions are moderately promising for increasing H_2_-production effectiveness via supporting separation of photo-generated (e^−^–h^+^) [270]. In comparison with UV light with small percentage of the solar emission, vis-light (almost 50% of solar radiation) determined that the photo-catalysts are more capable for high proficient sunlight utilization and photo-catalytic activity. Zhang et al. [271] developed a type of “sheet on sheet” hierarchical hetero-structure for vis-light-based photo-catalytic H_2_-production via in situ development of ZnIn_2_S_4_ 2D-NSs on sheetlike g-C_3_N_4_ surfaces. g-C_3_N_4_ was one of the most talented photo-catalysts because of its good stability, non-toxicity, exceptional electronic configuration, and cost efficiency. Its photo-catalytic effectiveness is restricted through poor light harvesting effectiveness and quick recombination of photo-generated charge carriers. Combining vis-active ZnIn_2_S_4_ 2D-NSs, the above shortcomings were overcome based on hetero-junction contact interface that can persuade proficient interfacial transfer of photo-generated (e^−^–h^+^) from g-C_3_N_4_ to ZnIn_2_S_4_ and delayed charge recombination depending on measurement findings of surface photo-voltage and PL of ZnIn_2_S_4_/g-C_3_N_4_ hetero-structures. Both suppressed charge recombination on g-C_3_N_4_ NSs and enhanced photo-generated charge carriers in ZnIn_2_S_4_ NSs give amazing improvement on photo-catalytic activity of ZnIn_2_S_4_/g-C_3_N_4_ hetero-structures for H_2_-production.

Enhanced photo-catalytic activity should also be contributed to increase in surface active sites and extension of light absorption via ZnIn_2_S_4_ NSs combination. The overall photo-catalysts-based H_2_O splitting was also developed for production of O_2_ and H_2_ concurrently, but it is still a major confront [270]. To fulfill redox potential for overall H_2_O splitting, photo-catalysts CBM must more negative as compared to E_0_ of H^+^/H_2_ (0 V vs. normal H_2_ electrode (NHE)) and VBM should be more positive compared to oxidation potential of O_2_/H_2_O (1.23 V). Over-potential related to charge transfer and minimum BG of photo-catalysts for efficient H_2_O splitting is always superior compared to theoretical value (1.23 eV). Therefore, Liao et al. formed 2D MoS_2_/AlN(GaN)-layered hetero-structures as extremely competent vis-light photo-catalysts for overall H_2_O splitting. The H_2_ and O_2_ were formed at opposite surfaces of hetero-structures, because AlN (GaN) and MoS_2_ SLs act as e^−^ donor and e^−^ acceptor in this hetero-junction photo-catalyst, respectively. Pristine MoS_2_ using a direct BG of 1.9 eV was a potential vis-light-driven photo-catalyst and is confirmed not efficient in H_2_O splitting. Group III nitrides (AlN or GaN) SLs with good thermal/chemical stability and highly thermal conductance have demonstrated a good option to manufacture SLs MoS_2_ hetero-structures, to improve photo-catalytic activity. Moreover, there was only around 2% lattice mismatch among h-AlN (GaN) and MoS_2_ SLs, which was major benefit for manufacturing of hetero-structures. The MoS_2_/AlN and MoS_2_/GaN hetero-structures were calculated to be capable photo-catalysts under vis-light irradiation because of proper BGs for H_2_O splitting and good optical absorption [136].

**Fig. 24:**
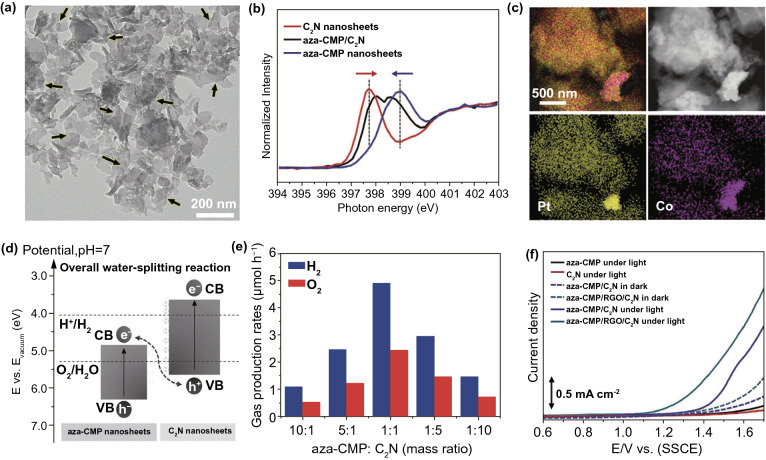
**a** TEM image. **b** NK-edge XANES of aza-CMP, C_2_N, and aza-CMP/C_2_N. **c** HAADF-STEM images and elemental mapping (Pt-labeled C_2_N and Co-labeled aza-CMP). **d** Overall H_2_O splitting activity. **e** Scheme showing aza-CMP/C_2_N hetero-structures electronic band structures. **f** J–V curves of catalysts in dark and under light irradiation. Adapted with permission from Ref. [274]

In recent times, polymers were supposed to be one of the potential alternatives in photo-catalytic overall H_2_O splitting because of its engineering molecular structures [272]. To remove limitation of single-element polymer with inadequate E_0_, 2D/2D polymer hetero-junction photo-catalysts with Z-scheme configuration were designed from imitating two-step excitation way of plants, where light-driven two half reactions of H_2_O splitting and glucose formation are divided spatially [273]. The Z-scheme-based polymer derived from 2DMs was proved to be an effective pathway to get appropriate energy levels for enough reaction kinetics and allow proficient charge transfer in overall H_2_O splitting. Wang et al. [270] synthesized hetero-structures (aza-CMP/C_2_N) consisting of aza-conjugated microporous polymers (CMP) and C_2_N NSs as 2D polymer-related Z-scheme systems for competent photo-catalytic overall H_2_O splitting. The aza-CMP/C_2_N hetero-structures were synthesized via mixing and consequently annealing of CMP and C_2_N NSs. The as-fabricated stacked NSs of aza-CMP/C_2_N with abundant overlapped areas might be exposed via the TEM analysis in Fig. 24a. The XANES spectroscopy, HAADF-STEM image, and elemental mapping were utilized to show interlayer relations as well as homogeneous combination of aza-CMP and C_2_N NSs (Fig. 24b).

Investigation on photo-catalytic activity showed that H_2_ and O_2_ (molar ratio 2:1) were concurrently formed from pristine H_2_O with aza-CMP/C_2_N hetero-structures as photo-catalysts under vis-light (> 420 nm) irradiation, whereas both aza-CMP and C_2_N components are inactive. In addition, aza-CMP/C_2_N hetero-structures with 1:1 mass ratio showed optimum photo-catalytic performance (H_2_ evolution rate: 5.0 mmol h^−1^, solar to H_2_ conversion efficiency: 0.23%, evident QE at 600 nm: 4.3%) (Fig. 24c). Figure 24d presents the energy band position of aza-CMP/C_2_N hetero-structures, which indicates that photo-generated e^−^s in C > B of aza-CMP were quickly recombined with photo-generated h^+^s at VB, of C_2_N at their interface, while other photo-generated e^−^s and h^+^s participated in H_2_O redox reaction. Credit to as-constructed hetero-structures, both charge separation and transfer were assisted in aza-CMP/C_2_N composites (confirmed via outcomes of electrochemical impedance measurements and transient photo-current) and therefore increased photo-catalytic performance.

**Fig. 25:**
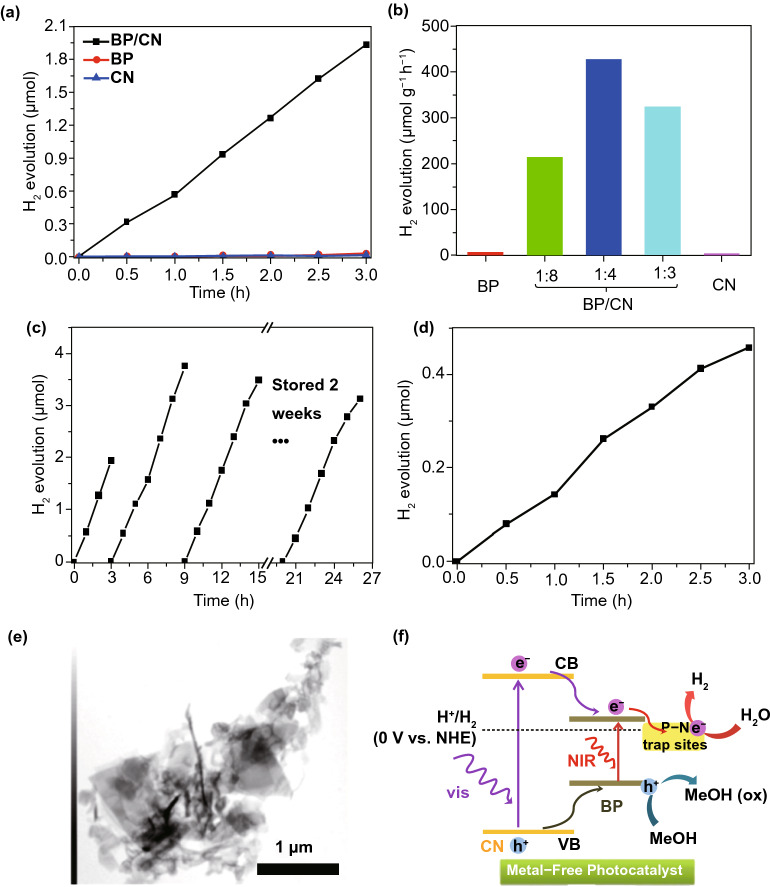
**a** Photo-catalytic H_2_ evolution based on different catalysts with > 420 nm. **b** Effect of BP/CN ratio in BP/CN on photo-catalytic H_2_ production rate under vis-light irradiation for 3 h. **c** Cycle stability test on BP/CN photo-catalytic H_2_ production under vis-light irradiation. **d** Photo-catalytic H_2_ production based on BP/CN with > 780 nm light irradiation. **e** HAADF-STEM image. **f** Scheme for photo-catalytic H_2_ evolution utilizing BP/CN. Adapted with permission from Ref. [275]

As a move toward green chemistry, sequence of metal-free photo-catalysts, e.g., g-C_3_N_4_, BP, boron nitride (BN), and boron carbide (BC), was highly preferred to be studied because of their profusion, inexpensive, and excellent stability. The study of a competent and stable metal-free photo-catalyst with broad spectrum solar absorption for photo-catalytic H_2_-production remains a major issue. Zhu et al. [275] productively formed a 2D layered hetero-structures consisting of BP and graphitic carbon nitride (CN) (BP/CN) as metal-free photo-catalysts in H_2_-evolution for vis to near-IR region for the very first time. Compared to a single element (BP or CN), BP/CN hetero-structures showed considerably enhanced photo-catalytic activity, and H_2_-evolution for 3 h obtained 1.93 µmol, 0.46 µmol under > 420 nm > 780 nm light irradiation, respectively (Fig. 25a, d). It is mostly due to proficient interface charge transfer based on strong interface interaction in CN and BP that restrained recombination and improved the partition of photo-generated (e^−^–h^+^)-pairs (Fig. 25f). After excitation in vis-light, a charge migration from CN to combine BP stimulated through hetero-junction interfacial effect in BP/CN can be established through consequences of time-resolved diffuse reflectance spectroscopy. In NIR excitation case, just BP was excited and extensive excitation duration is obtained from competent electron entrapping via P-N coordinate bond at hetero-junction edge [136].

Jingrun Ran et al. [276] studied metal-free photo-catalysts of metal-free 2D/2D vdWs phosphorene/g-C_3_N_4_ hetero-structure. The synthesized nano-composite showed improved vis-light photo-catalytic H_2_ production activity of ~ 571 µmol h^−1^ g^−1^ in 18 v % lactic acid aqueous solution. This enhanced activity occurs due to intimate electronic coupling at 2D/2D interface, which introduced a new metal-free phosphorene/g-C_3_N_4_ photo-catalyst and formed 2D/2D vdWs hetero-junction for uses in catalysis, electronics, and optoelectronics. Qixiao Gai et al. [277] synthesized 2D CdS and 2D CoP NSs, oxidation and phosphidation process. Then, 2D–2D CdSe/CoP photo-catalysts were formed by ultrasonically dispersing the mixed solution of CdS and CoP. The CdSe/CoP with 2% CoP loading amounts showed a maximum photo-catalytic performance of 56.3 mmol g^−1^ h^−1^ under vis-light irradiation that is 11.3 times as high as bare CdS. The improved photo-catalytic activity of CdS eCoP should be due to the following two points: (1) high catalytic activity of CoP; (2) highly proficient separation and transfer of (e^−^–h^+^)-pairs photo-generated in CdS because of synthesized 2D–2D hetero-structure. In Table 1, H_2_-production-based photo-catalysts are summarized.

**Table 1:** H_2_-production based on photo-catalysts

Materials	Co-catalyst	Light source	H_2_ yield (μmol h^−1^g^−1^)	References
CdS	0.5 wt% Pd, 4 wt% MoS_2_, 5wt % polyaniline	Daylight fluorescent lamp	570	[278]
CdSe	Pt	*λ* = 300 W	1.65	[279]
UiO-66/CdS	1.5 wt% MoS_2_	*λ* ≥ 400 nm	12,426	[280]
In_2_S_3_/CdS	0.2 wt% MoS_2_	*λ* ≥ 420 nm	625.8	[281]
CdS	0.4 wt% rGO, 2 wt% MoS_2_	500 W UV–Vis lamp	6857	[282]
CdS	1.33 wt% graphene,0.67 wt% MoS_2_	*λ* ≥ 420 nm	9000	[283]
CdS	2 wt% single-layer (SL) MoS_2_	*λ* ≥ 420 nm	10,050	[284]
CdS	0.2 wt% MoS_2_	*λ* ≥ 420 nm	5330	[285]
CdS	0.2 wt% MoS_2_	*λ* ≥ 420 nm	~ 5400	[286]
CdS NSs	1% MoS_2_ NSs	*λ* ≥ 400 nm	8720	[287]
g-C_3_N_4_	–	*λ* > 420 nm	3.2	[288]
g-C_3_N_4_	Pt	*λ* > 420 nm	106.9	[288]
g-C_3_N_4_	Pt	*λ* > 300 nm	23,468	[288]
Graphene	CdS	300 W Xe	1050	[289]
1H-MoS_2_	–	100 W halogen	50	[290]
NrGO-MoS_2_	–	100/400 W halogen	10.8/42 k	[290]
1T-MoS_2_	–	100 W halogen	26,000	[290]
1T-MoSe_2_	–	100 W halogen	62,000	[291]
1T-WS_2_	TiO_2_	300 W Xe	2570	[292]
2H-WS_2_	TiO_2_	300 W Xe	225	[292]
MoS_2_	CdS	*λ* > 420 nm	1472	[293]
WS_2_	CdS	*λ* > 420 nm	1984	[24]
SnS_2_	–	300 W Xe	1060	[104]
ZnIn_2_S_4_	–	300 W Xe	57	[294]
ZnIn_2_S_4_	Pt	300 W Xe	213	[294]
Zn–In–S	Pt	400 W Hg	229	[295]
Zn–In–S	Pt + NaCl	400 W Hg	1056	[295]
TiO_2_	Pt	350 W Xe	1667.5	[296]
CdSe	2%CoP	350 W Xe	56.3	[277]
CdS	WS_2_	300 W Xe lamp	14.1	[297]

### Photo-catalytic Pollutant Degradation

**Fig. 26:**
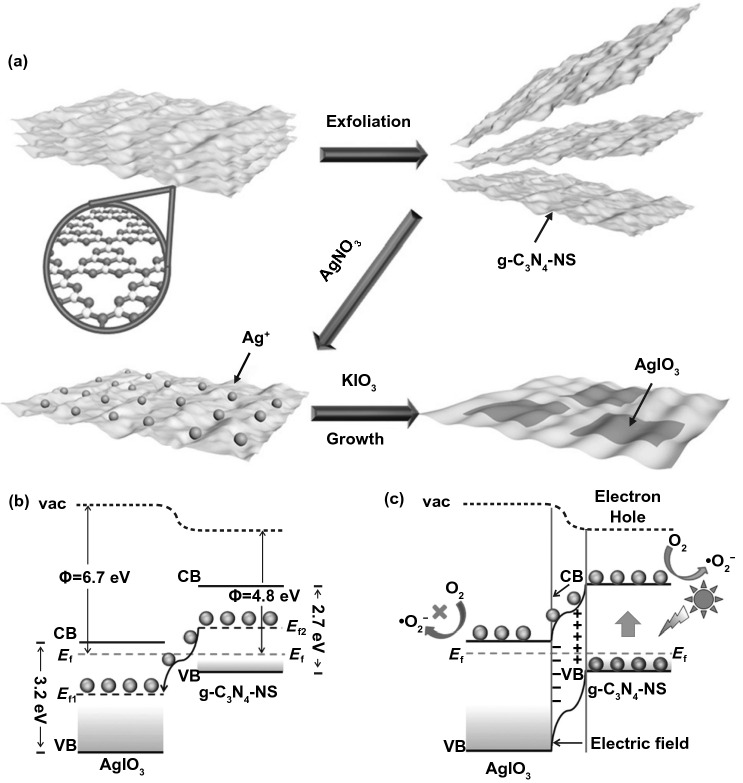
**a** Scheme showing AgIO_3_/g-C_3_N_4_-NSs composite synthesis. **b** Photo-catalytic degradation kinetics of RhB after vis-light irradiation. **c** Scheme showing charge separation at AgIO_3_/g-C_3_N_4_-NS interface after vis-light irradiation. Adapted with permission from Ref. [299]

In industrial progress, mass discharge of poisonous wastes (e.g., agrochemicals, dyes, and antibiotics) [136] has become worldwide a rigorous hazard to H_2_O resources and human physical condition [298]. Besides civilizing environment policies and system, development of an eco-friendly solution for eradicating pollution is dreadfully in demand. Among possible solutions, photo-catalytic decomposition of organic contaminants via in situ very reactive species is supposed to be green, cost-efficient, and talented move to deal with pollution matters. Even though numerous types of single ingredient photo-catalysts were developed, mostly suffer from a poor photo-catalytic activity and are not sufficiently efficient for real applications. A series of 2D/2D layer hetero-structures was generated for organic photo-degradation, showing potential uses. The 2D/2D hetero-structures consisting of AgIO_3_ and g-C_3_N_4_ NSs (AgIO_3_/g-C_3_N_4_-NS) were successfully formed for photo-catalytic waste H_2_O treatment after vis-light exposure by Li et al. [299]. The ultra-thin g-C_3_N_4_ NSs as polymeric organic SCs material were exhibiting superior vis-light response. Photo-catalytic performance of AgIO_3_/g-C_3_N_4_ NSs hetero-structures was considerably higher than that of single AgIO_3_ or g-C_3_N_4_ NSs for organic dyes degradation. Noticeably, degradation reaction rate constant of rhodamine B (RhB) than as-synthesized AgIO_3_/g-C_3_N_4_-NS sample was approximately 22.86 times higher as compared to hetero-structures composed of AgIO_3_ and bulk g-C_3_N_4_ (AgIO_3_/g-C_3_N_4_-B). It shows significance of 2DMs for construction of hetero-junction photo-catalysts (Fig. 26b).

A possible photo-catalytic method was also proposed in this work: The photo-induced e^−^s on CB of g-C_3_N_4_-NS can move to AgIO_3_ CB under vis-light irradiation that promoted photo-generated (e^−^–h^+^)-pairs separation in g-C_3_N_4_-NS (Fig. 26c). Considerably decreased steady-state PL spectra peak intensity and enhanced lifetime of charge carriers in PL spectra of AgIO_3_/g-C_3_N_4_ NSs as compared with that of single C_3_N_4_ NSs indicated superior e^−^-migration and charge separation effectiveness at hetero-junction edge. To consider O_2_/·O^2−^ reduction potential, the oxidative breakdown of the dye should be due to h^+^s in VB of the g-C_3_N_4_ NSs and •O^2−^ produced through e^−^s reduction of O_2_ from CB of g-C_3_N_4_ NSs. In another work, diverse quantity of BiOCl nano-plates is utilized to combine with C_3_N_4_ NSs via an easy calcination method. The face-to-face interaction edge of as-synthesized BiOCl/C_3_N_4_ hetero-structures was studied, and the significance of contact area of two components was discussed. The superior photo-current intensity and weakened PL intensity of BiOCl/C_3_N_4_ hetero-structures as compared with those of C_3_N_4_ NSs illustrated separation and migration of photo-generated e-s at interface. The BiOCl/C_3_N_4_ hetero-junction photo-catalysts were loaded with 70% BiOCl which showed the highest MO photo-degradation activity in vis-light irradiation. Wang et al. [300] prepared g-C_3_N_4_/Bi_2_WO_6_ 2D/2D hetero-structures consisting of g-C_3_N_4_ NSs and SL Bi_2_WO_6_ NSs for degradation of ibuprofen under vis-light irradiation. It shows that highly active photo-degradation system might be developed via navigation of charge division, transportation, and consumption at atomic level. Bera et al. [301] prepared a chain of hetero-structures consisting of rGO and CdS with diverse dimensionality (rGO/CdS) for photo-catalytic degradation of methylene blue (MB).

The 2D-G as an example of 2D-layered material, whose atomic thickness has outstanding charge transfer ability, can offer conducting e^−^-channels for separation of the photo-generated charges in the hetero-junction photo-catalyst composed of BG and SC. Through utilizing terephthalic acid (TA) as an example, the OH^•^ radicals were established to be generated active species for the photo-catalytic decomposition. Therefore, improved rGO/CdS activity in comparison with the single CdS might be explained as follows: Under vis-light irradiation, photo-generated e^−^ in CB of CdS is transferred to rGO surface and reacts with adsorbed O2 that consequently produces ·O^2−^ and OH^·^. Oxidization of both OH^•^ and photo-generated h^+^s leads to photo-catalytic decomposition of MB dye molecules. Charge migration from CdS to rGO takes place at rGO/CdS hetero-structures interface, as proved from considerably quenched PL of CdS components, promoting separation of photo-generated charges and therefore enhancing photo-catalytic activity [136]. In a few cases, the photo-catalytic activity of pollutant degradation might be increased through synergetic effect in hetero-junctions and other configuration tuning.

**Fig. 27:**
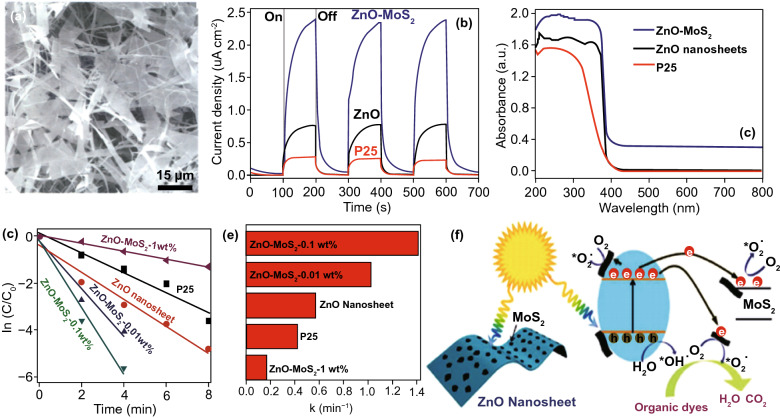
**a** ln (*C*/*C*_0_) versus time curves of MB with different photo-catalysts: ZnO/MoS_2_ hetero-structures with diverse doping amounts of MoS_2_ (mass ratio is 0, 0.01, 0.1, and 1 wt%) and commercially available P25. **b** Obvious rate constants of MB photo-degradation with different photo-catalysts. **c** Scheme showing photo-catalytic system of ZnO/MoS_2_ hetero-structures. Adapted with permission from Ref. [302]

Yangyang Liu et al. [302] studied a 2D/2D nano-composite photo-catalyst (ZnO/MoS_2_) derived from P-doped ZnO NSs with large SSA and 2D-MoS_2_ for competent photo-degradation of organic dyes (Fig. 27a–f). The ZnO/MoS_2_ hetero-structures with different MoS_2_ loading amounts (mass ratio is 0, 0.01, 0.1, and 1 wt%) and commercial P25 are utilized as photo-catalysts for photo-degradation calculations that took place in similar experimental conditions (Fig. 27d, e). Comparative findings illustrate that ZnO/MoS_2_ with a small loading amount of MoS_2_ (0.1 wt%) would considerably improve photo-catalytic activity in comparison with pristine ZnO NSs. When MoS_2_ loading amount was increased from 0.01 to 0.1 wt%, photo-catalytic performance of ZnO/MoS_2_ was further improved, but reduced after loading amount of MoS_2_ and further enhanced to 1 wt%. A huge MoS_2_-doped concentration would block sunlight that was utilized to force photo-degradation of MB and therefore decreases the photo-catalytic performance. A photo-catalytic mechanism of ZnO/MoS_2_ hetero-structures is proposed in Fig. 27f [136]. Derived from interfacial effect between ZnO and MoS_2_, photo-generated e^+^s would migrate from CB of ZnO to that of MoS_2_ that considerably improved the separation of carriers and therefore increase catalytic activity. Increased transport and separation of photo-generated h^+^s and e^−^s stimulated through interfacial effect can be established through increased photo-current density of MoS_2_/ZnO hetero-structures after introduction of MoS_2_ components. The photo-generated charges react with O_2_ and H_2_O after relocated to catalyst surface and generated much reactive radicals (OH and superoxide anion radicals) to degrade dye molecules. In addition to hetero-junction effect, P loading stimulated defects in ZnO NSs also support photo-catalytic activity as they can enhance light absorption via introduction of energy level between BGs [136].

### Photo-catalytic CO_2_ Reduction

**Fig. 28:**
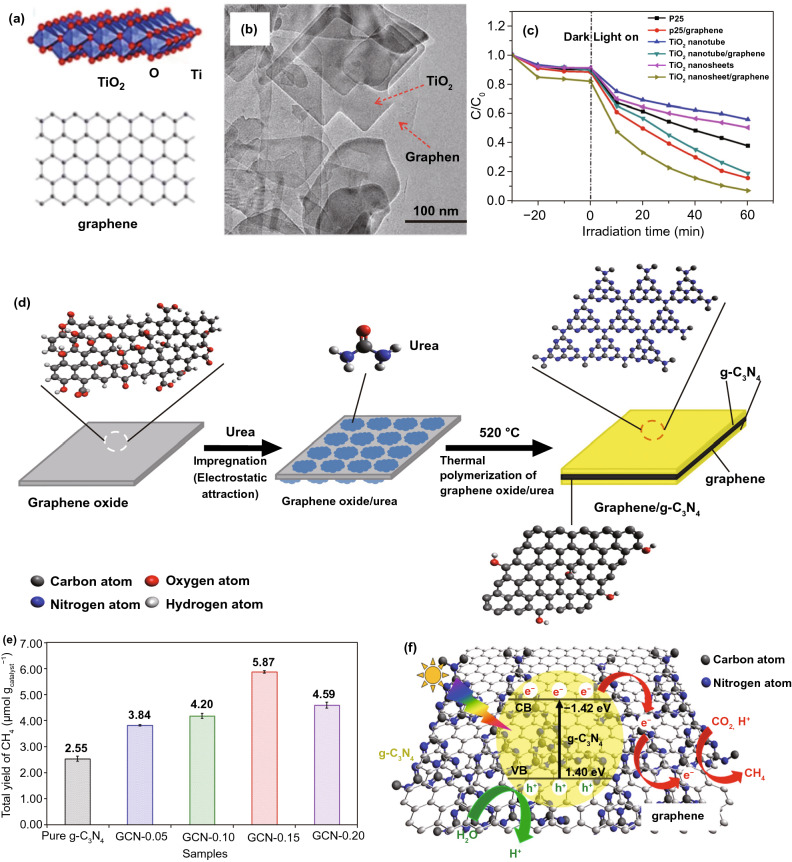
**a** TiO_2_ and graphene NS structures. **b** TEM and element mapping of graphene/TiO_2_ hetero-structure. **c** Photo-catalytic degradation of rhodamine B (RhB) over different photo-catalysts under irradiation of UV light. Adapted with permission from Ref. [304]. **d** Preparation of GCN samples through one-pot impregnation–thermal reduction approach. **e** Total CH_4_ yield over as-synthesized photo-catalysts. f Photo-generated charge transfer in GCN system for CO_2_ reduction with H_2_O to make CH_4_. Adapted with permission from Ref. [303]

The 2D materials aptitudes to boost the specific surface area to give elevated surface reactive sites make them a top precedence for photo-catalyst supports. Provided large contact area of 2D/2D composite, contact in graphene and photo-catalyst is anticipated to be enhanced that speeds up transfer and separation of photo-generated (e^−^–h^+^)-pairs, therefore enhancing their photo-catalytic CO_2_ reduction activity. According to the above-discussed phenomena, J. Sun et al. [257] fabricated three classes of 2D/0D, 2D/1D, and 2D/2D graphene/TiO_2_ hetero-structures through solvothermal method. So, the 2D/2D graphene/TiO_2_ hetero-structure showed maximum photo-catalytic effectiveness in contrast to 0D/2D and 1D/2D graphene/TiO_2_ hetero-structures and pristine TiO_2_ NSs. This performance was ascribed due to stronger electronic and physical coupling in 2D/2D structure that provides more proficient electron transport (Fig. 28a–c). Also, Wee-Jun Ong et al. [303] studied 2D/2D sandwich-like graphene-g-C_3_N_4_ (GCN) composite formed by one-pot impregnation thermal reduction technique. It also shows a high photo-catalytic activity for CO_2_ reduction to manufacture CH_4_. The large contact interface in graphene and g-C_3_N_4_ plays a significant function to improve photo-catalytic CO_2_ reduction by rising electron movement (Fig. 28d–f).

**Fig. 29:**
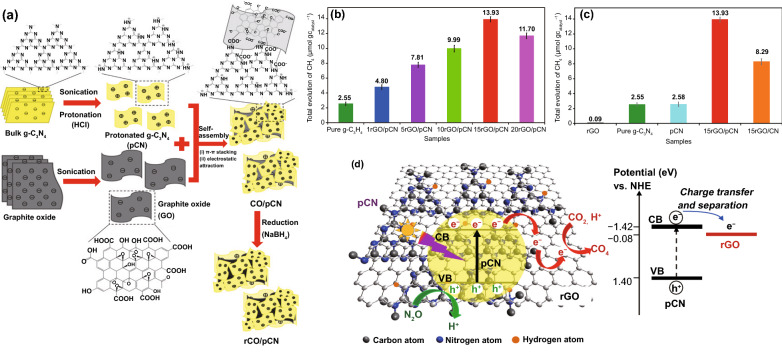
**a** Production method of rGO/pCN. **b** Total production of CH_4_ over pure g-C_3_N_4_ and a series of rGO/pCN photo-catalysts with different rGO contents under vis-light irradiation for 10 h. **c** Total evolution of CH_4_ after 10 h over rGO, pure g-C_3_N_4_, pCN, and 15rGO/CN. **d** Charge transfer and separation process happening in rGO/pCN nano-composite for CO_2_ reduction to CH_4_ in the occurrence of H_2_O under vis-light lighting. Adapted with permission from Ref. [305]

Furthermore, W. J. Ong et al. formed 2D/2D layered nano-structure of rGO/g-C_3_N_4_ by surface charge modification and protonation for improved photo-catalytic reduction of CO_2_ to CH_4_ [305]. The preparations involved ultrasonic dispersion with NaBH_4_-reduction method (Fig. 29a). In comparison with pristine g-C_3_N_4_ and rGO/CN, optimized composite 15 wt% rGO/pCN (15rGO/pCN) showed high CH_4_ production of 13.93 mmol g^−1^cat with a photochemical QY of 0.560% (Fig. 29b, c). Since g-C_3_N_4_ also showed 2D p-conjugated structure, this quality improves the capability of catalyst to adsorb CO_2_ molecules. When electrostatically charged with 2D conducting material rGO, pCN, and rGO created efficient interface, which caused improved performance for CO_2_ reduction (Fig. 29d).

In addition, formation method of 2D/2D structure may affect photo-catalytic activity; for example, exfoliation production of graphene and g-C_3_N_4_ will expose defects present on surface those are helpful for CO_2_ reduction, but surplus functional groups on catalyst surface will decrease conductance and number of active sites, therefore degrading activity. Therefore, Yu Teng Liang et al. [305] formed smaller quantity of surface defects that resulted in a higher photo-catalytic CO_2_ reduction activity toward CH_4_ formation as compared to their counterparts with high quantity of surface defects. In this method, graphene was formed by two different techniques that involved rGO solvent-exfoliated graphene (SEG) way and coupled with TiO_2_ to make rGO-TiO_2_ and SEG-TiO_2_, correspondingly. Due to its outstanding electron conductivity, SEG-TiO_2_ showed higher photo-catalytic CO_2_ reduction activity compared to rGO-TiO_2_. Hence, Table 2 shows the current developments in 2D materials for solar CO_2_ reduction devices.

**Table 2:** Comparison of current developments in 2D materials for solar-based CO_2_ reduction

Photo-catalyst materials	CO_2_ reduction product	Average rate (µmol g^−1^ h^−1^)	References
TiO_2_ NS-graphene	CH_4_	9.5	[306]
graphene-Ti_0.91_O_2_ hollow spheres	CH_4_/CO	1.14/8.91	[307]
WO_3_ NSs	CH_4_	16	[308]
MoS_2_-TiO_2_	CH_4_	10.6	[309]
BiOCl nano-plates	CO	8.1	[310]
BiOI/g‑C_3_N_4_	CO/CH_4_	4.86/0.18	[311]
Ag or Au/Zn-Ga-LDH	CO/CH_3_OH	300/2010	[312]
carbon-doped BN NSs	CO	9.3	[151]
Sandwich-like graphene/g-C_3_N_4_	CH_4_	5.87	[303]
rGO/protonated g-C_3_N_4_	CH_4_	13.93	[305]
GO	CH_3_OH	0.172	[313]
Boron-doped graphene (P25/B-GR)	CH_4_	2.5	[314]
Oxygen-rich TiO_2_-doped graphene oxide (5GO-OTiO_2_)	CH_4_	3.450	[315]
Cu_2_Se/graphene	CH_3_OH	2.63	[316]
rGO-TiO_2_	CH_4_	0.135	[317]
Cu_2_O/rGO	CH_3_OH	41.5	[318]
CsPbBr_3_ QDs/GO	CH_4_	29.6	[319]
TiO_2_-CdS/rGO	CH_4_	0.115	[320]
WO_3_ NS	CH_4_	16	[308]
MoS_2_/Bi_2_WO_6_	CH_3_OH	36.7	[321]
MoS_2_/TiO_2_	CH_4_	10.6	[309]
SnS_2_	CO	12.28	[322]
Bi_2_WO_6_	Methanol	75	[246]
ZnAl-LDH NSs	CO	7.6	–
Vv-rich o-BiVO_4_	Ethanol	398.3	–
Ti_3_C_2_ with P25	CH_4_		[252]

### Photo-catalytic H_2_O_2_ Production

Carbonaceous nanomaterials (NMs) with unique characteristics of sp^2^-hybridized carbon bonding with remarkable physicochemical nature at nanoscale usually show outstanding mechanical, chemical, and electrical properties [323]. In photo-catalysis research direction, carbonaceous NMs are frequently acted as e^−^ transfer materials and photo-sensitizer that can widen adsorption edge and advance migration effectiveness of photo-induced e^−^s [324]. While carbonaceous NMs are immobilized on g-C_3_N_4_ photo-catalyst, they can accept and transport photo-induced e^−^s from CB level of g-C_3_N_4_ and boost the reduction reaction activity and so lead to superior photo-catalytic performance [325]. As typical carbonaceous NMs, CNTs with *π*-conjugative structure are able to accept, transport, and store e^−^s [326]. So, g-C_3_N_4_-CNTs fabricated by incorporating the CNTs into g-C_3_N_4_ can advance photo-catalytic activity. Zhao and co-authors [327] applied the amination method to initiate CNTs in g-C_3_N_4_ NSs to make hybrid catalyst of g-C_3_N_4_/CNTs, where CNTs were covalently mixed with g-C_3_N_4_. The g-C_3_N_4_/CNTs hybrid photo-catalyst showed H_2_O_2_ formation rate of 32.6 μmol h^−1^ that was noticeably higher than g-C_3_N_4_ (2.5 μmol h^−1^). The CNTs covalent combined with g-C_3_N_4_ advanced e^−^ generation via higher reduction capability and favorably shifted CB level to improve single e^−^ reduction of O_2_ to ·O^2−^.

When polyoxometalates (POMs) [328] are irradiated through plentiful light energy, excitation of charge transfer from O^2−^ to Mn^+^ (*n* = 5, 6) is observed, guiding to development of h^+^ center (O^−^) and trapped e^−^ center (M_(*n*−1)_^+^) pair. So, excited POMs can provide e^−^ donors/acceptors. In addition, POMs have lesser recombination possibility of e^−^s and h^+^s, as a result of its distinct HOMO–LUMO BGs. Taking advantages of these, POMs have been broadly used in photo-catalysts areas, for example H_2_O oxidation [329], H_2_-evolution [330], CO_2_-reduction [331], etc. Thus far, POMs also combined with g-C_3_N_4_ for photo-catalytic H_2_O_2_ evolution. Zhao and co-workers [332] combined POM cluster of [PW_11_O_39_]_7_-(PW_11_) with 3D ordered macro-porous g-C_3_N_4_ (3DOM g-C_3_N_4_) for efficient photo-catalytic H_2_O_2_ evolution. POM covalent cluster of PW_11_ was covalently bonded with 3DOM g-C_3_N_4_ by captivating an organic linker approach. The quantity of synthesized H_2_O_2_ by g-C_3_N_4_/PW_11_ attained 3.5 μmol within 60 min, whereas catalytic activity of pristine 3DOM g-C_3_N_4_ was only 1.3 μmol. The CB and VB of 3DOM g-C_3_N_4_/PW_11_ were more positive compared to 3DOM g-C_3_N_4_ that improved their potential for H_2_O oxidation and promoted 2e^−^ reduction of O_2_ to H_2_O_2_. Similarly, they also studied a further POM cluster, [SiW_11_O_39_]8–(SiW_11_), to covalently combine with g-C_3_N_4_ [333].

Compared to PW_11_, the SiW_11_ possessed more negative CB level, helping 2e^−^ reduction of O_2_ to H_2_O_2_. Under sunlight irradiation (AM 1.5), H_2_O_2_ photo-catalytic production of 15.2 μmol h^−1^ over hybrid g-C_3_N_4_/SiW_11_ photo-catalyst was attained. Except for direct combination of POMs with g-C_3_N_4_, POMs-derived metal oxides were also incorporated with g-C_3_N_4_ to make hybrid photo-catalysts. Since POMs-derived metal oxides can accept, transport, and store e^−^s, resulting hybrid photo-catalysts are able to improve photo-induced e^−^s generation and therefore increase performance of reduction reaction for H_2_O_2_ formation. Zhao and Zhao [334] studied g-C_3_N_4_/PWO hybrid photo-catalyst through calcination of g-C_3_N_4_ precursor and (NH_4_)_3_PW_12_O_40_ (NH_4_-PW_12_) (POMs precursor). The hybrid g-C_3_N_4_/PWO photo-catalyst showed competent photo-catalytic appearance for photo-catalytic H_2_O_2_ fabrication by vis-light in the absence of organic e^−^ donor. In addition, a similar group [335] utilized another POMs-derived metal oxide to include g-C_3_N_4_. The g-C_3_N_4_/CoWO hybrid photo-catalyst was fabricated via calcination of 3-amino 1, 2, 4-triazole (3-AT) and (NH_4_)_8_Co_2_W_12_O_42_ (NH_4_-Co_2_W_12_). Under vis-light irradiation, H_2_O_2_ was rapidly generated over g-C_3_N_4_/CoWO and the amount of formed H_2_O_2_ was 18.7 μmol in 60 min, while individual g-C3N4 offered very lower photo-catalytic activity (< 0.1 μmol in 60 min). CoWO-incorporated g-C_3_N_4_ framework could produce more e^−^ for O_2_ reduction, while negative shifts of CB level from g-C_3_N_4_ to g-C_3_N_4_-CoWO enhanced single e^−^ reduction of O_2_ to ·O^2−^. Also, incorporated CoWO advanced the oxidation of ·O^2−^ to 1O_2_ by h^+^ and formed 1O_2_ proceeded 2e^−^ reduction to H_2_O_2_. Every one of these is related to enhanced photo-catalytic activity for H_2_O_2_ production over g-C_3_N_4_/CoWO hybrid photo-catalyst.

Organic counterparts have advantages of economic, simple formation and mechanical flexibility [336]. Mainly, organic photo-catalysts are capable to concentrate on faults of their inorganic counterparts, for example heavy metal with sensitive toxicity and restricted level of active sites. g-C_3_N_4_ were recognized as representative organic polymer SC photo-catalysts. Recently, other organic SCs were also employed as photo-catalysts, e.g., triazine and aromatic diimides. Aromatic diimides possess high e^−^ mobility and stability that are significant class of n-type organic SCs and incorporated with g-C_3_N_4_ for photo-catalytic H_2_O_2_ evolution. Shiraishi et al. [337] included facile way to aromatic diimide (pyromellitic diimide, PDI) in g-C_3_N_4_ network by facile thermal condensation. These results showed that both CB and VB levels were developed into more positive via incorporation of PDI units in g-C_3_N_4_ due to high e^−^ affinity of PDI. This detail exposed that g-C_3_N_4_/PDI photo-catalyst has superior ability for O_2_ reduction; therefore, H_2_O_2_ evolution from H_2_O and O_2_ was promoted. Additionally, efficient synthesis of 1,4-endoperoxide species on photoexcited g-C_3_N_4_/PDI suppressed 1e^−^ reduction of O_2_ and 4e^−^ reduction of O_2_, thus promoting selective two-e^−^ reduction of O_2_ to H_2_O_2_.

Likewise, other categories of aromatic diimides were also incorporated with g-C_3_N_4_, for example biphenyl diimide (BDI) [338] and mellitic triimide (MTI) [339]. In pristine H_2_O with O_2_, both g-C_3_N_4_/BDI and g-C_3_N_4_/MTI catalysts successfully produced millimolar levels of H_2_O_2_. For further activity improvement, rGO is incorporated with g-C_3_N_4_/PDI catalyst [340] that takes advantage from 2D single-carbon monolayer property of rGO with high charge carrier mobility and high photochemical and thermal stability. The g-C_3_N_4_/PDI/rGO_*x*_ nano-hybrids photo-catalyst was synthesized by hydrothermal–calcination process. The photo-catalytic reaction testing showed that g-C_3_N_4_/PDI/rGO_0.05_ produced the largest amount of H_2_O_2_ (29 μmol) under vis-light irradiation within 24 h, which was higher compared to g-C_3_N_4_ and g-C_3_N_4_/PDI. The SCC efficiency value of g-C_3_N_4_/PDI/rGO_0.05_ was up to 0.20% that was higher compared to other counterparts. In this photo-catalyst system, rGO not only encouraged efficient charge division but also increased selective 2e^−^ O_2_ reduction. The activity of g-C_3_N_4_/PDI-rGO photo-catalyst could be further improved by introducing BN because of spatial separation of e^−^ and h^+^ onto rGO and BN, correspondingly [341]. Yang et al. [342] fabricated perylene imides (PI) on g-C_3_N_4_ NSs to construct an all-solid-state Z-scheme hetero-junction. The hybrid g-C_3_N_4_/PI photo-catalyst with Z-scheme arrangement promoted spatial separation of charge carriers, where photo-induced e^−^s in PI recombined with h^+^s in g-C_3_N_4_, while remaining h^+^s and e^−^s were left on PI and g-C_3_N_4_, correspondingly. Consequently, more e^−^s from CB of g-C_3_N_4_ part reduced O_2_ to produce more H_2_O_2_, while h^+^s of g-C_3_N_4_/PI oxidized OH^−^ to ·OH that also later reacted to generate H_2_O_2_. The shift of H_2_O_2_ production from single-channel to two-channel leads to significant enhancement in photo-catalytic H_2_O_2_ evolution. In another study, anthraquinone (AQ) was fastened on g-C_3_N_4_ surface that attains analogous roles as other organic SCs [343].

Fei Xue et al. [344] studied efficient photo-catalytic pure H_2_O_2_ splitting for simultaneous H_2_ and H_2_O_2_ fabrication. Photo-catalytic overall H_2_O splitting for instantaneous H_2_ and H_2_O_2_ generation via a 2e^−^ pathway can readily address these issues. A novel Co_*x*_Ni_*y*_P cluster incorporated P-doped g-C_3_N_4_ photo-catalyst (Co_*x*_Ni_*y*_P-PCN) by two-step phosphating method that presents such unique behavior for pure H_2_O splitting into stoichiometric H_2_ and H_2_O_2_. The highest H_2_ production rate reaches 239.3 μmol h^−1^ g^−1^, achieved over CoNiP-PCN photo-catalyst that is among the best reported activities for overall H_2_O splitting. It is found that both P and cluster co-catalyst are critical to remarkably improved photo-catalytic activity. Specifically, P as a substitution of C in PCN introduces a positive charge center (P^+^), reinforcing chemical connection between PCN and CoNiP, in the form of P^+^–P^δ−^–Co^δ+^/Ni^δ+^. This unique bridging effect, together with extended light absorption by P doping and optimized surface redox potential by co-catalyst integration, stimulates efficient vectorial charge transfer between PCN and CoNiP and subsequent surface mass exchange. In contrast, this also shows that well-satisfied band structure of PCN can facilitate the 2e^−^ reaction pathway, which not only has implication for potential use of CoNiP-PCN as potential photo-catalyst for solar H_2_ manufacture, and offers a new idea for pure H_2_O splitting in particulate system [344]. Table 3 offers intuitive summary of photo-catalytic-based H_2_O_2_ production.

**Table 3:** Summary of photo-catalytic H_2_O_2_ production

Material	Sacrificial reagent	Photo-catalyst concentration	Irradiation conditions	H_2_O_2_ yields	References
TiO_2_	C_6_H_5_OH	10 mg mL^−1^	> 280 nm	40 mM (12 h)	[345]
TiO_2_	2-C_3_H_7_OH	1 mg mL^−1^	365 nm	423.2 μΜ (2 h)	[346]
Cu/TiO_2_	–	300 mg	300–400 nm	8 μM (5 min)	[347]
F-TiO_2_	HCOOH	0.5 g L^−1^	360 nm	1–1.3 mM	[347]
Au/TiO_2_	HCOOH	1 mg mL^−1^	> 420 nm	640–700 μΜ (1 h)	[337]
CoPi/rGO/TiO_2_	2-C_3_H_7_OH	0.5 g L^−1^	≥ 320 nm	4.5 mM (3 h)	[348]
TiO_2_/WO_3_/rGO	2-C_3_H_7_OH	1 mg mL^−1^	AM 1.5	~ 270 μΜ (1 h)	[349]
Au/TiO_2_	CH_3_OH	1 mg mL^−1^	> 320 nm	1.06 mΜ (3 h)	[350]
Au/SnO_2_-TiO_2_	Alcohol	1 mg mL^−1^	UV irradiation	~ 15 mM (25 h) -	[351]
g-C_3_N_4_	Alcohol	4 mg mL^−1^	> 420 nm	30 μmol (24 h)	[345]
Mesoporous g-C_3_N_4_	EtOH	4 mg mL^−1^	> 420 nm	90 μmol (24 h)	[352]
AQ-augmented g-C_3_N_4_	2-C_3_H_7_OH	0.5 mg mL^−1^	AM 1.5	361 μmol (1 h)	[343]
KPF_6_/g-C_3_N_4_	C_2_H_5_	0.5 mg mL^−1^	> 420 nm	1.5 mM (5 h)	[353]
Holey defective g-C_3_N_4_	H_2_O + IPA	0.83 mg mL^−1^	> 420 nm	12.1 μmol (2.5 h)	[354]
O_2_-enriched g-C_3_N_4_	H_2_O + C_3_H_7_OH	1 mg mL^−1^	> 420 nm	300 μmol (5 h)	[355]
g-C_3_N_4_-SiW_11_	CH_3_OH	1 mg mL^−1^	AM 1.5	15.2 μmol (1 h)	[356]
g-C_3_N_4_-CoWO	Organic e^−^	1 mg mL^−1^	≥ 420 nm	9.7 μmol (1 h)	[357]
[RuII (Me_2_phen)_3_]^2+^	O_2_-saturated H_2_SO_4_	1.0 μM	> 420 nm	612 μM (9 h)	[358]
Au/BiVO_4_	H_2_O + EtOH	50 mg/30 mL	> 420 nm	40.2 mM (10 h)	[359]
rGO/Cd_3_(TMT)_2_	H_2_O + C_2_H_5_OH	80 mg/20 mL	> 420 nm	~7 mM (24 h)	[360]
Resins	H_2_O	50 mg/30 mL	> 420 nm	99 μmol (24 h)	[361]

## Future Perspective and Challenges

In this review, a large number of experimental as well as theoretical research works related to photo-catalysts are discussed. A lot of confronts are required to be solved for incorporation of 2D p–n junctions in mass production electronic elements. Two most imperative confronts are scalable production of 2D p–n junctions and environment degradation of 2DMs. Despite significant growth in ultra-thin 2DMs for photo-catalysis, it is still suffering from large number of challenges in this direction. First, apart from extensively developed ultra-thin 2DMs, for instance OH, MOs, and sulfides for photo-catalytic process, other types of probable ultra-thin 2DMs with novel structure or electronic properties for photo-catalysis should be investigated, for instance layer oxy-halides (e.g., FeOBr and Bi_4_VO_8_Cl), thiophosphates (e.g., CoPS_3_), multi-metal chalcogenides (for instance Cu_2_ZnGeS_4_), or metal-free SCs (e.g., C_3_N and C_2_N). Specially, ultra-thin 2DMs by means of intrinsic non-vdWs’ layer structure have great potential in photo-catalysis, since plentiful surface atoms along dangling bonds can assist to construct outstanding chemical surroundings for supporting molecular reaction chemisorptions and enhance catalytic reaction dynamics. Second, there is an exceptional large-scale approach to yield ultra-thin 2D photo-catalysts with controlled thickness or crystal structures. The scalable manufacturing of ultra-thin 2D photo-catalysts has immense importance for possible commercial applications, and therefore more concentration should be given to cost-efficient scalable production approach. Third, atomic-scale thicknesses permit ultra-thin materials along simply adjusting electronic structures that show an important effect on photo-catalytic performance. In spite of defect engineering, element doping, etc., or other efficient approaches for instance, engineering or tensile strain of surface state should be modified to engineer electronic structure and therefore enormously promote photo-catalytic activity. Fourth, ultra-thin 2D arrangement offers a perfect material model to distinguish catalysts’ active site, additional types of tuning, and reaction centers quantity to fulfill definite catalytic process requirement.

### Scalable Synthesis and Environmental Degradation

First major confront is concerned with scalable synthesis of engineered vdWs hetero-structures with well-controlled edges. Still if deterministic assigned methods are flourishing at experimental level, they are not suitable for commercial purposes. Growth methods, such as CVD growth, have already been proven proficient for the growth of high-quality 2DMs such as lateral and vertical hetero-structures at laboratory level. The VdWs epitaxial methods are even more promising for fabrication of high-quality 2D hetero-structures. Upscale of such growth methods is probable, and the upcoming years will realize the application of higher-quality devices. A second, potential approach is to upscale growth of 2D p–n junctions to merge development of single 2DMs (e.g., MoS_2_) with different doping methods (frequently electrostatic or chemical). A second confront is environment degradation of numerous recognized 2DMs. For instance, while exposed to air, the BP in its ultra-thin structure is likely to absorb humidity that degrades material electronic properties. In context of BP, the most established degradation mechanism such as material reaction with O_2_ alters material properties. One method that prevents this degradation is encapsulating air-sensitive material among h-BN flakes under O^2−^ and humidity-free conditions. One active area of 2DMs investigation is consequently devoted to upscale encapsulation methods. A special move, which is at present practiced, is active investigation for novel 2DMs that did not create degradation problems, which could arrive either from the preparation (e.g., TiS_3_) or from natural resources (e.g., franckeite). Such, active search previously assisted to multiply number of present 2DMs only a handful more than twenty under 10 years.

### Future Perspectives

Other than traditional optoelectronic applications, 2D p–n junctions have still a lot of unknown applications and fundamental problems. For example, thermoelectric applications of 2D p–n junctions were not yet completely studied. The conventional Peltier device, a component mainly utilized in electronics for cooling (and commonly less for heating), depends on p- and n-type SC thermally coupled in parallel and series. The VdWs hetero-structures could be utilized to manufacture atomic-level thin cooling (or heating) elements in combination with other smart properties of 2DMs, e.g., high transparency or flexibility. Another exciting way is the study of new p-n junction geometries (e.g., circular p-n junctions in recent times showed in G) or novel devices based on 2D p-n junctions, e.g., logic gates or memories. Genuine probabilities still buried in 2DMs are in all possibilities than one described and 2D p–n junctions grasp much promise in larger-scale applications. Such 2D-junctions are particularly attractive building blocks of inflexible and transparent electronics, e.g., light-emitting diodes (LEDs) or solar cells. One more application, which can advantage from ultra-thin structure of 2D p–n junctions, is light-sensing and harvesting applications for nano-photonics. The 2D p–n junctions can be utilized as photodetectors, and numerous material combinations are present, which can be utilized to propose devices with sensible wavelengths ranging from infrared to UV that have already been established.

## Conclusion

In recent years, new p–n junctions manufacturing witnessed benefit of an ultra-thin nature of 2DMs. The top-down and bottom-up production methods have established competent generating p-n junctions with high optoelectronic properties. 2DMs continue to offer numerous prospects to manufacture new p-n junctions with exceptional properties, which unlock motivating scientific directions both in requisites of elementary questions and with respect to applications [32]. Ultra-thin 2DMs and their hybridizations through maintaining 2D arrangement are outstanding materials for elementary photo-catalytic investigation and promising marketable uses. This broad review highlights modern advancement in appliance of ultra-thin 2DMs for the photo-catalytic solar energy conversion. First, this review offers a complete outline of categorization and controlled fabrication mechanism of an ultra-thin 2D photo-catalyst. After that, approaches to modify electronic arrangement of ultra-thin2DMs and more effecting photo-catalytic properties are reviewed, that is, an element engineering, thickness engineering, defect engineering and doping. In addition, further hybridizations with upholding ultra-thin 2D characteristics are offered to further enhance photo-catalytic properties, such as QDs/2DMs, single atoms/2DMs, molecular/2DMs, and 2D–2D stacking materials. Lastly, a variety of photo-catalytic applications over ultra-thin 2D photo-catalysts were reviewed with emphasis on insights into structure–performance relationship, involving H_2_O oxidation, H_2_ evolution, CO_2_ reduction, N_2_ fixation, organic synthesis, and pollutants degradation. In conclusion, this review highlights universal approaches and current growth in 2D/2D hetero-junctions and hetero-structures that are outstanding candidates for basic investigation and possible catalyst applications because of their exclusive electronic structure and physicochemical properties:

1. Integrating their components’ advantages, for instance ultra-thin 2D configuration, large surface area, and electronic/physicochemical properties;
2. Partition or charge transfer can be encouraged for required function; and
3. Versatile options (such as thickness, elements, defects, fabrication expertise, and contact space) can be designed to engineer properties and therefore application activity [136].

Heterogeneous photo-catalysis has turned into a fast-growing galaxy with manifold miscellaneous matters being investigated and introduced. Driven through aforementioned benefits and possible standards of 2D/2D hetero-structures, increasing amount of extraordinary achievements were established in last few years. However, with challenges creating hurdles for real catalysis applications, e.g., catalyst competency, selectivity related to yield and pollution, environment friendly, and cost-efficient, there are still a lot of efforts to follow the purpose of developing required catalyst and reaction scheme for real-world applications. Catalysts grasp key for increasing effectiveness of catalytic reaction method which is the entrance of commercial practicable applications required to be prevailed over. The 2D/2D hetero-structures as photo-catalysts are planned with purpose to endorse light harvesting, charge carrier separation/transfer, redox reactivity, etc. In addition to sufficiently intriguing benefits of 2D elements via very well engineering of structure, composition, BG, and surface reaction sites, interfacial tuning at nanoscale of 2D/2D hetero-structures is probable to further make easy photo-catalytic activity. Even though a lot of opportunities subsist for 2D/2D hetero-structures as electrocatalysts, this field is also facing a lot of challenges and research room:

1. Synthetic techniques are further required to be optimized to persuade commercial manufacturing demands.
2. Analysis techniques might be more superior for structure, physicochemical properties, and activity for these ultra-thin hetero-structures.
3. Clear comprehension of work mechanism, in particular reaction intermediates, of 2D/2D hetero-junction electrocatalysts is still now required.

In addition, aforementioned optimization method of designing catalyst, high products yield/selectivity, and development of novel organic materials preparation are all solutions for further investigation. As a result of quick advancement and affluent information accumulated in the 2DMs and hetero-structures in last year’s, one may anticipate that 2D/2D hetero-structures would participate in a significant role in resolving energy and environment confronts [136]. However, investigations concentrating on the photo-catalysis mechanism are still insufficient. A lot of hard work should be required to optimize the theoretical calculation setting, nearer to the industrial-scale practical reaction conditions. Parallel exploitation of theoretical approach along superior experimental approach can assist to get deeper understanding for connection between neighboring atomic microstructure and activity as well as elucidate reaction methods. Also, due to relevant limitations of every material, a preferred move to obtain optimal properties is performed to assemble diverse building blocks to produce supportive results. It is supposed that such a broad review will further put research in the field of 2DMs as a novel photo-catalysis [32].

## References

[1] Huang K, Li Z, Lin J, Han G, Huang P (2018). Two-dimensional transition metal carbides and nitrides (mxenes) for biomedical applications. Chem. Soc. Rev..

[2] Khan K, Tareen AK, Aslam M, Thebo KH, Khan U (2018). A comprehensive review on synthesis of pristine and doped inorganic room temperature stable mayenite electride, [Ca_24_Al_28_O_64_]^4+^(e^−^)_4_ and its applications as a catalyst. Prog. Solid State Chem..

[3] Khan K, Tareen AK, Aslam M, Mahmood A, Khan Q (2019). Going green with batteries and supercapacitor: two dimensional materials and their nanocomposites based energy storage applications. Prog. Solid State Chem..

[4] Khan K, Tareen AK, Aslam M, Zhang Y, Wang R, Ouyang Z, Gou Z, Zhang H (2019). Recent advances in two-dimensional materials and their nanocomposites in sustainable energy conversion applications. Nanoscale.

[5] Dincer I (2000). Renewable energy and sustainable development: a crucial review. Renew. Sust. Energy Rev..

[6] Zou X, Zhang Y (2015). Noble metal-free hydrogen evolution catalysts for water splitting. Chem. Soc. Rev..

[7] Chia X, Eng AYS, Ambrosi A, Tan SM, Pumera M (2015). Electrochemistry of nanostructured layered transition-metal dichalcogenides. Chem. Rev..

[8] Yin H, Tang Z (2016). Ultrathin two-dimensional layered metal hydroxides: an emerging platform for advanced catalysis, energy conversion and storage. Chem. Soc. Rev..

[9] Shi Y, Li H, Li LJ (2015). Recent advances in controlled synthesis of two-dimensional transition metal dichalcogenides via vapour deposition techniques. Chem. Soc. Rev..

[10] Jiao Y, Zheng Y, Jaroniec M, Qiao SZ (2015). Design of electrocatalysts for oxygen-and hydrogen-involving energy conversion reactions. Chem. Soc. Rev..

[11] Pang J, Mendes RG, Bachmatiuk A, Zhao L, Ta HQ (2019). Applications of 2d mxenes in energy conversion and storage systems. Chem. Soc. Rev..

[12] Jin H, Guo C, Liu X, Liu J, Vasileff A, Jiao Y, Zheng Y, Qiao SZ (2018). Emerging two-dimensional nanomaterials for electrocatalysis. Chem. Rev..

[13] Zhang Z, Penev ES, Yakobson BI (2017). Two-dimensional boron: structures, properties and applications. Chem. Soc. Rev..

[14] Meng Q, Xiu RW, Taeho J, Miae W, Young PG (2018). Omnipotent phosphorene: a next-generation, two-dimensional nanoplatform for multidisciplinary biomedical applications. Chem. Soc. Rev..

[15] Dai L, Chang DW, Baek JB, Lu W (2012). Carbon nanomaterials: carbon nanomaterials for advanced energy conversion and storage. Small.

[16] Khan K, Jia L, Wenwei Z, Wei X, Ye Y, Weijie S (2016). Low temperature synthesis of nano porous 12Cao·7Al_2_O_3_ powder by hydrothermal method. J. Wuhan Univ. Technol.-Mater. Sci. Ed..

[17] Khan K, Tareen AK, Elshahat S, Yadav AK, Khan U (2018). Facile synthesis of cationic doped [Ca_24_Al_28_O_64_]^4+^.(4e^−^) composite via rapid citrate sol–gel method. Dalton Trans..

[18] Khan K, Tareen AK, Li J, Khan U, Nairan A (2018). Facile synthesis of tin-doped mayenite electride composite as a non-noble metal durable electrocatalyst for oxygen reduction reaction (ORR). Dalton Trans..

[19] Khan K, Tareen AK, Aslam M, Wang R, Zhang Y (2019). Recent developments in emerging two-dimensional materials and their applications. J. Mater. Chem. C.

[20] Khan K, Tareen AK, Aslam M, Khan SA, Khan Q (2019). Fe-doped mayenite electride composite with 2d reduced graphene oxide: as a non-platinum based, highly durable electrocatalyst for oxygen reduction reaction. Sci. Rep..

[21] Khan K, Tareen AK, Aslam M, Khan Q, Khan SA (2019). Novel two-dimensional carbon–chromium nitride-based composite as an electrocatalyst for oxygen reduction reaction. Front. Chem..

[22] Khan K, Tareen AK, Aslam M, Wang R, Zhang Y (2020). Recent developments in emerging two-dimensional materials and their applications. J. Mater. Chem. C.

[23] Khan K, Tareen AK, Elshahat S, Muhammad N, Li J (2018). Facile metal-free reduction-based synthesis of pristine and cation-doped conductive mayenite. RSC Adv..

[24] Khan K, Tareen AK, Khan U, Nairan A, Elshahat S (2018). Single step synthesis of highly conductive room-temperature stable cation-substituted mayenite electride target and thin film. Sci. Rep..

[25] Tareen AK, Priyanga GS, Khan K, Pervaiz E, Thomas T, Yang M (2019). Nickel-based transition metal nitride electrocatalysts for the oxygen evolution reaction. Chemsuschem.

[26] Zou W, Khan K, Zhao X, Zhu C, Huang J (2017). Direct fabrication of C12A7 electride target and room temperature deposition of thin films with low work function. Mater. Res. Express.

[27] Khan K, Tareen AK, Aslam M, Khan MF, Shi Z (2020). Synthesis, properties and novel electrocatalytic applications of the 2-D borophene xenes. Prog. Solid State Chem..

[28] Fan W, Zhang Q, Wang Y (2013). Semiconductor-based nanocomposites for photocatalytic H_2_ production and Co_2_ conversion. Phys. Chem. Chem. Phys..

[29] Luo B, Liu G, Wang L (2016). Recent advances in 2D materials for photocatalysis. Nanoscale.

[30] Zhang WJ, Li WW, Chen XG, Hu ZG, Liu W (2011). Phonon mode and phase transition behaviors of (1 − x)PbSc_1/2_Ta_1/2_O_3−x_PbHfO_3_ relaxor ferroelectric ceramics determined by temperature-dependent raman spectra. Appl. Phys. Lett..

[31] Bonito RD, Elliott ML, Jardinm EAD (1995). Detection of an arbuscular mycorrhizal fungus in roots of different plant species with the PCR. Appl. Environ. Microb..

[32] Di J, Xiong J, Li H, Liu Z (2017). Ultrathin 2d photocatalysts: electronic-structure tailoring, hybridization, and applications. Adv. Mater..

[33] Ran J, Zhang J, Yu J, Jaroniec M, Qiao SZ (2015). Earth-abundant cocatalysts for semiconductor-based photocatalytic water splitting. Chem. Soc. Rev..

[34] Ma Y, Wang X, Jia Y, Chen X, Han H, Li C (2014). Titanium dioxide-based nanomaterials for photocatalytic fuel generations. Chem. Rev..

[35] Equally LTC, Zhang J, Li LZ, Kumar NA, Zhao XS (2016). Functionalization of chemically derived graphene for improving its electrocapacitive energy storage properties. Energy Environ. Sci..

[36] Ong WJ, Tan LL, Ng YH, Yong ST, Chai SP (2016). Graphitic carbon nitride (g-C3N4)-based photocatalysts for artificial photosynthesis and environmental remediation: are we a step closer to achieving sustainability?. Chem. Rev..

[37] Fujishima A, Honda K (1972). Electrochemical photolysis of water at a semiconductor electrode. Nature.

[38] Tosine HM, Lawrence J, Carey JH (1976). Photodechlorination of PCB’s in the presence of titanium dioxide in aqueous suspensions. B-Environ. Contam. Toxicol..

[39] Zhou X, Liu N, Schmidt J, Kahnt A, Osvet A (2017). Noble-metal-free photocatalytic hydrogen evolution activity: the impact of ball milling anatase nanopowders with TiH_2_. Adv. Mater..

[40] Choi WB, Chung DS, Kang JH, Kim HY, Jin YW (1999). Fully sealed, high-brightness carbon-nanotube field-emission display. Appl. Phys. Lett..

[41] Hisatomi T, Maeda K, Takanabe K, Kubota J, Domen K (2009). Aspects of the water splitting mechanism on (Ga1 − xZnx)(n1 − xOx) photocatalyst modified with Rh2–YCrYo3 cocatalyst. J. Phys. Chem. C.

[42] Hisatomi T, Kubota J, Domen K (2014). Recent advances in semiconductors for photocatalytic and photoelectrochemical water splitting. Chem. Soc. Rev..

[43] Bao Q, Zhang H, Wang B, Ni Z, Lim CHYX (2011). Broadband graphene polarizer. Nat. Photonics.

[44] Tao W, Kong N, Ji XY, Zhang YP, Sharma A (2019). Emerging two-dimensional monoelemental materials (Xenes) for biomedical applications. Chem. Soc. Rev..

[45] Tan C, Cao X, Wu XJ, He Q, Yang J (2017). Recent advances in ultrathin two-dimensional nanomaterials. Chem. Rev..

[46] Novoselov KS, Geim AK, Morozov SV, Jiang D, Zhang Y (2004). Electric field effect in atomically thin carbon films. Science.

[47] Sun Y, Gao S, Lei F, Xie Y (2014). Atomically-thin two-dimensional sheets for understanding active sites in catalysis. Chem. Soc. Rev..

[48] Du Z, Yang S, Li S, Lou J, Zhang S (2020). Conversion of non-van der waals solids to 2d transition-metal chalcogenides. Nature.

[49] Su T, Shao Q, Qin Z, Guo Z, Wu Z (2018). Role of interfaces in two-dimensional photocatalyst for water splitting. ACS Catal..

[50] Low J, Cao S, Yu J, Wageh S (2014). Two-dimensional layered composite photocatalysts. Chem. Commun..

[51] Kouser S, Thannikoth A, Gupta U, Waghmare UV, Rao CNR (2015). 2D-gas as a photocatalyst for water splitting to produce H_2_O_2_. Small.

[52] Chao Z, Wang L, Gang L, Gao QL, Cheng HM (2013). Template-free synthesis of Ta_3_N_5_ nanorod arrays for efficient photoelectrochemical water splitting. Chem. Commun..

[53] Singh N, Jabbour G, Schwingenschlgl U (2012). Optical and photocatalytic properties of two-dimensional MoS_2_. Eur. Phys. J. B.

[54] Li Q, Li X, Wageh S, Al-Ghamdi AA, Yu J (2015). CdS/graphene nanocomposite photocatalysts. Adv. Energy Mater..

[55] Li Y, Li YL, Sa B, Ahuja R (2017). Review of two-dimensional materials for photocatalytic water splitting from a theoretical perspective. Catal. Sci. Technol..

[56] Chen GF, Ma TY, Liu ZQ, Li N, Qiao SZ (2016). Efficient and stable bifunctional electrocatalysts Ni/Ni_x_ M_y_ (M = P, S) for overall water splitting. Adv. Funct. Mater..

[57] Monai M, Melchionna M, Fornasiero P (2018). Chapter One - From metal to metal-free catalysts: Routes to sustainable chemistry. Adv. Catal..

[58] Tian T, Li Y, Xie D, Shen Y, Ren J (2012). Clinical features and risk factors for post-partum depression in a large cohort of chinese women with recurrent major depressive disorder. J. Affect. Disord..

[59] Li X, Lin MW, Lin J, Huang B, Puretzky AA (2016). Two-dimensional GaSe/MoSe_2_ misfit bilayer heterojunctions by van der waals epitaxy. Sci. Adv..

[60] Liang Q, Ye L, Huang ZH, Xu Q, Bai Y, Kang F, Yang QH (2014). A honeycomb-like porous carbon derived from pomelo peel for use in high-performance supercapacitors. Nanoscale.

[61] Kobayashi R, Tanigawa S, Takashima T, Ohtani B, Irie H (2014). Silver-inserted heterojunction photocatalysts for z-scheme overall pure-water splitting under visible-light irradiation. J. Phys. Chem. C.

[62] Lin B, Li H, An H, Hao W, Wei J (2018). Preparation of 2d/2d g-C_3_N_4_ nanosheet@ZNiN_2_S_4_ nanoleaf heterojunctions with well-designed high-speed charge transfer nanochannels towards high-efficiency photocatalytic hydrogen evolution. Appl. Catal. B Environ..

[63] Xiao H, Tan C, Zhang ZYA (2014). 25th anniversary article: hybrid nanostructures based on two-dimensional nanomaterials. Adv. Mater..

[64] Wang YJ, Tao YM, Li FY, Wang YH, Xu XJ (2009). Pharmacological characterization of ATPM [(–)-3-aminothiazolo[5,4-b]-N-cyclopropylmethylmorphinan hydrochloride], a novel mixed κ-agonist and μ-agonist/-antagonist that attenuates morphine antinociceptive tolerance and heroin self-administration behavior. J. Pharmacol. Exp. Ther..

[65] Yang J, Wang D, Han H, Li C (2013). Roles of cocatalysts in photocatalysis and photoelectrocatalysis. Acc. Chem. Res..

[66] Melchionna M, Fornasiero P (2020). Updates on the roadmap for photocatalysis. ACS Catal..

[67] Ohtani B (2008). Preparing articles on photocatalysis-beyond the illusions, misconceptions, and speculation. Chem. Lett..

[68] Zhang J, Huang Y, Jin L, Rosei F, Vetrone F, Claverie JP (2017). Efficient upconverting multiferroic core@shell photocatalysts: visible-to-near-infrared photon harvesting. ACS Appl. Mater. Interfaces..

[69] Wang L, Xu X, Cheng Q, Dou SX, Du Y (2019). Near-infrared-driven photocatalysts: design, construction, and applications. Small.

[70] Freitag M, Möller N, Rühling A, Strassert CA, Ravoo BJ, Glorius F (2019). Photocatalysis in the dark: near-infrared light driven photoredox catalysis by an upconversion nanoparticle/photocatalyst system. ChemPhotoChem.

[71] Chen H, Liu W, Hu B, Qin Z, Liu H (2017). A full-spectrum photocatalyst with strong near-infrared photoactivity derived from synergy of nano-heterostructured Er^3+^-doped multi-phase oxides. Nanoscale.

[72] Braslavsky SE, Braun AM, Cassano AE, Emeline AV, Litter MI (2011). Glossary of terms used in photocatalysis and radiation catalysis. Pure Appl. Chem..

[73] Munoz-Batista MJ, Caudillo-Flores U, Ung-Medina F, del Carmen Chávez-Parga M, Cortés JA, Kubacka A, Fernández-García M (2017). Gas phase 2-propanol degradation using titania photocatalysts: study of the quantum efficiency. Appl. Catal. B Environ..

[74] Shelef M, McCabe RW (2000). Twenty-five years after introduction of automotive catalysts: what next?. Catal. Today.

[75] Sui S, Wang X, Zhou X, Su Y, Riffat S, Liu C-J (2017). A comprehensive review of pt electrocatalysts for the oxygen reduction reaction: nanostructure, activity, mechanism and carbon support in PEM fuel cells. J. Mater. Chem. A.

[76] Kou J, Lu C, Wang J, Chen Y, Xu Z, Varma RS (2017). Selectivity enhancement in heterogeneous photocatalytic transformations. Chem. Rev..

[77] Ruiz-Mercado GJ, Smith RL, Gonzalez MA (2012). Sustainability indicators for chemical processes: i. Taxonomy. Ind. Eng. Chem. Res..

[78] S.M. Fortier, N.T. Nassar, G.W. Lederer, J. Brainard, J. Gambogi, E.A. McCullough, Draft critical mineral list-summary of methodology and background information US geological survey technical input document in response to secretarial order no. 3359. 2018-1021 (2018). 10.3133/ofr20181021

[79] Gulley AL, Nassar NT, Xun S (2018). China, the united states, and competition for resources that enable emerging technologies. Proc. Natl. Acad. Sci. U.S.A..

[80] Paik T, Cargnello M, Gordon TR, Zhang S, Yun H (2018). Photocatalytic hydrogen evolution from substoichiometric colloidal WO_3−x_ nanowires. ACS Energy Lett..

[81] Carraro G, Maccato C, Gasparotto A, Montini T, Turner S (2014). Enhanced hydrogen production by photoreforming of renewable oxygenates through nanostructured Fe_2_O_3_ polymorphs. Adv. Funct. Mater..

[82] Qamar S, Lei F, Liang L, Gao S, Liu K (2016). Ultrathin TiO_2_ flakes optimizing solar light driven CO_2_ reduction. Nano Energy.

[83] Gao S, Sun Y, Lei F, Liu J, Liang L (2014). Freestanding atomically-thin cuprous oxide sheets for improved visible-light photoelectrochemical water splitting. Nano Energy.

[84] Lei F, Sun Y, Liu K, Gao S, Liang L, Pan B, Xie Y (2014). Oxygen vacancies confined in ultrathin indium oxide porous sheets for promoted visible-light water splitting. J. Am. Chem. Soc..

[85] Wang J, Liu CJ (2015). Preparation of 2D WO_3_ nanomaterials with enhanced catalytic activities: current status and perspective. Chembioeng. Rev..

[86] Wang L, Sasaki T (2014). Titanium oxide nanosheets: graphene analogues with versatile functionalities. Chem. Rev..

[87] Sakai N, Ebina Y, Takada K, Sasaki T (2005). Photocurrent generation from semiconducting manganese oxide nanosheets in response to visible light. J. Phys. Chem. B.

[88] Ma R, Sasaki T (2011). Nanosheets of oxides and hydroxides: ultimate 2d charge-bearing functional crystallites. Adv. Mater..

[89] Akatsuka K, Takanashi G, Ebina Y, Haga MA, Sasaki T (2012). Electronic band structure of exfoliated titanium- and/or niobium-based oxide nanosheets probed by electrochemical and photoelectrochemical measurements. J. Phys. Chem. C.

[90] Ida S, Ogata C, Eguchi M, Youngblood WJ, Mallouk TE, Matsumoto Y (2008). Photoluminescence of perovskite nanosheets prepared by exfoliation of layered oxides, K_2_Ln_2_Ti_3_O_10_, KLnNb_2_o_7_, and RbLnTa_2_O_7_ (ln: lanthanide ion). J. Am. Chem. Soc..

[91] Maeda K, Eguchi M, Oshima T (2015). Perovskite oxide nanosheets with tunable band-edge potentials and high photocatalytic hydrogen-evolution activity. Angew. Chem. Int. Ed..

[92] Yu H, Sun Q, Jia X, Wang X, Yu J (2015). Facile synthesis of porous Bi_2_WO_6_ nanosheets with high photocatalytic performance. Dalton Trans..

[93] Tae EL, Lee KE, Jeong JS, Yoon KB (2008). Synthesis of diamond-shape titanate molecular sheets with different sizes and realization of quantum confinement effect during dimensionality reduction from two to zero. J. Am. Chem. Soc..

[94] Zhou Y, Zhang Y, Lin M, Long J, Zhang Z (2015). Monolayered Bi_2_WO_6_ nanosheets mimicking heterojunction interface with open surfaces for photocatalysis. Nat. Commun..

[95] Li J, Qin S, Xu J, Xiong J, Wu C (2016). Randomized, double-blind, placebo-controlled phase iii trial of apatinib in patients with chemotherapy-refractory advanced or metastatic adenocarcinoma of the stomach or gastroesophageal junction. J. Clin. Oncol..

[96] Zhang T, Zhou C, Zhao Y, Bian T, Shang L (2013). Bubble template synthesis of Sn_2_Nb_2_O_7_ hollow spheres for enhanced visible-light-driven photocatalytic hydrogen production. Chem. Commun..

[97] Zhao Y, Chen G, Bian T, Zhou C, Zhang T (2015). Defect-rich ultrathin ZnAl-layered double hydroxide nanosheets for efficient photoreduction of CO_2_ to CO with water. Adv. Mater..

[98] Liu YR, Loh EW, Lan TH, Chen SF, Yu YH (2010). ADRA1A gene is associated with BMI in chronic schizophrenia patients exposed to antipsychotics. Pharmacogenomics J..

[99] Zhao Y, Li B, Wang Q, Gao W, Wang CJ (2014). NiTi-layered double hydroxides nanosheets as efficient photocatalysts for oxygen evolution from water using visible light. Chem. Sci..

[100] Hasani A, Tekalgne M, Le QV, Jang HW, Kim SY (2019). Two-dimensional materials as catalysts for solar fuels: hydrogen evolution reaction and CO_2_ reduction. J. Mater. Chem A.

[101] Xu Y, Zhao W, Xu R, Shi Y, Zhang B (2013). Synthesis of ultrathin CdS nanosheets as efficient visible-light-driven water splitting photocatalysts for hydrogen evolution. Chem. Commun..

[102] He Q, Li C, Geng F, Yang H, Li P (2012). Aerosol optical properties retrieved from sun photometer measurements over Shanghai, China. J. Geophys. Res. Atmos..

[103] Li H, Wu J, Yin Z, Zhang H (2014). Preparation and applications of mechanically exfoliated single-layer and multi layer MoS_2_ and WSe_2_ nanosheets. ACS Chem. Res..

[104] Yu J, Xu CY, Ma FX, Hu SP, Zhen L (2014). Monodisperse sns_2_ nanosheets for high-performance photocatalytic hydrogen generation. ACS Appl. Mater. Interfaces..

[105] Lin C, Zhu X, Feng J, Wu C, Xie Y (2013). Hydrogen-incorporated TiS_2_ ultrathin nanosheets with ultrahigh conductivity for stamp-transferrable electrodes. J. Am. Chem. Soc..

[106] Sang YH, Zhao ZH, Zhao MW, Hao P, Leng YH, Liu H (2015). From UV to near-infrared, WS_2_ nanosheet: a novel photocatalyst for full solar light spectrum photodegradation. Adv. Mater..

[107] Wu Y, Xu M, Chen X, Yang S, Wu H, Pan J, Xiong X (2015). CTAB-assisted synthesis of novel ultrathin MoSe_2_ nanosheets perpendicular to graphene for adsorption and photodegradation of organic dyes under visible light. Nanoscale.

[108] Balendhran S, Walia S, Nili H, Ou JZ, Zhuiykov S (2013). Semiconductors: two-dimensional molybdenum trioxide and dichalcogenides. Adv. Funct. Mater..

[109] Chhowalla M, Shin HS, Eda G, Li LJ, Loh KP, Zhang H (2013). The chemistry of two-dimensional layered transition metal dichalcogenide nanosheets. Nat. Chem..

[110] Jang JT, Jeong S, Seo JW, Kim MC, Sim E (2011). Ultrathin zirconium disulfide nanodiscs. J. Am. Chem. Soc..

[111] Xie J, Zhang J, Shuang L, Grote F, Zhang X (2014). Correction to controllable disorder engineering in oxygen-incorporated MoS_2_ ultrathin nanosheets for efficient hydrogen evolution. J. Am. Chem. Soc..

[112] Hou J, Cao S, Wu Y, Liang F, Ye L, Lin Z, Sun L (2016). Perovskite-based nanocubes with simultaneously improved visible-light absorption and charge separation enabling efficient photocatalytic CO_2_ reduction. Nano Energy.

[113] Li WQ, Wang G, Zhang XN, Geng HP, Shen JL (2015). Geometrical and morphological optimizations of plasmonic nanoarrays for high-performance SERS detection. Nanoscale.

[114] Ablikim M, Fang SS, Yang HX, Zhao MG, Varner GS (2005). Observation of the decay ψ(2S) → k(892)k^−^+c.c. Phys. Lett. B.

[115] Di J, Xia J, Huang Y, Ji M, Fan W, Chen Z, Li H (2016). Constructing carbon quantum dots/Bi_2_SiO_5_ ultrathin nanosheets with enhanced photocatalytic activity and mechanism investigation. Chem. Eng. J..

[116] Liu H, Wang F, Liu L, Jia XY, Zheng W (2014). Synthesis, characterization, and ethylene polymerization behaviors of late-transition metal complexes coordinated with chlorinated bis(arylimino)pyridine ligand. Polymer.

[117] Tan L, Zhu XC, Tan MS, Sun L, Tan L (2014). The genetic variation of ARRB2 is associated with late-onset Alzheimer’s disease in Han Chinese. Curr. Alzheimer Res..

[118] Hameed A, Montini T, Gombac V, Fornasiero P (2008). Surface phases and photocatalytic activity correlation of Bi_2_O_3_/Bi_2_O_4-x_ nanocomposite. J. Am. Chem. Soc..

[119] Guan M, Xiao C, Zhang J, Fan S, Xie Y (2013). Vacancy associates promoting solar-driven photocatalytic activity of ultrathin bismuth oxychloride nanosheets. J. Am. Chem. Soc..

[120] Di J, Xia JX, Ji MX, Wang B, Yin S (2016). Advanced photocatalytic performance of graphene-like BN modified BIOBr flower-like materials for the removal of pollutants and mechanism insight. Appl. Catal. B Environ..

[121] Dreyer DR, Park S, Bielawski CW, Ruoff RS (2010). The chemistry of graphene oxide. Chem. Soc. Rev..

[122] Yeh TF, Syu JM, Cheng C, Chang TH, Teng H (2010). Graphite oxide as a photocatalyst for hydrogen production from water. Adv. Funct. Mater..

[123] Chu J, Sun J, Peng LI, Guangsheng LI, Niu Y (2016). Effect of platelet-rich plasma combined with human umbilical cord-mesenchymal stem cells on the healing of osteoporotic fracture in rats. Chin. J. Osteoporos..

[124] Zhou Z, Wang J, Yu J, Shen Y, Zhang Y (2015). Dissolution and liquid crystals phase of 2D polymeric carbon nitride. J. Am. Chem. Soc..

[125] Ping N, Zhang L, Liu G, Cheng HM (2012). Graphene-like carbon nitride nanosheets for improved photocatalytic activities. Adv. Funct. Mater..

[126] Yang S, Gong Y, Zhang J, Liang Z, Jayan PMA (2013). Exfoliated graphitic carbon nitride nanosheets as efficient catalysts for hydrogen evolution under visible light. Adv. Mater..

[127] Liu CX, Luo ZY, Li YW, Chen M, Xu J (2016). Active waveguides by low-fluence carbon implantation in Nd^3+^-doped fluorophosphate glasses. Mod. Phys. Lett. B.

[128] Xu H, Yan J, She X, Xu L, Xia J (2014). Graphene-analogue carbon nitride: novel exfoliation synthesis and its application in photocatalysis and photoelectrochemical selective detection of trace amount of Cu^2+^. Nanoscale.

[129] Zhang X, Xie X, Wang H, Zhang J, Pan B, Xie Y (2013). Enhanced photoresponsive ultrathin graphitic-phase C_3_N_4_ nanosheets for bioimaging. J. Am. Chem. Soc..

[130] Hevesi I, Nánai L, Vajtai R (1987). Laser light stimulated oxidation of vanadium at nonuniform illumination. Superlattice Microstruct..

[131] Ou H, Lin L, Zheng Y, Yang P, Fang Y, Wang X (2017). Tri-s-triazine-based crystalline carbon nitride nanosheets for an improved hydrogen evolution. Adv. Mater..

[132] Z.T.Z. Tao, K.W.K. Wang, F.Y.F. Yi, C.Y.C. Yan, Q.L.Q. Li et al., A 3D soc design for H.264 application with on-chip dram stacking, in *2010 IEEE International 3D Systems Integration Conference (3DIC)*, vol. 1, Corpus ID: 11735204 (2010). 10.1109/3DIC.2010.5751446

[133] Ryu J, Jang YJ, Choi S, Kang HJ, Park H, Lee JS, Park S (2016). All-in-one synthesis of mesoporous silicon nanosheets from natural clay and their applicability to hydrogen evolution. NPG Asia Mater..

[134] Xie YP, Xing J-Y, Li X-Y, Wang X, Sun H-J (2013). Survey of sweetpotato viruses in China. Acta Virol..

[135] Mashtalir O, Cook KM, Mochalin VN, Crowe M, Barsoum MW, Gogotsi Y (2014). Dye adsorption and decomposition on two-dimensional titanium carbide in aqueous media. J. Mater. Chem. A.

[136] Su J, Li GD, Li XH, Chen JS (2019). 2D/2D heterojunctions for catalysis. Adv. Sci..

[137] Maeda K, Eguchi M, Oshima T (2014). Perovskite oxide nanosheets with tunable band-edge potentials and high photocatalytic hydrogen-evolution activity. Angew. Chem. Int. Ed..

[138] Ngaw CK, Xu Q, Tan TTY, Hu P, Cao S, Loo JSC (2014). A strategy for in situ synthesis of well-defined core–shell Au@TiO_2_ hollow spheres for enhanced photocatalytic hydrogen evolution. Chem. Eng. J..

[139] Li J, Zhan G, Yu Y, Zhang L (2016). Superior visible light hydrogen evolution of Janus bilayer junctions via atomic-level charge flow steering. Nat. Commun..

[140] Wu Y, Li Z, Ma W, Huang Y, Huo L (2013). PDT-S-T: a new polymer with optimized molecular conformation for controlled aggregation and *π*–*π* stacking and its application in efficient photovoltaic devices. Adv. Mater..

[141] Chen EQ, Song XQ, Wang YL, Zhou TY, Bai L (2011). Construction of a highly-active, liver-specific transcriptional regulatory element through combination of the albumin promoter and α-fetoprotein enhancer. Plasmid.

[142] Deckoff-Jones S, Zhang J, Petoukhoff CE, Man MKL, Lei S (2016). Observing the interplay between surface and bulk optical nonlinearities in thin van der Waals crystals. Sci. Rep..

[143] Sun Y, Sun Z, Gao S, Cheng H, Liu Q (2014). Photoelectrochemical reactions: all-surface-atomic-metal chalcogenide sheets for high-efficiency visible-light photoelectrochemical water splitting. Adv. Energy Mater..

[144] Sun Y, Sun Z, Gao S, Cheng H, Liu Q (2014). All-surface-atomic-metal chalcogenide sheets for high-efficiency visible-light photoelectrochemical water splitting. Adv. Energy Mater..

[145] Liang D, Luo H, Liu YF, Hao ZY, Wang Y (2013). Lysilactones A–C, three 6H-dibenzo(*b*, *d*)pyran-6-one glycosides from *Lysimachia clethroides*, total synthesis of lysilactone A. Tetrahedron.

[146] Li Y, Wang Z, Xia T, Ju H, Zhang K (2016). Implementing metal-to-ligand charge transfer in organic semiconductor for improved visible-near-infrared photocatalysis. Adv. Mater..

[147] Lei F, Zhang L, Sun Y, Liang L, Liu K (2015). Atomic-layer-confined doping for atomic-level insights into visible-light water splitting. Angew. Chem. Int. Ed..

[148] Liu G, Niu P, Sun CH, Smith SC, Chen ZG, Lu GQ, Cheng HM (2010). Unique electronic structure induced high photoreactivity of sulfur-doped graphitic C_3_N_4_. J. Am. Chem. Soc..

[149] Zhou J, Huang Y, Cao X, Ouyang B, Sun W (2015). Two-dimensional NiCo_2_O_4_ nanosheet-coated three-dimensional graphene networks for high-rate, long-cycle-life supercapacitors. Nanoscale.

[150] Qiao Q, Li BH, Shan CX, Liu JS, Yu J (2012). Light-emitting diodes fabricated from small-size ZnO quantum dots. Mater. Lett..

[151] Huang C, Chen C, Zhang M, Lin L, Ye X (2015). Carbon-doped BN nanosheets for metal-free photoredox catalysis. Nat. Commun..

[152] Bi W, Ye C, Xiao C, Tong W, Zhang X, Shao W, Xie Y (2014). Spatial location engineering of oxygen vacancies for optimized photocatalytic. Small.

[153] Collaboration BES, Ablikim M, Bai JZ, Ban Y, Bian JG (2005). Observation of the decay (2S)k(892)k + c.c. Phys. Lett. B.

[154] Gao S, Gu B, Jiao X, Sun Y, Zu X (2017). Highly efficient and exceptionally durable CO_2_ photoreduction to methanol over freestanding defective single-unit-cell bismuth vanadate layers. J. Am. Chem. Soc..

[155] Cargnello M, Montini T, Smolin SY, Prieb JB, Delgado Jaén JJ (2016). Engineering titania nanostructure to tune and improve its photocatalytic activity. Proc. Natl. Acad. Sci..

[156] Du J, Zhang M, Guo Z, Chen J, Zhu X (2017). Phosphorene quantum dot saturable absorbers for ultrafast fiber lasers. Sci. Rep..

[157] Ge Y, Zhu Z, Xu Y, Chen Y, Chen S (2018). Broadband nonlinear photoresponse of 2D TiS_2_ for ultrashort pulse generation and all-optical thresholding devices. Adv. Opt. Mater..

[158] Guo B, Wang SH, Wu ZX, Wang ZX, Wang DH (2018). Sub-200 fs soliton mode-locked fiber laser based on bismuthene saturable absorber. Opt. Express.

[159] Jiang X, Liu S, Liang W, Luo S, He Z (2018). Broadband nonlinear photonics in few-layer MXene Ti_3_C_2_t_x_ (t = F, O, or OH). Laser Photonics Rev..

[160] Jiang X, Zhang L, Liu S, Zhang Y, He Z (2018). Ultrathin metal–organic framework: an emerging broadband nonlinear optical material for ultrafast photonics. Adv. Opt. Mater..

[161] Song Y, Liang Z, Jiang X, Chen Y, Li Z (2017). Few-layer antimonene decorated microfiber: ultra-short pulse generation and all-optical thresholding with enhanced long term stability. 2D Mater..

[162] Liu Z, Mu H, Xiao S, Wang R, Wang Z (2016). Pulsed lasers employing solution-processed plasmonic Cu_3−x_P colloidal nanocrystals. Adv. Mater..

[163] Zhu X, Chen S, Zhang M, Chen L, Wu Q (2018). TiS_2_-based saturable absorber for ultrafast fiber laser. Photonics Res..

[164] Li P, Chen Y, Yang T, Wang Z, Lin H (2017). Two-dimensional CH_3_NH_3_PBi_3_ perovskite nanosheets for ultrafast pulsed fiber lasers. ACS Appl. Mater. Interfaces..

[165] Zhang M, Wu Q, Zhang F, Chen L, Jin X (2018). 2D black phosphorus saturable absorbers for ultrafast photonics. Adv. Opt. Mater..

[166] Song YF, Zhang H, Tang DY, Shen DY (2012). Polarization rotation vector solitons in a graphene mode-locked fiber laser. Opt. Express.

[167] Chen Y, Wu M, Tang P, Chen S, Du J (2014). The formation of various multi-soliton patterns and noise-like pulse in a fiber laser passively mode-locked by a topological insulator based saturable absorber. Laser Phys. Lett..

[168] Ge Y, Chen S, Xu Y, He Z, Liang Z (2017). Few-layer selenium-doped black phosphorus: synthesis, nonlinear optical properties and ultrafast photonics applications. J. Mater. Chem. C.

[169] Li J, Luo H, Zhai B, Lu R, Guo Z, Zhang H, Liu Y (2016). Black phosphorus: a two-dimension saturable absorption material for mid-infrared q-switched and mode-locked fiber lasers. Sci. Rep..

[170] Liu M, Zhao N, Liu H, Zheng X, Luo A (2014). Dual-wavelength harmonically mode-locked fiber laser with topological insulator saturable absorber. IEEE Photonics Technol. Lett..

[171] Liu M, Cai ZR, Hu S, Luo AP, Zhao CJ (2015). Dissipative rogue waves induced by long-range chaotic multi-pulse interactions in a fiber laser with a topological insulator-deposited microfiber photonic device. Opt. Lett..

[172] Zheng G, Chen Y, Huang H, Zhao C, Lu S (2013). Improved transfer quality of CVD-grown graphene by ultrasonic processing of target substrates: applications for ultra-fast laser photonics. ACS Appl. Mater. Interfaces..

[173] Song YF, Zhang H, Zhao LM, Shen DY, Tang DY (2016). Coexistence and interaction of vector and bound vector solitons in a dispersion-managed fiber laser mode locked by graphene. Opt. Express.

[174] Song Y, Chen S, Zhang Q, Li L, Zhao L, Zhang H, Tang D (2016). Vector soliton fiber laser passively mode locked by few layer black phosphorus-based optical saturable absorber. Opt. Express.

[175] Luo ZC, Liu M, Guo ZN, Jiang XF, Luo AP (2015). Microfiber-based few-layer black phosphorus saturable absorber for ultra-fast fiber laser. Opt. Express.

[176] Wang Q, Chen Y, Miao L, Jiang G, Chen S (2015). Wide spectral and wavelength-tunable dissipative soliton fiber laser with topological insulator nano-sheets self-assembly films sandwiched by PMMA polymer. Opt. Express.

[177] Xu Y, Wang W, Ge Y, Guo H, Zhang X (2017). Stabilization of black phosphorous quantum dots in PMMA nanofiber film and broadband nonlinear optics and ultrafast photonics application. Adv. Funct. Mater..

[178] Jiang XF, Zeng Z, Li S, Guo Z, Zhang H, Huang F, Xu QH (2017). Tunable broadband nonlinear optical properties of black phosphorus quantum dots for femtosecond laser pulses. Materials (Basel).

[179] Wang Z, Xu Y, Dhanabalan SC, Sophia J, Zhao C (2016). Black phosphorus quantum dots as an efficient saturable absorber for bound soliton operation in an erbium doped fiber laser. IEEE Photonics J..

[180] Ma C, Wang C, Gao B, Adams J, Wu G, Zhang H (2019). Recent progress in ultrafast lasers based on 2d materials as a saturable absorber. Appl. Phys. Rev..

[181] Jiang T, Yin K, Wang C, You J, Ouyang H (2019). Ultrafast fiber lasers mode-locked by two-dimensional materials: review and prospect. Photonics Res..

[182] Song Y, Shi X, Wu C, Tang D, Zhang H (2019). Recent progress of study on optical solitons in fiber lasers. Appl. Phys. Rev..

[183] Fang Y, Ge Y, Wang C, Zhang H (2019). Mid-infrared photonics using 2D materials: status and challenges. Laser Photonics Rev..

[184] Zheng J, Tang X, Yang Z, Liang Z, Chen Y (2017). Few-layer phosphorene-decorated microfiber for all-optical thresholding and optical modulation. Adv. Opt. Mater..

[185] Zheng J, Yang Z, Si C, Liang Z, Chen X (2017). Black phosphorus based all-optical-signal-processing: toward high performances and enhanced stability. ACS Photonics.

[186] Wang C, Wang Y, Jiang X, Xu J, Huang W (2019). MXene Ti_3_C_2_T_x_: a promising photothermal conversion material and application in all-optical modulation and all-optical information loading. Adv. Opt. Mater..

[187] Wang Y, Huang W, Zhao J, Huang H, Wang C (2019). A bismuthene-based multifunctional all-optical phase and intensity modulator enabled by photothermal effect. J. Mater. Chem. C.

[188] Wu L, Huang W, Wang Y, Zhao J, Ma D (2019). 2d tellurium based high-performance all-optical nonlinear photonic devices. Adv. Funct. Mater..

[189] Chen S, Miao L, Chen X, Chen Y, Zhao C (2015). Few-layer topological insulator for all-optical signal processing using the nonlinear kerr effect. Adv. Opt. Mater..

[190] Song Y, Chen Y, Jiang X, Ge Y, Wang Y (2019). Nonlinear few-layer MXene-assisted all-optical wavelength conversion at telecommunication band. Adv. Opt. Mater..

[191] Wang Y, Zhang F, Tang X, Chen X, Chen Y (2018). All-optical phosphorene phase modulator with enhanced stability under ambient conditions. Laser Photonics Rev..

[192] Wu L, Chen K, Huang W, Lin Z, Zhao J (2018). Perovskite CSPBX_3_: a promising nonlinear optical material and its applications for ambient all-optical switching with enhanced stability. Adv. Opt. Mater..

[193] Wu L, Dong Y, Zhao J, Ma D, Huang W (2019). Kerr nonlinearity in 2D graphdiyne for passive photonic diodes. Adv. Mater..

[194] Wu L, Jiang X, Zhao J, Liang W, Li Z (2018). Mxene-based nonlinear optical information converter for all-optical modulator and switcher. Laser Photonics Rev..

[195] Wu L, Xie Z, Lu L, Zhao J, Wang Y (2018). Few-layer tin sulfide: a promising black-phosphorus-analogue 2D material with exceptionally large nonlinear optical response, high stability, and applications in all-optical switching and wavelength conversion. Adv. Opt. Mater..

[196] Wu Q, Chen S, Wang Y, Wu L, Jiang X (2019). MZI-based all-optical modulator using mxene Ti_3_C_2_T_x_ (T = F, O, or OH) deposited microfiber. Adv. Mater. Technol..

[197] Wang Y, Huang W, Wang C, Guo J, Zhang F (2019). An all-optical, actively q-switched fiber laser by an antimonene-based optical modulator. Laser Photonics Rev..

[198] Ou Q, Zhang Y, Wang Z, Yuwono JA, Wang R (2018). Strong depletion in hybrid perovskite p–n junctions induced by local electronic doping. Adv. Mater..

[199] Guo P, Xu J, Gong K, Shen X, Lu Y (2016). On-nanowire axial heterojunction design for high-performance photodetectors. ACS Nano.

[200] Guo Z, Chen S, Wang Z, Yang Z, Liu F (2017). Metal-ion-modified black phosphorus with enhanced stability and transistor performance. Adv. Mater..

[201] Huang Z, Han W, Tang H, Ren L, Chander DS, Qi X, Zhang H (2015). Photoelectrochemical-type sunlight photodetector based on MoS_2_/graphene heterostructure. 2D Mater..

[202] Ren X, Li Z, Huang Z, Sang D, Qiao H (2017). Environmentally robust black phosphorus nanosheets in solution: application for self-powered photodetector. Adv. Funct. Mater..

[203] Xu Y, Yuan J, Zhang K, Hou Y, Sun Q (2017). Field-induced n-doping of black phosphorus for CMOS compatible 2D logic electronics with high electron mobility. Adv. Funct. Mater..

[204] Ji X, Kong N, Wang J, Li W, Xiao Y (2018). A novel top-down synthesis of ultrathin 2D boron nanosheets for multimodal imaging-guided cancer therapy. Adv. Mater..

[205] Liang X, Ye X, Wang C, Xing C, Miao Q (2019). Photothermal cancer immunotherapy by erythrocyte membrane-coated black phosphorus formulation. J. Control. Release.

[206] Luo M, Fan T, Zhou Y, Zhang H, Mei L (2019). 2D black phosphorus-based biomedical applications. Adv. Funct. Mater..

[207] Sun ZB, Zhao YT, Li ZB, Cui HD, Zhou YY (2017). TiL_4_-coordinated black phosphorus quantum dots as an efficient contrast agent for in vivo photoacoustic imaging of cancer. Small.

[208] Qiu M, Wang D, Liang W, Liu L, Zhang Y (2018). Novel concept of the smart NIR-light-controlled drug release of black phosphorus nanostructure for cancer therapy. Proc. Natl. Acad. Sci..

[209] Yin F, Hu K, Chen S, Wang D, Zhang J (2017). Black phosphorus quantum dot based novel siRNA delivery systems in human pluripotent teratoma PA-1 cells. J. Mater. Chem. B.

[210] Fan T, Zhou Y, Qiu M, Zhang H (2018). Black phosphorus: a novel nanoplatform with potential in the field of bio-photonic nanomedicine. J. Innov. Opt. Health Sci..

[211] Tao W, Ji X, Xu X, Islam MA, Li Z (2017). Antimonene quantum dots: synthesis and application as near-infrared photothermal agents for effective cancer therapy. Angew. Chem. Int. Ed..

[212] Xue T, Liang W, Li Y, Sun Y, Xiang Y (2019). Ultrasensitive detection of miRNA with an antimonene-based surface plasmon resonance sensor. Nat. Commun..

[213] Xie H, Li Z, Sun Z, Shao J, Yu XF (2016). Metabolizable ultrathin Bi_2_Se_3_ nanosheets in imaging-guided photothermal therapy. Small.

[214] Tao W, Ji X, Zhu X, Li L, Wang J (2018). Two-dimensional antimonene-based photonic nanomedicine for cancer theranostics. Adv. Mater..

[215] Liu J, Liu Y, Liu N, Han Y, Zhang X (2015). Metal-free efficient photocatalyst for stable visible water splitting via a two-electron pathway. Science.

[216] Xia J, Di J, Li H, Xu H, Li H, Guo S (2016). Ionic liquid-induced strategy for carbon quantum dots/BiOx (x = Br, Cl) hybrid nanosheets with superior visible light-driven photocatalysis. Appl. Catal. B Environ..

[217] Xiang F, Nan S, Liu Y, Chen X, Zhou X (2017). Simultaneously enhanced stability and selectivity for propene epoxidation with H_2_ and O_2_ on Au catalysts supported on nano-crystalline mesoporous TS-1. ACS Catal..

[218] Qiao B, Wang A, Yang X, Allard LF, Jiang Z (2011). Single-atom catalysis of Co oxidation using Pt_1_/FeO_x_. Nat. Chem..

[219] Chen Z, Pronkin S, Fellinger TP, Kailasam K, Vilé G (2016). Merging single-atom-dispersed silver and carbon nitride to a joint electronic system via copolymerization with silver tricyanomethanide. ACS Nano.

[220] Li X, Bi W, Zhang L, Tao S, Xie Y (2016). Single-atom Pt as co-catalyst for enhanced photocatalytic H_2_ evolution. Adv. Mater..

[221] Ida S, Kim N, Ertekin E, Takenaka S, Ishihara T (2014). Photocatalytic reaction centers in two-dimensional titanium oxide crystals. J. Am. Chem. Soc..

[222] Han Z, Qiu F, Eisenberg R, Holland PL, Krauss TD (2012). Robust photogeneration of H_2_ in water using semiconductor nanocrystals and a nickel catalyst. Science.

[223] Lu X, Xu K, Tao S, Shao Z, Peng X (2016). Engineering the electronic structure of two-dimensional subnanopore nanosheets using molecular titanium-oxide incorporation for enhanced photocatalytic activity. Chem. Sci..

[224] Yuan YJ, Ye ZJ, Lu H, Hu B, Li YH (2016). Constructing anatase TiO_2_ nanosheets with exposed (001) facets/layered MoS_2_ two-dimensional nanojunction for enhanced solar hydrogen generation. ACS Catal..

[225] Ida S, Takashiba A, Koga S, Hagiwara H, Ishihara T (2014). Potential gradient and photocatalytic activity of an ultrathin p–n junction surface prepared with two-dimensional semiconducting nanocrystals. J. Am. Chem. Soc..

[226] Huang YZ, Wu LM, Wu XT, Li LH, Chen L, Zhang YF (2010). Pb_2_B_5_O_9_I: an iodide borate with strong second harmonic generation. J. Am. Chem. Soc..

[227] Hou Y, Laursen AB, Zhang J, Zhang G, Zhu Y (2013). Layered nanojunctions for hydrogen-evolution catalysis. Angew. Chem. Int. Ed..

[228] Huang YH, Wang JJ, Liu ZM, Lin GD, Zhang HB (2013). Highly efficient Ni–ZrO_2_ catalyst doped with YB_2_O_3_ for co-methanation of CO and CO_2_. Appl. Catal. A Gen..

[229] Wen F, An C, Wu X, Yang Y, Xu J (2018). MiR-34a regulates mitochondrial content and fat ectopic deposition induced by resistin through the AMPK/PPARα pathway in HepG2 cells. Int. J. Biochem. Cell Biol..

[230] Gunjakar JL, Kim TW, Kim HN, Kim IY, Hwang SJ (2011). Mesoporous layer-by-layer ordered nanohybrids of layered double hydroxide and layered metal oxide: highly active visible light photocatalysts with improved chemical stability. J. Am. Chem. Soc..

[231] Sun Y, Sun Z, Gao S, Cheng H, Liu Q (2012). Fabrication of flexible and freestanding zinc chalcogenide single layers. Nat. Commun..

[232] Liu Y, Liang L, Xiao C, Hua X, Li Z, Pan B, Xie Y (2016). Promoting photogenerated holes utilization in pore-rich WO_3_ ultrathin nanosheets for efficient oxygen-evolving photoanode. Adv. Energy Mater..

[233] Sun Y, Sun Z, Gao S, Cheng H, Liu Q (2012). Fabrication of flexible and freestanding zinc chalcogenide single layers. Nat. Commun..

[234] He Q, Li C, Geng F, Yang H, Li P (2012). Aerosol optical properties retrieved from sun photometer measurements over Shanghai, China. J. Geophys. Res. Atmos..

[235] Sun Y, Sun Z, Gao S, Cheng H, Liu Q (2014). Photoelectrochemical reactions: all-surface-atomic-metal chalcogenide sheets for high-efficiency visible-light photoelectrochemical water splitting. Adv. Energy Mater..

[236] Zhu J, Yin Z, Dan Y, Sun T, Yan Q (2013). Hierarchical hollow spheres composed of ultrathin Fe_2_O_3_ nanosheets for lithium storage and photocatalytic water oxidation. Energy Environ. Sci..

[237] Ablikim M, Bai JZ, Bai Y, Ban Y, Cai X (2008). Measurements of the observed cross sections for e^+^e^−^ → exclusive light hadrons containing π^0^π^0^ at √s = 3.773, 3.650 and 3.6648 GeV. Phys. Lett. B.

[238] Kwon KC, Choi S, Hong K, Andoshe DM, Suh JM (2017). Tungsten disulfide thin film/p-type si heterojunction photocathode for efficient photochemical hydrogen production. MRS Commun..

[239] Zhang M, Guan J, Tu Y, Chen S, Wang Y (2020). Highly efficient H_2_ production from H_2_S via a robust graphene-encapsulated metal catalyst. Energy Environ. Sci..

[240] Wang H, Peng R, Hood ZD, Naguib M, Adhikari SP, Wu Z (2016). Titania composites with 2D transition metal carbides as photocatalysts for hydrogen production under visible-light irradiation. Chemsuschem.

[241] Peng J, Chen X, Ong WJ, Zhao X, Li N (2019). Surface and heterointerface engineering of 2D MXenes and their nanocomposites: insights into electro- and photocatalysis. Chem.

[242] Su T, Peng R, Hood ZD, Naguib M, Ivanov IN, Keum JK, Qin Z, Guo Z, Wu Z (2018). One-step synthesis of Nb_2_O_5_/C/Nb_2_C (MXene) composites and their use as photocatalysts for hydrogen evolution. Chemsuschem.

[243] Ran J, Gao G, Li FT, Ma TY, Du A, Qiao SZ (2017). Ti_3_C_2_ Mxene co-catalyst on metal sulfide photo-absorbers for enhanced visible-light photocatalytic hydrogen production. Nat. Commun..

[244] Shao M, Shao Y, Chai JW, Qu Y, Pan H (2017). Synergistic effect of 2D Ti_2_C and g-C_3_N_4_ for efficient photocatalytic hydrogen production. J. Mater. Chem. A.

[245] Vasileff A, Xu C, Jiao Y, Zheng Y, Qiao SZ (2018). Surface and interface engineering in copper-based bimetallic materials for selective CO_2_ electroreduction. Chem.

[246] Liang L, Lei F, Gao S, Sun Y, Jiao X (2015). Single unit cell bismuth tungstate layers realizing robust solar CO_2_ reduction to methanol. Angew. Chem. Int. Ed..

[247] Asif S, Kashif R, Mohsin N, Waheed M, Jiseon J (2017). Heterostructural TiO_2_/Ti_3_C_2_T_x_ (MXene) for photocatalytic degradation of antiepileptic drug carbamazepine. Chem. Eng. J..

[248] Li N, Chen X, Ong WJ, MacFarlane DR, Zhao X, Cheetham AK, Sun C (2017). Understanding of electrochemical mechanisms for CO_2_ capture and conversion into hydrocarbon fuels in transition-metal carbides (MXenes). ACS Nano.

[249] Bai Y, Ye L, Chen T, Wang L, Shi X, Zhang X, Chen D (2016). Facet-dependent photocatalytic N_2_ fixation of bismuth-rich Bi_5_O_7i_ nanosheets. ACS Appl. Mater. Interfaces..

[250] Li H, Shang J, Ai Z, Zhang L (2015). Efficient visible light nitrogen fixation with BIOBr nanosheets of oxygen vacancies on the exposed 001 facets. J. Am. Chem. Soc..

[251] Cao S, Shen B, Tong T, Fu J, Yu J (2018). 2D/2D heterojunction of ultrathin mxene/Bi_2_Wo_6_ nanosheets for improved photocatalytic CO_2_ reduction. Adv. Funct. Mater..

[252] Ye M, Wang X, Liu E, Ye J, Wang D (2018). Boosting the photocatalytic activity of P25 for carbon dioxide reduction using a surface-alkalinized titanium carbide MXene as co-catalyst. Chemsuschem.

[253] Liu C, Xu Q, Zhang Q, Zhu Y, Xu J (2019). Layered BioBr/Ti_3_C_2_ MXene composite with improved visible-light photocatalytic activity. J. Mater. Sci..

[254] Kong XY, Tan WL, Ng BJ, Chai SP, Mohamed AR (2017). Harnessing Vis-NIR broad spectrum for photocatalytic CO_2_ reduction over carbon quantum dots-decorated ultrathin Bi_2_Wo_6_ nanosheets. Nano Res..

[255] Christianson JR, Zhu D, Hamers RJ, Schmidt JR (2014). Mechanism of N_2_ reduction to NH_3_ by aqueous solvated electrons. J. Phys. Chem. B.

[256] Li H, Qin F, Yang Z, Cui X, Wang J, Zhang L (2017). New reaction pathway induced by plasmon for selective benzyl alcohol oxidation on BioCl possessing oxygen vacancies. J. Am. Chem. Soc..

[257] Zhang N, Li X, Ye H, Chen S, Ju H (2016). Oxide defect engineering enables to couple solar energy into oxygen activation. J. Am. Chem. Soc..

[258] Li XB, Liu CH, Zhang R, Huang XT, Li YY (2013). Determination and pharmacokinetics of amygdalin in rats by LC-MS–MS. J. Chromatogr. Sci..

[259] Peng C, Yang X, Li Y, Yu H, Wang H, Peng F (2016). Hybrids of two-dimensional Ti_3_C_2_ and TiO_2_ exposing 001 facets toward enhanced photocatalytic activity. ACS Appl. Mater. Interfaces..

[260] Lin Z, Barbara D, Taberna PL, Van Aken KL, Anasori B, Gogotsi Y, Simon P (2016). Capacitance of Ti_3_C_2_t_x_ mxene in ionic liquid electrolyte. J. Power Sources.

[261] Xie X, Zhang N, Tang ZR, Anpo M, Xu YJ (2018). Ti_3_C_2_T_x_ MXene as a janus cocatalyst for concurrent promoted photoactivity and inhibited photocorrosion. Appl. Catal. B Environ..

[262] Wang H, Wu Y, Xiao T, Yuan X, Zeng G (2018). Formation of quasi-core–shell In_2_S_3_/anatase TiO_2_ @metallic Ti_3_C_2_t_x_ hybrids with favorable charge transfer channels for excellent visible-light-photocatalytic performance. Appl. Catal. B Environ..

[263] Campos-Martin JM, Blanco-Brieva G, Fierro JL (2006). Hydrogen peroxide synthesis: an outlook beyond the anthraquinone process. Angew. Chem. Int. Ed..

[264] Sato K, Aoki M, Noyori R (1998). A “Green” Route to adipic acid: direct oxidation of cyclohexenes with 30 percent hydrogen peroxide. Science.

[265] Yang S, Verdaguer-Casadevall A, Arnarson L, Silvioli L, Čolić V (2018). Toward the decentralized electrochemical production of H_2_O_2_: a focus on the catalysis. ACS Catal..

[266] Zhan W, Ji L, Ge ZM, Wang X, Li RT (2018). A continuous-flow synthesis of primary amides from hydrolysis of nitriles using hydrogen peroxide as oxidant. Tetrahedron.

[267] Ksibi M (2006). Chemical oxidation with hydrogen peroxide for domestic wastewater treatment. Chem. Eng. J..

[268] Gurram RN, Al-Shannag M, Lecher NJ, Duncan SM, Singsaas EL, Alkasrawi M (2015). Bioconversion of paper mill sludge to bioethanol in the presence of accelerants or hydrogen peroxide pretreatment. Bioresour. Technol..

[269] Mase K, Yoneda M, Yamada Y, Fukuzumi S (2016). Seawater usable for production and consumption of hydrogen peroxide as a solar fuel. Nat. Commun..

[270] Lan YQ, Wang XL, Dong LZ, Man Q, Su JX (2018). Exploring the performance improvement of oxygen evolution reaction in stable bimetal–organic framework system. Angew. Chem. Int. Ed..

[271] Zhang Z, Liu K, Feng Z, Bao Y, Dong B (2016). Hierarchical sheet-on-sheet ZnIn_2_S_4_/g-C_3_N_4_ heterostructure with highly efficient photocatalytic H_2_ production based on photoinduced interfacial charge transfer. Sci. Rep..

[272] Liu CZ, Zhang YF, Li XF, Lu XF, Chang Z (2020). The high energy X-ray telescope (HE) onboard the insight-HXMT astronomy satellite. Sci. China Phys. Mech..

[273] Zhong M, Hisatomi T, Kuang Y, Zhao J, Liu M (2015). Surface modification of CoO_x_ loaded BiVo_4_ photoanodes with ultrathin p-type NiO layers for improved solar water oxidation. J. Am. Chem. Soc..

[274] Wang L, Zheng X, Chen L, Xiong Y, Xu H (2018). Van der Waals heterostructures comprised of ultrathin polymer nanosheets for efficient z-scheme overall water splitting. Angew. Chem. Int. Ed..

[275] Zhu M, Kim S, Mao L, Fujitsuka M, Zhang J, Wang X, Majima T (2017). Metal-free photocatalyst for H_2_ evolution in visible to near-infrared region: black phosphorus/graphitic carbon nitride. J. Am. Chem. Soc..

[276] Ran J, Guo W, Wang H, Zhu B, Yu J, Qiao SZ (2018). Metal-free 2D/2D phosphorene/g-C_3_N_4_ van der Waals heterojunction for highly enhanced visible-light photocatalytic h_2_ production. Adv. Mater..

[277] Gai Q, Zheng X, Liu W, Dong Q, Wang Y, Gao R, Ren S (2019). 2D–2D heterostructured CdS–CoP photocatalysts for efficient H2 evolution under visible light irradiation. Int. J. Hydrog. Energy.

[278] Sasikala R, Gaikwad A, Jayakumar O, Girija K, Rao R, Tyagi A, Bharadwaj S (2015). Nanohybrid MoS_2_-PANI-CdS photocatalyst for hydrogen evolution from water. Colloids Surf. A.

[279] Luo M, Yao W, Huang C, Wu Q, Xu Q (2015). Shape effects of pt nanoparticles on hydrogen production via Pt/CdS photocatalysts under visible light. J. Mater. Chem A.

[280] Lang D, Shen T, Xiang Q (2015). Roles of MoS_2_ and graphene as cocatalysts in the enhanced visible-light photocatalytic H_2_ production activity of multiarmed CdS nanorods. ChemCatChem.

[281] Jiang W, Liu Y, Zong R, Li Z, Yao W, Zhu Y (2015). Photocatalytic hydrogen generation on bifunctional ternary heterostructured In_2_S_3_/MoS_2_/CdS composites with high activity and stability under visible light irradiation. J. Mater. Chem. A.

[282] Jia T, Kolpin A, Ma C, Chan RCT, Kwok WM, Tsang SE (2014). A graphene dispersed CdS–MoS_2_ nanocrystal ensemble for cooperative photocatalytic hydrogen production from water. Chem. Commun..

[283] Chang K, Mei Z, Wang T, Kang Q, Ouyang S, Ye J (2014). MoS_2_/graphene cocatalyst for efficient photocatalytic H_2_ evolution under visible light irradiation. ACS Nano.

[284] Chang K, Li M, Wang T, Ouyang S, Li P, Liu L, Ye J (2015). Drastic layer number dependent activity enhancement in photocatalytic H_2_ evolution over nMoS_2_/CdS (n ≥ 1) under visible light. Adv. Energy Mater..

[285] Zong X, Wu G, Yan H, Ma G, Shi J (2010). Photocatalytic H_2_ evolution on MoS_2_/CdS catalysts under visible light irradiation. J. Phys. Chem. C.

[286] Zong X, Yan H, Wu G, Ma G, Wen F, Wang L, Li C (2008). Enhancement of photocatalytic H_2_ evolution on CdS by loading MoS_2_ as cocatalyst under visible light irradiation. J. Am. Chem. Soc..

[287] Ma S, Xie J, Wen J, He K, Li X, Liu W, Zhang X (2017). Constructing 2D layered hybrid CdS nanosheets/mos_2_ heterojunctions for enhanced visible-light photocatalytic H_2_ generation. Appl. Surf. Sci..

[288] Wang X, Maeda K, Thomas A, Takanabe K, Xin G (2009). A metal-free polymeric photocatalyst for hydrogen production from water under visible light. Nat. Mater..

[289] Jia L, Wang DH, Huang YX, Xu AW, Yu HQ (2011). Highly durable n-doped graphene/cds nanocomposites with enhanced photocatalytic hydrogen evolution from water under visible light irradiation. J. Phys. Chem. C.

[290] Maitra U, Gupta U, De M, Datta R, Govindaraj A, Rao C (2013). Highly effective visible-light-induced H_2_ generation by single-layer 1T-MoS_2_ and a nanocomposite of few-layer 2H-MoS_2_ with heavily nitrogenated graphene. Angew. Chem. Int. Ed..

[291] Gupta U, Naidu B, Maitra U, Singh A, Shirodkar SN, Waghmare UV, Rao C (2014). Characterization of few-layer 1T-MoSe_2_ and its superior performance in the visible-light induced hydrogen evolution reaction. APL Mater..

[292] Mahler B, Hoepfner V, Liao K, Ozin GA (2014). Colloidal synthesis of 1T-WS_2_ and 2H-WS_2_ nanosheets: applications for photocatalytic hydrogen evolution. J. Am. Chem. Soc..

[293] Chen J, Wu XJ, Yin L, Li B, Hong X (2015). One-pot synthesis of CdS nanocrystals hybridized with single-layer transition-metal dichalcogenide nanosheets for efficient photocatalytic hydrogen evolution. Angew. Chem. Int. Ed..

[294] Lei Z, You W, Liu M, Zhou G, Takata T (2003). Photocatalytic water reduction under visible light on a novel ZnIn_2_S_4_ catalyst synthesized by hydrothermal method. Chem. Commun..

[295] Xu Z, Li Y, Peng S, Lu G, Li S (2012). Nacl-assisted low temperature synthesis of layered Zn–In–S photocatalyst with high visible-light activity for hydrogen evolution. RSC Adv..

[296] Yu J, Qi L, Jaroniec M (2010). Hydrogen production by photocatalytic water splitting over Pt/TiO_2_ nanosheets with exposed (001) facets. J. Phys. Chem. C.

[297] Zhang K, Fujitsuka M, Du Y, Majima T (2018). 2D/2D heterostructured CdS/WS_2_ with efficient charge separation improving H_2_ evolution under visible light irradiation. ACS Appl. Mater. Interfaces..

[298] Schwarzenbach RP, Egli T, Hofstetter TB, Gunten UV, Wehrli B (2010). Global water pollution and human health. Annu. Rev. Environ. Resour..

[299] Li Y, Li K, Yang Y, Li L, Xing Y (2015). Ultrathin g-C_3_N_4_ nanosheets coupled with AgIO_3_ as highly efficient heterostructured photocatalysts for enhanced visible-light photocatalytic activity. Chemistry.

[300] Wang J, Tang L, Zeng G, Deng Y, Liu Y (2017). Atomic scale g-C_3_N_4_/Bi_2_WO_6_ 2D/2D heterojunction with enhanced photocatalytic degradation of ibuprofen under visible light irradiation. Appl. Catal. B Environ..

[301] Bera R, Kundu S, Patra A (2015). 2D hybrid nanostructure of reduced graphene oxide–CdS nanosheet for enhanced photocatalysis. ACS Appl. Mater. Interfaces..

[302] Liu Y, Xie S, Hui L, Wang X (2014). A highly efficient sunlight driven ZnO nanosheet photocatalyst: synergetic effect of p-doping and MoS_2_ atomic layer loading. ChemCatChem.

[303] Ong WJ, Tan LL, Chai SP, Yong ST (2015). Graphene oxide as a structure-directing agent for the two-dimensional interface engineering of sandwich-like graphene-g-C_3_N_4_ hybrid nanostructures with enhanced visible-light photoreduction of CO_2_ to methane. Chem. Commun..

[304] Sun J, Zhang H, Guo LH, Zhao L (2013). Two-dimensional interface engineering of a titania–graphene nanosheet composite for improved photocatalytic activity. ACS Appl. Mater. Interfaces..

[305] Ong W, Tan LL, Chai SP, Yong ST, Mohamed A (2015). Surface charge modification via protonation of graphitic carbon nitride (g-C_3_N_4_) for electrostatic self-assembly construction of 2D/2D reduced graphene oxide (rGO)/g-C_3_N_4_ nanostructures toward enhanced photocatalytic reduction of carbon dioxide to methane. Nano Energy.

[306] Liang YT, Vijayan BK, Lyandres O, Gray KA, Hersam MC (2012). Effect of dimensionality on the photocatalytic behavior of carbon-titania nanosheet composites: charge transfer at nanomaterial interfaces. J. Phys. Chem. Lett..

[307] Tu W, Zhou Y, Liu Q, Tian Z, Gao J (2012). Robust hollow spheres consisting of alternating titania nanosheets and graphene nanosheets with high photocatalytic activity for CO_2_ conversion into renewable fuels. Adv. Funct. Mater..

[308] Chen X, Zhou Y, Liu Q, Li Z, Liu J, Zou Z (2012). Ultrathin, single-crystal WO_3_ nanosheets by two-dimensional oriented attachment toward enhanced photocatalystic reduction of CO_2_ into hydrocarbon fuels under visible light. ACS Appl. Mater. Interfaces..

[309] Tu W, Li Y, Kuai L, Zhou Y, Xu Q (2017). Construction of unique two-dimensional MoS_2_–TiO_2_ hybrid nanojunctions: MoS_2_ as a promising cost-effective cocatalyst toward improved photocatalytic reduction of CO_2_ to methanol. Nanoscale.

[310] Zhang L, Wang W, Jiang D, Gao E, Sun S (2015). Photoreduction of CO_2_ on BioCl nanoplates with the assistance of photoinduced oxygen vacancies. Nano Res..

[311] Wang JC, Yao HC, Fan ZY, Zhang L, Wang JS, Zang SQ, Li ZJ (2016). Indirect z-scheme BiOi/g-C_3_N_4_ photocatalysts with enhanced photoreduction CO_2_ activity under visible light irradiation. ACS Appl. Mater. Interfaces..

[312] Kawamura S, Puscasu MC, Yoshida Y, Izumi Y, Carja G (2015). Tailoring assemblies of plasmonic silver/gold and zinc–gallium layered double hydroxides for photocatalytic conversion of carbon dioxide using UV–visible light. Appl. Catal. A Gen..

[313] Hsu HC, Shown I, Wei HY, Chang YC, Du HY (2013). Graphene oxide as a promising photocatalyst for CO_2_ to methanol conversion. Nanoscale.

[314] Qiu B, Li Q, Shen B, Xing M, Zhang J (2016). Stöber-like method to synthesize ultradispersed Fe_3_O_4_ nanoparticles on graphene with excellent photo-fenton reaction and high-performance lithium storage. Appl. Catal. B Environ..

[315] Tan LL, Ong WJ, Chai SP, Goh BT, Mohamed AR (2015). Visible-light-active oxygen-rich TiO_2_ decorated 2D graphene oxide with enhanced photocatalytic activity toward carbon dioxide reduction. Appl. Catal. B Environ..

[316] Ali A, Oh WC (2017). A simple ultrasono-synthetic route of PbSe–graphene–TiO_2_ ternary composites to improve the photocatalytic reduction of CO_2_. Fuller. Nanotube Carbon Nanostruct..

[317] Tan LL, Ong WJ, Chai SP, Mohamed AR (2013). Reduced graphene oxide–TiO_2_ nanocomposite as a promising visible-light-active photocatalyst for the conversion of carbon dioxide. Nanoscale Res. Lett..

[318] Wang A, Li X, Zhao Y, Wu W, Chen J, Meng H (2014). Preparation and characterizations of Cu_2_O/reduced graphene oxide nanocomposites with high photo-catalytic performances. Powder Technol..

[319] Xu YF, Yang MZ, Chen BX, Wang XD, Chen HY, Kuang DB, Su CY (2017). A CsPbBr_3_ perovskite quantum dot/graphene oxide composite for photocatalytic co_2_ reduction. J. Am. Chem. Soc..

[320] Kuai L, Zhou Y, Tu W, Li P, Li H (2015). Rational construction of a CdS/reduced graphene oxide/TiO_2_ core-shell nanostructure as an all-solid-state z-scheme system for CO_2_ photoreduction into solar fuels. RSC Adv..

[321] Dai W, Yu J, Deng Y, Hu X, Wang T, Luo X (2017). Facile synthesis of MoS_2_/Bi_2_WO_6_ nanocomposites for enhanced CO_2_ photoreduction activity under visible light irradiation. Appl. Surf. Sci..

[322] Jiao X, Li X, Jin X, Sun Y, Xu J (2017). Partially oxidized sns_2_ atomic layers achieving efficient visible-light-driven CO_2_ reduction. J. Am. Chem. Soc..

[323] Xin Q, Shah H, Nawaz A, Xie W, Akram MZ (2019). Antibacterial carbon-based nanomaterials. Adv. Mater..

[324] Ge J, Zhang Y, Park SJ (2019). Recent advances in carbonaceous photocatalysts with enhanced photocatalytic performances: a mini review. Materials.

[325] Wang R, Zhang X, Li F, Cao D, Pu M (2018). Energy-level dependent H_2_O_2_ production on metal-free, carbon-content tunable carbon nitride photocatalysts. J. Energy Chem..

[326] Tasis D, Tagmatarchis N, Bianco A, Prato M (2006). Chemistry of carbon nanotubes. Chem. Rev..

[327] Zhao S, Guo T, Li X, Xu T, Yang B, Zhao X (2018). Carbon nanotubes covalent combined with graphitic carbon nitride for photocatalytic hydrogen peroxide production under visible light. Appl. Catal. B Environ..

[328] Long DL, Burkholder E, Cronin L (2007). Polyoxometalate clusters, nanostructures and materials: from self assembly to designer materials and devices. Chem. Soc. Rev..

[329] Han XB, Zhang ZM, Zhang T, Li YG, Lin W (2014). Polyoxometalate-based cobalt–phosphate molecular catalysts for visible light-driven water oxidation. J. Am. Chem. Soc..

[330] Kong XJ, Lin Z, Zhang ZM, Zhang T, Lin W (2016). Hierarchical integration of photosensitizing metal–organic frameworks and nickel-containing polyoxometalates for efficient visible-light-driven hydrogen evolution. Angew. Chem. Int. Ed..

[331] Zhou J, Chen W, Sun C, Han L, Qin C (2017). Oxidative polyoxometalates modified graphitic carbon nitride for visible-light CO_2_ reduction. ACS Appl. Mater. Interfaces..

[332] Zhao S, Zhao X, Zhang H, Li J, Zhu Y (2017). Covalent combination of polyoxometalate and graphitic carbon nitride for light-driven hydrogen peroxide production. Nano Energy.

[333] Zhao S, Zhao X, Ouyang S, Zhu Y (2018). Polyoxometalates covalently combined with graphitic carbon nitride for photocatalytic hydrogen peroxide production. Catal. Sci. Technol..

[334] Zhao S, Zhao X (2018). Polyoxometalates-derived metal oxides incorporated into graphitic carbon nitride framework for photocatalytic hydrogen peroxide production under visible light. J. Catal..

[335] Zhao S, Zhao X (2019). Insights into the role of singlet oxygen in the photocatalytic hydrogen peroxide production over polyoxometalates-derived metal oxides incorporated into graphitic carbon nitride framework. Appl. Catal. B Environ..

[336] Jiang X, Wang P, Zhao J (2015). 2d covalent triazine framework: a new class of organic photocatalyst for water splitting. J. Mater. Chem. A.

[337] Shiraishi Y, Kanazawa S, Kofuji Y, Sakamoto H, Ichikawa S, Tanaka S, Hirai T (2014). Sunlight-driven hydrogen peroxide production from water and molecular oxygen by metal-free photocatalysts. Angew. Chem. Int. Ed..

[338] Kofuji Y, Ohkita S, Shiraishi Y, Sakamoto H, Tanaka S, Ichikawa S, Hirai T (2016). Graphitic carbon nitride doped with biphenyl diimide: efficient photocatalyst for hydrogen peroxide production from water and molecular oxygen by sunlight. ACS Catal..

[339] Kofuji Y, Ohkita S, Shiraishi Y, Sakamoto H, Ichikawa S, Tanaka S, Hirai T (2017). Mellitic triimide-doped carbon nitride as sunlight-driven photocatalysts for hydrogen peroxide production. Catal. Sci. Technol..

[340] Kofuji Y, Isobe Y, Shiraishi Y, Sakamoto H, Tanaka S, Ichikawa S, Hirai T (2016). Carbon nitride–aromatic diimide–graphene nanohybrids: metal-free photocatalysts for solar-to-hydrogen peroxide energy conversion with 0.2% efficiency. J. Am. Chem. Soc..

[341] Kofuji Y, Isobe Y, Shiraishi Y, Sakamoto H, Ichikawa S, Tanaka S, Hirai T (2018). Hydrogen peroxide production on a carbon nitride–boron nitride-reduced graphene oxide hybrid photocatalyst under visible light. ChemCatChem.

[342] Yang L, Dong G, Jacobs DL, Wang Y, Zang L, Wang C (2017). Two-channel photocatalytic production of H_2_O_2_ over g-C_3_N_4_ nanosheets modified with perylene imides. J. Catal..

[343] Kim HI, Choi Y, Hu S, Choi W, Kim JH (2018). Photocatalytic hydrogen peroxide production by anthraquinone-augmented polymeric carbon nitride. Appl. Catal. B Environ..

[344] Xue F, Si Y, Wang M, Liu M, Guo L (2019). Toward efficient photocatalytic pure water splitting for simultaneous H_2_ and H_2_O_2_ production. Nano Energy.

[345] Shiraishi Y, Kanazawa S, Sugano Y, Tsukamoto D, Sakamoto H, Ichikawa S, Hirai T (2014). Highly selective production of hydrogen peroxide on graphitic carbon nitride (g-C_3_N_4_) photocatalyst activated by visible light. ACS Catal..

[346] Burek BO, Bahnemann DW, Bloh JZ (2018). Modeling and optimization of the photocatalytic reduction of molecular oxygen to hydrogen peroxide over titanium dioxide. ACS Catal..

[347] Cai R, Kubota Y, Fujishima A (2003). Effect of copper ions on the formation of hydrogen peroxide from photocatalytic titanium dioxide particles. J. Catal..

[348] Kim S, Moon GH, Kim G, Kang U, Park H, Choi W (2017). TiO_2_ complexed with dopamine-derived polymers and the visible light photocatalytic activities for water pollutants. J. Catal..

[349] Zeng X, Wang Z, Wang G, Gengenbach TR, McCarthy DT (2017). Highly dispersed TiO_2_ nanocrystals and wo_3_ nanorods on reduced graphene oxide: Z-scheme photocatalysis system for accelerated photocatalytic water disinfection. Appl. Catal. B Environ..

[350] Xiong X, Zhang X, Liu S, Zhao J, Xu Y (2018). Sustained production of H_2_O_2_ in alkaline water solution using borate and phosphate-modified Au/TiO_2_ photocatalysts. Photochem. Photobiol. Sci..

[351] Zuo G, Li B, Guo Z, Wang L, Yang F (2019). Efficient photocatalytic hydrogen peroxide production over TiO_2_ passivated by SnO_2_. Catalysts.

[352] Shiraishi Y, Kofuji Y, Sakamoto H, Tanaka S, Ichikawa S, Hirai T (2015). Effects of surface defects on photocatalytic H_2_O_2_ production by mesoporous graphitic carbon nitride under visible light irradiation. ACS Catal..

[353] Kim S, Moon GH, Kim H, Mun Y, Zhang P, Lee J, Choi W (2018). Selective charge transfer to dioxygen on KPF6-modified carbon nitride for photocatalytic synthesis of H_2_O_2_ under visible light. J. Catal..

[354] Shi L, Yang L, Zhou W, Liu Y, Yin L (2018). Photoassisted construction of holey defective g-C_3_N_4_ photocatalysts for efficient visible-light-driven H_2_O_2_ production. Small.

[355] Wei Z, Liu M, Zhang Z, Yao W, Tan H, Zhu Y (2018). Efficient visible-light-driven selective oxygen reduction to hydrogen peroxide by oxygen-enriched graphitic carbon nitride polymers. Energy Environ. Sci..

[356] Kusuyama Y, Tanaka S, Sakatsuji K, Nishihara T, Saito K (2008). Central pontine myelinolysis. Pathol. Int..

[357] Zhao S, Wang X, Huo M (2010). Catalytic wet air oxidation of phenol with air and micellar molybdovanadophosphoric polyoxometalates under room condition. Appl. Catal. B Environ..

[358] Kato S, Jung J, Suenobu T, Fukuzumi S (2013). Production of hydrogen peroxide as a sustainable solar fuel from water and dioxygen. Energy Environ. Sci..

[359] Hirakawa H, Shiota S, Shiraishi Y, Sakamoto H, Ichikawa S, Hirai T (2016). Au nanoparticles supported on BiVO_4_: effective inorganic photocatalysts for H_2_O_2_ production from water and O_2_ under visible light. ACS Catal..

[360] Jie X, Chen Z, Zhang H, Lin G, Lin H, Wang X, Long J (2017). Cd_3_(C_3_N_3_S_3_)_2_ coordination polymer/graphene nanoarchitectures for enhanced photocatalytic H_2_O_2_ production under visible light. Sci. Bull..

[361] Inagami T, Yokosawa N, Takahashi N, Takii Y (1979). Partial purification of prorenin and activation by kallikreins: a possible new link between renin and kallikrein systems. Adv. Exp. Med. Biol..

